# Radiation with reproductive isolation in the near-absence of phylogenetic signal

**DOI:** 10.1126/sciadv.adt0973

**Published:** 2025-07-25

**Authors:** Martin Helmkampf, Floriane Coulmance, Melanie J. Heckwolf, Arturo Acero P., Alice Balard, Iliana Bista, Omar Dominguez Dominguez, Paul B. Frandsen, Montserrat Torres-Oliva, Aintzane Santaquiteria, Jose Tavera, Benjamin C. Victor, D. Ross Robertson, Ricardo Betancur-R., W. Owen McMillan, Oscar Puebla

**Affiliations:** ^1^ Leibniz Center for Tropical Marine Research (ZMT), Bremen, Germany.; ^2^ Institute for Chemistry and Biology of the Marine Environment (ICBM), Carl von Ossietzky Universität Oldenburg, Oldenburg, Germany.; ^3^ Smithsonian Tropical Research Institute (STRI), Panama, Republic of Panama.; ^4^ Instituto para el Estudio de las Ciencias del Mar (CECIMAR), Universidad Nacional de Colombia sede Caribe, Santa Marta, Colombia.; ^5^ Corporation Center of Excellence in Marine Science (CEMarin), Bogota, Colombia.; ^6^ Division of Biosciences, Faculty of Life Science, University College London (UCL), London, UK.; ^7^ Senckenberg Research Institute and Natural History Museum, Frankfurt am Main, Germany.; ^8^ LOEWE Centre for Translational Biodiversity Genomics, Frankfurt am Main, Germany.; ^9^ Tree of Life, Wellcome Sanger Institute, Wellcome Genome Campus, Cambridge, UK.; ^10^ Laboratorio de Biología Acuática, Facultad de Biología, Universidad Michoacana de San Nicolás de Hidalgo, Morelia, Michoacán, México.; ^11^ Laboratorio Nacional de Análisis y Síntesis Ecológica para la Conservación de Recursos Genéticos de México, Escuela Nacional de Estudios Superiores, Unidad Morelia, Universidad Nacional Autónoma de México, Morelia, Michoacán, México.; ^12^ Department of Plant and Wildlife Sciences, Brigham Young University, Provo, UT, USA.; ^13^ Institute of Clinical Molecular Biology (IKMB), Kiel University, Kiel, Germany.; ^14^ School of Biological Sciences, The University of Oklahoma, Norman, OK, USA.; ^15^ Departamento de Biología, Universidad del Valle, Cali, Colombia.; ^16^ Ocean Science Foundation, Irvine, CA, USA.; ^17^ Guy Harvey Research Institute, Dania Beach, FL, USA.; ^18^ Scripps Institution of Oceanography, University of California San Diego, San Diego, CA, USA.

## Abstract

According to the genic view, species are characterized by the genes that underlie functional divergence. Here, we take a phylogenomic approach to assess this view at the scale of a whole radiation. The hamlets (*Hypoplectrus* spp.) represent a recent radiation of reef fishes from the Greater Caribbean that are reproductively isolated through assortative mating. A total of 335 genomes from 15 locations revealed a single well-supported phylogenetic split among species, with a large share of the radiation unresolved. The polytomic nature of the hamlet radiation is extreme compared to other recent radiations such as Lake Victoria cichlids. At the gene-tree level, we identified just one genomic region, centered around the *casz1* transcription factor, with a topology that reflects species differences. These results show that phenotypic diversification and reproductive isolation—two major attributes of species—may unfold in the near-absence of phylogenetic signal, both genome-wide and at the gene-tree level.

## Teaser

Phenotypic diversification and reproductive isolation may unfold in the near-absence of phylogenetic signal.

## INTRODUCTION

Species are the foundational units of biodiversity, but how they form and what exactly characterizes them remain open questions. Furthermore, few theoretical frameworks and empirical systems provide the opportunity to address these two questions jointly. In this regard, the genic view of species and speciation (hereafter “genic view”) is of particular interest. This framework was articulated by Wu with a specific focus on animals (*1*, *2*). However, the idea predates Wu (*3*, *4*), also applies to plants (*5*), and is not necessarily incompatible with alternative views of species and speciation (*5*–*7*). A fundamental tenet of the genic view is that species are essentially formed and characterized by the genes that underlie adaptation to ecological and sexual environments, with reproductive isolation emerging as a by-product of this functional divergence. From a genomic perspective, it is in the presence of gene flow that the genic view manifests itself most clearly because gene flow tends to reveal the genomic regions underlying functional divergence by homogenizing genetic variation throughout the rest of the genome. Nevertheless, an understanding of the nature and origin of species requires more than just a genomic perspective. It also entails the knowledge of the traits that underlie adaptation, how they relate to ecological and sexual environments, and how this functional divergence results in reproductive isolation.

The hamlets (*Hypoplectrus* spp., Serranidae) provide a rare opportunity to empirically assess the genic view. These reef fishes from the Greater Caribbean differ mainly in terms of color pattern (*8*), which is ecologically relevant for camouflage and mimicry (*9*–*13*), but are otherwise morphologically and ecologically very similar (*14*, *15*). They also differ in terms of distribution and abundance (*16*–*21*) but are highly sympatric, with up to nine species encountered on a single dive survey (*8*). Hamlet courtship and spawning behavior can be observed on a daily basis throughout the year, which provides a direct window on prezygotic reproductive isolation. Different species are commonly observed spawning at the same time and in the same area, but spawning is strongly assortative with respect to color pattern (*11*, *12*, *16*, *22*, *23*). Assortative mate choice is maintained under experimental conditions and also when fishes are kept in different compartments without water exchange (*16*), indicating that it relies heavily on visual cues. Interspecific spawnings are nonetheless observed at a low frequency (<2%) in natural populations (*11*, *12*, *16*, *22*, *23*). There are no barriers to fertilization among species (*24*), and the available evidence indicates that hybrids develop normally. In the few cases where hybrids were raised to the adult stage, they developed color patterns that were intermediate between the two parental species (*16*). Furthermore, high-probability hybrids and backcrosses have been identified in natural populations through genetic analysis (*25*–*27*), indicating that hybridization and introgression are ongoing.

**Fig. 1.**
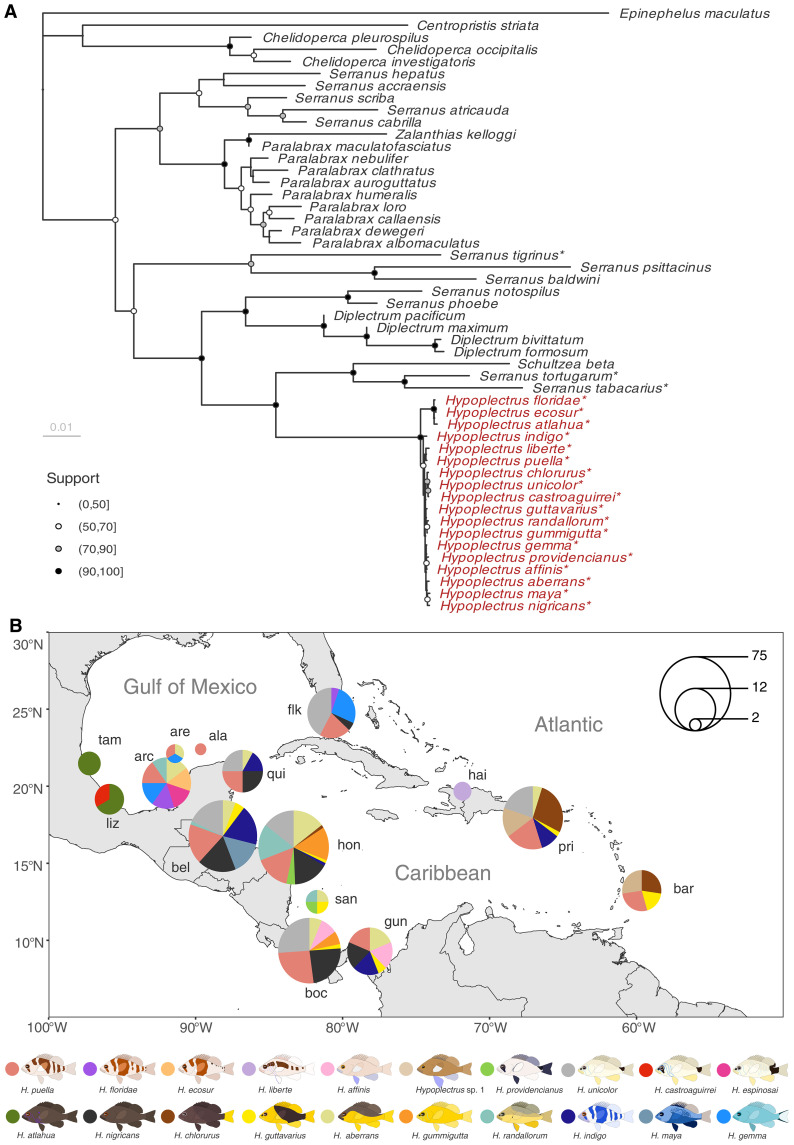
Phylogenetic context of the hamlet radiation and sampling design. (**A**) Maximum likelihood phylogeny of the Serraninae subfamily based on 23 nuclear and mitochondrial genes. The hamlet radiation (genus *Hypoplectrus*) is highlighted in red, and the species considered in this study are marked with an asterisk. Gene sequences for the other species were obtained from the Fish Tree of Life (*64*). Node point size and fill represent node support derived from 200 nonparametric bootstrap replicates. (**B**) Sampling design. The dataset consists of 327 hamlet (*Hypoplectrus*) and eight *Serranus* outgroup genomes that include all described hamlet species from 15 locations covering the Gulf of Mexico [Tamiahua (tam), Antón Lizardo (liz), Cayos Arcas (arc), Cayo Arenas (are), Alacranes Reef (ala), and the Florida Keys (flk)], the Western Caribbean [Quintana Roo (qui), Belize (bel), Honduras (hon), San Andrés (san), Bocas del Toro (boc), and Guna Yala (gun)], and the Eastern Caribbean [Haiti (hai), Puerto Rico (pri), and Barbados (bar)].

In agreement with the occurrence of gene flow, hamlet species are genetically highly similar. Levels of genetic differentiation among species fall within the range that is typically encountered among populations within species (*12*, *23*, *28*–*31*). The genomic architecture of species differences is characterized by sharp peaks of differentiation that stand out against a background of low differentiation (*25*, *26*), exactly as envisioned by the genic view during the early stages of speciation (*1*, *2*). As a group, the hamlets form an exceptionally shallow radiation of 19 described species (Fig. 1A) that appear to have diverged very recently [<10,000 generations (*26*)] and are largely unresolved from a phylogenetic perspective (*26*, *28*, *32*–*35*). This raises the question whether phenotypic diversification and reproductive isolation, two major attributes of species, may unfold in the absence of phylogenetic signal. Note that we refer to the “phylogenetic signal” in the sense of phylogenetic informativeness, i.e., power of a gene or fraction of the genome to resolve phylogenetic relationships [e.g., (*36*, *37*)], as opposed to its meaning in the context of trait evolution [e.g., (*38*)]. Following the genic view, a small part of the genome—the genomic regions underlying functional divergence—is expected to show an evolutionary history that reflects species differences. The simplest scenario would be that a single genomic region phylogenetically resolves the whole radiation, but given the modular genetic basis of species differences in the hamlets (*26*, *27*), a more likely scenario is that different genomic regions resolve different aspects of species differences (pattern, color, ...), and that these regions jointly resolve the radiation. Nevertheless, previous phylogenetic studies of the hamlet radiation were based on a small number of markers and/or a limited subset of the species collected at few locations (*26*, *28*, *32*–*35*).

Here, we use genome-wide data to (i) provide a first comprehensive phylogenomic perspective of the hamlet radiation, (ii) explore whether diversification may develop in a genomic context that is overwhelmingly dominated by incomplete lineage sorting and introgression, and (iii) provide an illustration of how species may arise and persist against a backdrop of minimal genetic divergence. Following the genic view, we hypothesized that specific genomic regions may exhibit a phylogenetic signal that relates to species differences and that these genomic regions may point to the genomic basis of functional divergence among species.

## RESULTS

### The hamlet radiation is characterized by a single deep phylogenetic split

**Fig. 2.**
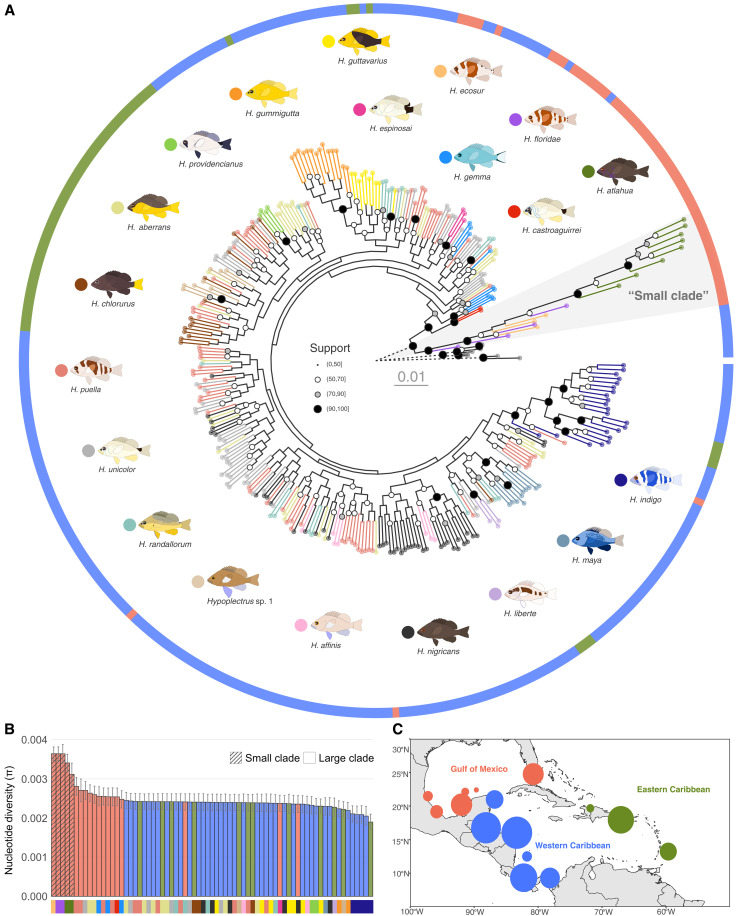
Phylogeny and nucleotide diversity of the hamlet radiation. (**A**) IQ-TREE phylogeny based on ~110,000 SNP markers distributed across the genome. Branch and tip point colors indicate the hamlet species as shown by the icons surrounding the tree, and outer ring colors correspond to the three biogeographic regions depicted in (C). Node point size and fill represent node support. The eight *Serranus* outgroup individuals are shown in gray, and the length of their branches, represented with dashed lines, is reduced to 5% of their actual length to improve the visualization of the hamlet radiation. (**B**) Nucleotide diversity (π) of the 71 populations (species/location combinations) considered in this study. Bar colors correspond to the three biogeographic regions depicted in (C), and *x*-axis labels indicate species as in (A). Small clade species are additionally highlighted with a hatched pattern. Bar height and errors bars represent the means and standard deviation, respectively, across the 24 LGs, for each of which (π) was calculated separately. (**C**) Map showing the grouping of the 15 locations into three biogeographic regions: Gulf of Mexico (orange), Western Caribbean (blue), and Eastern Caribbean (green).

To test our hypothesis, we compiled a dataset of 335 genomes including all recognized hamlet species from 15 locations across their geographical range (Fig. 1B, fig. S1, and table S1) and generated genome-wide phylogenies using three *Serranus* species as an outgroup. Multiple phylogenetic reconstruction methods based on different datasets identified just one consistent and well-supported phylogenetic split among species in the whole radiation. This split separated three species from the Gulf of Mexico (*Hypoplectrus floridae*, *Hypoplectrus ecosur*, and *Hypoplectrus atlahua*, hereafter “small clade”) from the rest of the radiation (hereafter “large clade”; Fig. 2A and figs. S2 and S3, also apparent in Fig. 1A). Using the multiple sequentially Markovian coalescent [MSMC2; (*39*)], we estimated that these two lineages started to diverge 60,000 generations ago (fig. S4). The three species constituting the small clade are largely restricted to the Gulf of Mexico, but the phylogenetic split does not reflect complete geographic isolation. Several species from the large clade are also present in the Gulf of Mexico, often in sympatry with members of the small clade (Fig. 2, A and C).

The phylogenetic split between the two clades was also recovered in the mitochondrial genome (fig. S5). However, we identified two individuals with a nuclear genome from the large clade and a mitochondrial genome from the small clade and two individuals with the opposite pattern. These mitonuclear discordances suggest that mitonuclear incompatibilities do not play a major role in the isolation of the two clades and more generally in reproductive isolation. They also point to introgression between the two clades. Analyses of single-nucleotide polymorphism (SNP)–derived ancestry proportions (*40*) and chromosome-scale ancestral recombination graphs (*41*) indicate that the two clades are admixed (fig. S6). Admixture is largely restricted to the Gulf of Mexico, where the two clades are found in sympatry. This geographic pattern suggests that gene flow and introgression, not just incomplete lineage sorting, contribute to admixture. The *D*-statistics and related estimates (*42*) confirm that introgression occurred between the two clades (fig. S7). Furthermore, demographic inference with linked selection using approximate Bayesian computation (*43*) indicates that the genomic data are more consistent with a scenario of continuous gene flow between the two clades—i.e., nonallopatric divergence—than scenarios without gene flow or with secondary contact (table S4).

Thus, the hamlet radiation is characterized by a single deep phylogenetic split that distinguishes three species from the Gulf of Mexico from the rest of the radiation, but these two clades are not completely geographically and genetically isolated. Another notable aspect of this split is that the species forming the small clade, in particular *H. floridae* and *H. ecosur*, present the highest levels of nucleotide diversity (π) in the whole radiation (Fig. 2B). This suggests that the hamlet radiation originated in the Gulf of Mexico from a hamlet population similar to *H. floridae* and *H. ecosur*. Furthermore, maximum likelihood biogeographic analysis indicates that despite the lack of phylogenetic signal for species boundaries, the dataset is well structured biogeographically (Gulf of Mexico, Western Caribbean, and Eastern Caribbean), and that the large clade originated in the Gulf of Mexico (fig. S8 and table S5). The emerging picture is therefore an origin of the hamlet radiation in the Gulf of Mexico, where two major lineages diverged with continuous gene flow, followed by expansion and further diversification of the large clade in the Caribbean.

### A large share of the radiation is unresolved at the genome-wide level

In contrast to the major phylogenetic split described above, none of the other deep nodes of the radiation were highly supported or consistently recovered by the different phylogenetic reconstruction methods that we implemented. Even at the shallow levels of the radiation, just 7 or 8 of the 19 described species were recovered as strictly monophyletic lineages with high support by the different methods applied to the nuclear genome (and this number may have been inflated by a small sample size for some of these species) and none according to the mitochondrial genome. The species recovered as strictly monophyletic with high support include members of both clades, with a high proportion of microendemic species (*H. atlahua*, *Hypoplectrus castroaguirrei*, *Hypoplectrus maya*, and *Hypoplectrus liberte*). These species are endemic to specific areas (*35*, *44*–*46*), a single bay in the extreme case of *H. liberte* (*34*). This suggests that increased genetic drift and lineage sorting because of low population size contributed to their divergence. The same may be said about geographic isolation, but to a limited extent because similar to the small clade in the Gulf of Mexico, microendemic species are often found in sympatry with other hamlets.

On the other hand, *Hypoplectrus indigo* and *Hypoplectrus gummigutta*, two species that are extensively distributed across the Greater Caribbean, were often recovered as monophyletic and were the most genetically distinct species of the large clade according to branch lengths (Fig. 2A and figs. S2 and S3). These two species are characterized by bright blue and yellow/orange colors, respectively, which are thought to be particularly effective for camouflage and communication in reef fishes (*47*). It is tempting to speculate that stronger natural and sexual selection contributed to the increased divergence of these two species, but they do not show stronger prezygotic isolation than the other hamlets (*12*, *23*). Four populations were also recovered as monophyletic lineages. These include a tan-colored hamlet from Barbados whose taxonomic status is unclear [*Hypoplectrus* sp. 1 (*8*)], *Hypoplectrus nigricans* from Bocas del Toro in Panama, *Hypoplectrus randallorum* from Cayo Arcas in the Gulf of Mexico, and *Hypoplectrus gemma* from the Florida Keys. Unsupervised clustering confirmed in large part the phylogenetic analyses by identifying up to six unambiguous genotypic clusters in the dataset, corresponding to *H. atlahua*, *H. gummigutta*, *H. indigo*, *H. maya*, *Hypoplectrus* sp. 1, and *H. nigricans* from Bocas del Toro (fig. S9). This indicates that at least from a genetic perspective, it may be justified to describe the latter two as species.

All in all, at best, just 8 of the 19 currently recognized species were recovered as monophyletic lineages, and even then, their phylogenetic relationships are largely unresolved because the deeper nodes of the radiation have low support values. Phylogenetic resolution did not noticeably improve as we considered more SNPs or genomic windows, and the general outcome was robust to all the SNP and genomic window selection strategies we tried for phylogenetic reconstruction. This leaves at least 11 species that are phylogenetically unresolved at the whole-genome level and notably includes *Hypoplectrus puella*, *H. nigricans*, and *Hypoplectrus unicolor*, the three most abundant and widely distributed species in the whole radiation. As illustrated by the SVDQuartets tree (fig. S3), the hamlet phylogeny is in large part a polytomy. Ancient polytomies are well documented (*48*–*50*), but the hamlet polytomy extends largely until present. Following the genic view, we hypothesized that specific genomic regions may be characterized by a phylogenetic signal that relates to species differences and that these genomic regions may point to the genomic basis of functional divergence.

### A single genomic region relates to species differences

To explore the phylogeny of specific genomic regions, we first examined the 2000 regions considered for the window-based phylogenetic inference (fig. S2). We hypothesized that windows with higher mean support might resolve species better, but this was not the case. All the species were mixed, and none were resolved in the region-specific phylogeny with the highest mean support (fig. S10), illustrating the complexity of region-specific phylogenies. This complexity, coupled with the large number of possible species/location topologies and the fact that a large proportion of the region-specific phylogenies that we examined did not align with species/location boundaries, precluded the use of a topology-based approach to analyze the region-specific phylogenies.

**Fig. 3.**
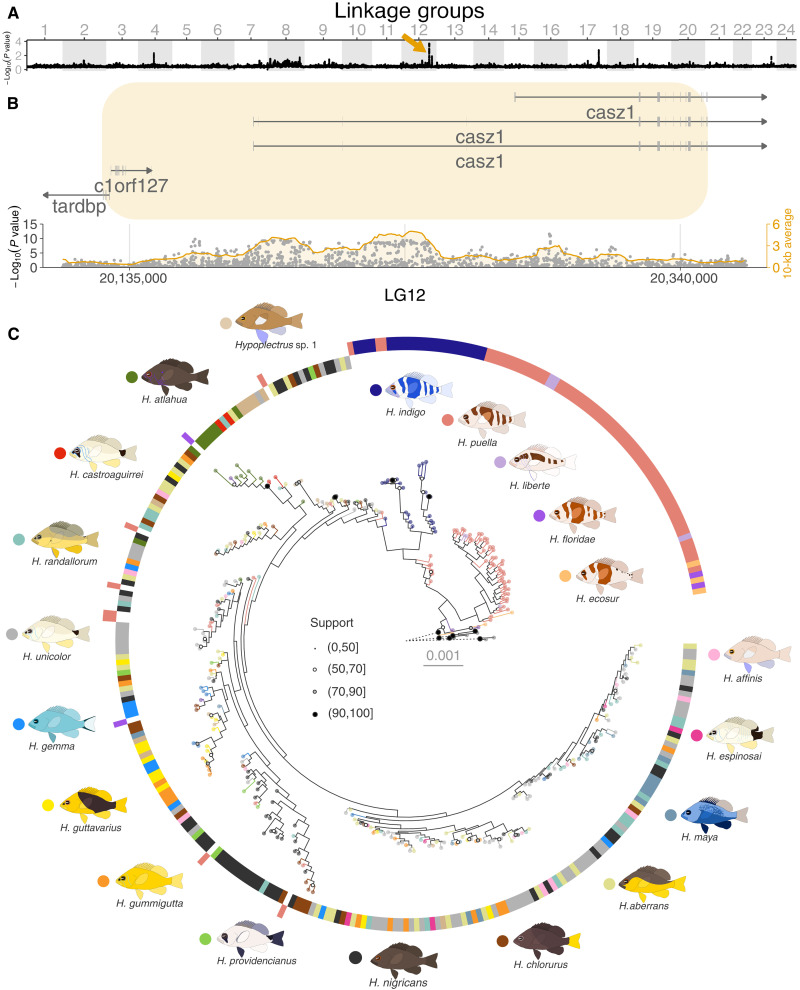
GWAS for species identity and *casz1* gene tree. (**A**) Results of the GWAS for species identity. The gray and white blocks represent the 24 LGs (putative chromosomes), and the black dots are averages of the GWAS log-transformed *P* value over 50-kb windows with 5-kb increments. (**B**) Close-up on the major association peak in the *casz1* genomic region on LG12. The gray dots represent the GWAS log-transformed *P* value for each individual SNP, and the orange line represents its average over 10-kb windows. The *x* axis shows the position on LG12 (in base pairs), and the orange rectangle highlights the region that shows an association >1.5 considering 50-kb windows. (**C**) Phylogeny of the *casz1* gene region highlighted in orange in (B), which spans 0.2 Mb. Ring, branch, and tip point colors indicate the hamlet species as shown by the icons surrounding the tree. The outer ring corresponds to species that have a barred color pattern and the inner ring the species that have a nonbarred color pattern. Node point size and fill represent node support. The eight *Serranus* outgroup samples are shown in gray, and the length of their branches, represented with dashed lines, is reduced to 5% of their actual length to improve the visualization of the hamlet radiation. The same pattern was obtained when considering the *casz1* gene only (fig. S11). The phylogenies of the other major association peaks are shown in figs. S13 to S19.

We then posited that if a genomic region resolves species or groups of species better than the rest of the genome, it is expected to show an increased association with species identity. On the basis of this principle, we conducted a genome-wide association study (GWAS) on species identity to uncover such regions. In agreement with previous association studies on a subset of the radiation (*26*, *27*), the results revealed sharp association peaks along the genome (Fig. 3A). The number of association peaks recovered by the GWAS is remarkably small considering that this analysis integrates the differences among all the described species. This confirms that a small number of large-effect loci contribute to the hamlet radiation.

In particular, one major association peak was recovered in the *casz1* gene region of linkage group (putative chromosome) 12 (LG12) (Fig. 3B). The phylogeny of this region failed to resolve any of the species with high bootstrap support, but it distinguishes the species that have brown vertical bars (*H. floridae*, *H. ecosur*, *H. puella*, and *H. liberte*), blue vertical bars (*H. indigo*), and no vertical bars (Fig. 3C). The same pattern was observed when considering the *casz1* gene only (fig. S11), but it faded away when only exons were considered (fig. S12), indicating that this signal is essentially noncoding. The branching pattern suggests a scenario in which the ancestral hamlets had brown vertical bars, from which blue-barred and nonbarred color patterns evolved and diversified rapidly. This is consistent with the high levels of nucleotide diversity of *H. floridae* and *H. ecosur*, which have brown vertical bars (Fig. 2B), and with the observation that most members of the subfamily Serraninae display cryptic color patterns such as brown vertical bars.

While the gene genealogy of the *casz1* region is notable, it is nonetheless characterized by moderate to low bootstrap support values and it is not perfectly clear-cut, with seven *H. puella* and two *H. floridae* samples grouping with nonbarred samples (Fig. 3C). The phylogenies of the other major association peaks are even more diffuse (figs. S13 to S19). Some of these local phylogenies recover the small and large clades or the most diverged species, but beyond that, they do not clearly relate to species, geography, or color pattern, except for a few trends. For example, the association peak on LG04 tends to group samples from the species that are blue together (*H. indigo*, *H. maya*, and *H. gemma*; fig. S13). This is consistent with previous analyses showing that this genomic region is associated with body color (*27*). One genomic region on LG08 tends to cluster *H. unicolor* and *Hypoplectrus espinosai* individuals, which are white (fig. S14), and two genomic regions on LG19 and LG23 tend to group *H. nigricans* samples, which are black (figs. S18 and S19). Some regions also show reduced genetic variation, with basically the same haplotype shared by several individuals from different species and locations (e.g., the association peak on LG17, which contains short- and long-wave sensitive opsin genes; fig. S17). In these cases, selection is likely to be the cause of the association peak. Species are broadly better recovered when considering the eight high-association regions jointly (fig. S20), but here again, the deep nodes have low support values. Just four species are recovered as monophyletic in this tree (*Hypoplectrus guttavarius*, *H. maya*, *H. gemma*, and *H. liberte*), and among these, only *H. gemma* with high bootstrap support.

In summary, we identified a single genomic interval with a phylogenetic signal that relates to species differences. This region is centered around the *casz1* gene, and its tree topology is linked to color pattern, the major functional trait of the radiation. *casz1* encodes a zinc-finger transcription factor that orchestrates cell differentiation in retinal and neural progenitors, in T helper cells, and during cardiac morphogenesis (*51*–*54*), suggesting a role as a housekeeping gene. In line with this, we found *casz1* to be coexpressed with a large number of genes in the skin, retina, and brain, three tissues that relate to the formation and perception of color pattern (fig. S21). *casz1* expression was orders of magnitude higher in the retina than in the skin and brain (fig. S22). This is consistent with the fact that *casz1* plays a role in photoreceptor development (*51*, *52*, *55*) and that the visual system of fishes grows continuously and therefore expresses developmental genes. The role of *casz1* for the establishment and maintenance of photoreceptor gene expression profiles (*51*, *52*, *55*) led us to look into the genes involved in the phototransduction cascade specifically, and we found that a large share of them was coexpressed with *casz1* in the retina (fig. S23). Furthermore, some of these genes, such as *PDE6* and *RDH*, were also coexpressed in the skin and brain in nonvisual contexts (fig. S23), suggesting possible pleiotropic effects of *casz1* in different tissues. Considering that the association peak is centered around the *casz1* transcription start site (Fig. 3B), we hypothesize that species differences may be driven by regulatory mechanisms. We found no differential expression or splicing of *casz1* among species (figs. S22 and S24), but this may occur in specific contexts, e.g., during development or mate choice. We also examined the expression of transposable elements in the *casz1* region and found neither differential expression among species (fig. S25) nor elevated transposon expression compared to the rest of the genome (fig. S26). Last, we assembled hamlet genomes de novo using long-read sequencing to look into structural variation in the *casz1* region and identified no major structural differences among species (fig. S27). Together, considering the association of *casz1* with color pattern and its strong expression in the retina, we speculate that it might pleiotropically regulate both the color pattern and mate choice. This would provide a mechanism for the explosive radiation of the hamlets, paralleling the situation in *Heliconius* butterflies where a gene linked to male approach behavior is in close proximity to a gene that modulates color pattern (*56*, *57*).

## DISCUSSION

We took a phylogenomic perspective to address the genic view of species and speciation at the scale of a whole radiation and uncovered just one well-supported phylogenetic split among species and few unequivocally monophyletic species. Thus, a large share of the hamlet radiation is phylogenetically unresolved, even with genome-wide data and state-of-the-art phylogenetic reconstruction methods. The whole-genome phylogenies were based on a fraction of the genome, yet it is unlikely that we missed any genomic region with a strong phylogenetic signal because the GWAS considered the whole genome. This lack of phylogenetic signal is notable considering that the hamlets present clear phenotypic differences among species and are reproductively isolated to a large extent through assortative mating. In comparison, the cichlids from Lake Victoria, the most rapid east African cichlid radiation and one of the few other systems that compare to the hamlets in terms of speciation rate, can be phylogenetically resolved with a fraction of the amount of data that we used (*58*). However, the observation that genome-wide data do not resolve species well should not come as a surprise under the view that species are essentially formed and characterized by the genes that underlie adaptation to ecological and sexual environments, which may represent a small fraction of the genome. This was recently illustrated by another notable case, an instance of homoploid hybrid speciation in *Heliconius* butterflies where one of the two parental species contributed just 1% of the genome that includes genomic regions linked to functional divergence (*59*).

Following the genic view, we hypothesized that the genomic regions underlying functional divergence would show a phylogenetic signal that relates to species differences, and this is exactly what we found for the *casz1* region that distinguishes brown-barred, blue-barred, and nonbarred species. Nevertheless, this was less clearly or not the case at all for the other genomic regions that we identified. These results are consistent with a recent high-resolution analysis of color pattern variation in a subset of the radiation (*27*), which indicates that the presence/absence of vertical bars is essentially a discrete trait that associates almost exclusively with the *casz1* region, while other traits such as body color vary more continuously and associate with several genomic regions. Many of the regions that associate with body color were identified by our GWAS on species identity but did not show a phylogenetic signal that clearly reflects species differences. The fact that the presence/absence of vertical bars is a discrete trait that is essentially governed by a single locus explains why this is the only genomic region that exhibits a phylogenetic signal that reflects species differences. Under polygenic control, as seems to be the case for body color and other species differences, phylogenetic patterns are expected to be diffuse at each individual genomic region because variation at other loci may also affect the trait either individually or in interaction. Thus, our results are consistent with the genic view, but they illustrate that while phylogenetic patterns are clear-cut when functional divergence is controlled by a single locus, they erode when the traits underlying functional divergence have a polygenic basis. This aligns with the observation that species are broadly better recovered when considering the eight high-association regions jointly, but the fact that this phylogeny still poorly recovers species suggests that large-effect genes are only part of the story. Furthermore, recurrent utilization of the same genomic regions might also contribute to the blur of the phylogenetic signal. Considering that many functional traits may have a polygenic basis (*60*), the near-absence of phylogenetic signal at the gene-tree level should not come as a surprise either. An even more extreme situation in which phenotypic diversification and reproductive isolation develop in the total absence of any phylogenetic signal, both genome-wide and at the gene-tree level, does not seem far-fetched and should actually be the default expectation. Our results highlight the limits of the phylogenomic approach to address adaptation, speciation, and rapid radiation at both the species and trait levels.

An in-depth discussion as to whether the hamlets should be considered species is provided in the latest taxonomic review of the group (*8*). For all practical purposes, they are recognized as species by the ichthyological community and beyond, but this ultimately depends on the species definition that is considered. In light of our results, one may be tempted to conclude that there are just two hamlet species corresponding to the two lineages that we identified, but this view would be completely at odds with the patterns of assortative mating and color variation in the group. In this regard, it is worth noting that although the genic view is not a species definition, the genomic regions that underlie functional divergence and the occurrence of linkage disequilibrium among these regions (*25*) may be used to identify species, which relates to the genotypic cluster species definition (*3*). Anyhow, the hamlet radiation is characterized by phenotypic differentiation and strong reproductive isolation, two fundamental attributes of species. We show herein that these attributes may unfold and persist in the near-absence of phylogenetic signal, and this conclusion remains valid regardless of the species status of the hamlets. This implies that the phylogenetic signal that we observe in older radiations may have largely developed after phenotypic diversification and prezygotic reproductive isolation, with little relation to the initial evolution of these traits.

## MATERIALS AND METHODS

### Sample collection and sequencing

This study is based on a total of 335 genomes from 327 hamlet (*Hypoplectrus* spp.) and 8 outgroup (*Serranus* spp.) samples (table S1). This constitutes a major improvement over the last genome-wide phylogenetic analysis of the hamlets that included just eight species from three Western Caribbean locations (*26*). The samples were collected between 2004 and 2017 across 15 locations that cover nearly the entire range of the hamlet radiation and include the 19 currently recognized hamlet species (Fig. 1B, fig. S1, and table S1). Some of these genomes were available from previous studies (*8*, *25*–*27*), three had been sequenced previously but were not used until this study, and 104 were sequenced anew for this study. Genomic DNA of these was extracted from fin clips stored in 100% ethanol at −20°C with the MagAttract high-molecular-weight DNA extraction kit (Qiagen). Illumina DNA Prep libraries were prepared and sequenced on three NovaSeq6000 S4 lanes [2 × 151 base pairs (bp)] at the Institute of Clinical Molecular Biology (IKMB, Kiel University).

In addition, genomes of three hamlet species were assembled de novo to look into structural variation within the *casz1* region (table S2). High-molecular-weight genomic DNA was extracted from gill tissue using the Qiagen genomic tip DNA extraction kit. DNA was sheared to 18 kb using a Diagenode Megaruptor and size selected for fragments >10 kb on a SAGE BluePippin. PacBio CCS (circular consensus sequencing) libraries were prepared according to the SMRTbell Express Template Prep Kit 2.0. Libraries were then sequenced at the DNA Sequencing Center (DNASC) at Brigham Young University on a single 30-hour SMRT cell on the PacBio Sequel II instrument in CCS mode (table S2).

Last, gene expression was analyzed from 99 tissue samples. These include 24 previously published retina samples (*25*), as well as 54 and 21 newly sequenced brain and skin samples, respectively (table S3). These samples included three hamlet species (*H. puella*, *H. nigricans*, and *H. unicolor*), collected in reefs around Isla Colón (Bocas del Toro, Panama). The collection and dissection procedures were randomized and standardized to minimize bias. For the skin, a 1 by 1–cm square was dissected from the area where the barred species have their central bar, with the upper edge parallel to the lateral line. The brain was dissected into three brain regions (telencephalon, diencephalon, and optic tectum), which were individually stored, extracted, and sequenced, resulting in three brain samples per individual. RNA was extracted from brain and skin tissue using the PureLink RNA Mini Kit with an additional TRIzol lysis step (Invitrogen by Thermo Fisher Scientific). Sequencing libraries were prepared using the Illumina TruSeq Stranded mRNA HT Sample Prep Kit Illumina, which includes a polyA enrichment step for mRNA purification, and sequenced on an Illumina NovaSeq platform (2 × 100 bp) at the IKMB (table S3).

### Computational analyses

The following data processing steps and analyses were performed on the high-performance computing cluster ROSA (University of Oldenburg) using Linux shell or Python scripts and Slurm version 23.02.4, unless noted otherwise. Results were visualized locally in R version 3.4.1 using ggplot version 3.4.4 and related packages.

### Genotyping

Previously available and new datasets were genotyped together using GATK version 4.1.9 (*61*). After marking adapters for removal, datasets were individually mapped to the *H. puella* reference genome assembly (*25*) with bwa version 0.7.17. Read data of the same sample sequenced across multiple lanes were merged, duplicates were marked, and samples with a mean coverage of less than 10× were removed from further analysis (the coverage of all samples after filtering averaged 22×). After calculating haplotype likelihoods, per-sample VCF files were combined into a single cohort GVCF file, and genotypes called jointly on all 24 reference LGs (putative chromosomes). During this step, the workflow was split into parallel tracks including all samples (phylo) and hamlet samples only (phyps). In both cases, only variant sites were called (snp), in addition to all callable sites except indels (all). These four datasets were then hard-filtered on the basis of missing data (10% cutoff) and various quality metrics as recommended by the Broad Institute for germline short variants. Last, the SNP datasets were limited to biallelic sites with a minor allele count of at least 2 using VCFtools version 0.1.16 (*62*). Where applicable, linked sites were removed by either applying a physical distance filter of 5 kb, beyond which very little physical linkage remains in the hamlets (*25*, *44*), or accepting a maximum correlation coefficient of 0.5 along 50-kb windows by a combination of VCFtools and BCFtools. A phased version of the phylo-snp dataset (without minor allele count filter) was produced on the basis of phase-informative reads with extractPIRs version 1 and SHAPEIT version 2 (*63*).

### Phylogenetic context

Before examining our whole-genome data in detail, we put the radiation into a broader taxonomic and phylogenetic context. First, we extracted the sequences of 23 nuclear and mitochondrial genes used by Fish Tree of Life (*64*) from the phylo-all dataset (or regenotyped contigs where necessary). For each of the 19 described hamlet species, the sample with the highest coverage was chosen. These sequences were then combined with the corresponding sequences of the Serraninae subfamily represented in FToL. Alignment and phylogenetic inference followed (*26*), with the exception of using 200 nonparametric bootstrap replicates.

### Species tree inference

Multiple genome-wide, conventional, and coalescent-aware approaches were pursued to reconstruct the phylogeny of the hamlet radiation itself. First, 110,436 genome-wide SNPs—the phylo-snp dataset filtered by physical linkage—were converted to Fasta format and treated as a conventional alignment containing 70,505 parsimony-informative sites. A thorough nearest-neighbor interchange search was conducted with IQ-TREE version 2.2.2.7 (*65*) under the GTR + ASC model for ascertainment bias correction (*66*). Branch support values were obtained by ultrafast bootstrap approximation (*67*) with 1000 nearest-neighbor interchange–optimized replicates.

Second, the same dataset was converted to Nexus format and subjected to an SVDQuartets (*68*) analysis implemented in PAUP version 4.0a (*69*). The optimal quartet-based tree was identified by considering 490 million quartets (95% of the total number of distinct quartets). In the case of heterozygous (ambiguous) sites, counts were distributed over all compatible site patterns. A 50% majority consensus tree was calculated from 200 bootstrap replicates each considering 5 million (1%) quartets.

Third, 2000 nonoverlapping windows of 5-kb near-continuous genomic sequence were randomly extracted from the phylo-all dataset. A window size of 5 kb was considered because very little linkage remains beyond this distance in the hamlets (*25*, *44*). Extracted sites were converted to Fasta format using a custom Perl script (https://github.com/JinfengChen/vcf-tab-to-fasta) and aligned with MAFFT version 7 (*70*). Gene trees corresponding to each window were inferred using IQ-TREE version 2.1.2 on the basis of the best-fit model according to the built-in ModelFinder. A summary tree was computed from these gene trees using ASTRAL version 5.15.5 (*71*). Branch support values for gene trees were obtained by 1000 ultrafast bootstrap replicates.

### Mitochondrial tree

To investigate the phylogeny of the hamlet mitochondrial genome, we called the genotypes mapping to reference LG M, the mitochondrial genome identified in (*25*), from the GVCF above (see the “Genotyping” section). Only hamlet samples were considered, and all callable sites were taken into account except indels (16,998 bp in total). The resulting VCF files were converted to near-continuous sequences in Fasta format as above. Sequences were checked for equal length to ensure alignment and then subjected to a combined maximum likelihood tree search and nonparametric bootstrap analysis in RAxML-NG version 1.0.3 (*72*). Settings included the GTR + G substitution model, 20 each of random and parsimony starting trees, and 200 bootstrap replicates.

### Admixture analysis

Admixture between the large and small clades was first assessed with ADMIXTURE version 1.3.0 using a version of the phyps dataset without missing data, filtered by genetic linkage (see above), and converted to BED format with PLINK version 1.90b. The number of clusters *k* was set to 2, and the clustering proceeded unsupervised, i.e., without assigning the samples to the two clades a priori. Model fit was evaluated by comparing cross-validation errors. The analysis was then repeated with *k* set from 1 to 12 to test whether genotypic clusters exist within the large or small clade that may correspond to species or populations.

### Nucleotide diversity (π) and genealogical nearest neighbors

We used an ancestral recombination graph–based approach to estimate nucleotide diversity and genealogical nearest-neighbor proportions (*41*). The latter quantifies the amount of shared ancestry among individuals with respect to a priori defined groups using local topologies. For this, we relied on the unfiltered and phased version of the phylo-snp dataset. Ancestral alleles were identified with est-sfs version 2.04 (*73*) on the basis of VCFtools-derived raw allele counts and the allele identities in all three outgroup species. Sites with missing data in any samples were removed with VCFtools, the data were converted from VCF to tsinfer sample file format in Python version 3.11.4, and tree sequences were inferred with tsinfer version 0.3.1 (*41*). Nei and Li’s nucleotide diversity (*74*) was then calculated for each LG (putative chromosomes) with the tskit library (version 0.5.5) and averaged across LGs. Genealogical nearest-neighbor proportions were computed with respect to clade identity (small and large), also using tskit (*41*), and taken as averages across LG02 and both haplotypes.

### History of effective population size (*N*_e_) and divergence

The demographic history of effective population size and divergence among species was inferred with MSMC2 (*39*). This analysis was executed through a Nextflow version 20.10.0 pipeline and based on the phased version of the SNP dataset (phylo-snp). After removing the outgroup species, the density of heterozygous sites was extracted for each individual, taking into account individual sequencing depth and mapping quality to the *H. puella* reference genome assembly [masking files were obtained from deduplicated BAM files and with the help of a calling script adapted from (*26*)]. For effective population size (*N*_e_), individuals were randomly placed into groups of three or four samples from the same species and location using a custom R script (without replacement; *H. castroaguirrei* was excluded because only two samples were available). For species divergence, the analysis was restricted to the Gulf of Mexico where species from the two clades co-occur. For each pair of species, two individuals were randomly chosen per species (with replacement between comparisons). In both cases, MSMC input files were created for each group, and MSMC run with a time segmentation pattern of 1 × 2 + 25 × 1 + 1 × 2 + 1 × 3 and the average of Watterson’s estimator across input datasets (θ = 2.55 × 10^−3^). In the case of population divergence, within- and between-species coalescence rates were estimated separately and then combined into a single output file. Times and rates were interpreted assuming a mutation rate of μ = 3.7 × 10^−8^ [estimated in the three-spine stickleback in (*75*)].

### *D*-statistics

We used Patterson’s *D* and the *f*4-ratio statistic (*42*) to quantify gene flow among species/locations. Linked sites were first removed with the correlation coefficient threshold described above from a version of the phylo-snp dataset containing no missing data (936,095 sites). *D* was then calculated with Dtrios in the Dsuite package (*76*) from all 67,525 possible trios, with the *Serranus* samples serving as the outgroup. Both BBAA and Dmin topologies were considered, and *P* values were corrected for multiple testing using Bonferroni’s method. For each pair of populations, the highest *D*- and *f*4-values as well as associated *P* values were retrieved from all trios with these populations in the P2 and P3 positions.

### Demographic inference with linked selection

For this analysis, 935 noncoding, unlinked windows were selected out of the 2000 windows of the 5-kb near-continuous genomic sequence described above. This was accomplished by removing windows that overlapped with the coding sequence of gene models identified in the reference genome by MAKER (*25*). Two pseudo-haplotypes were generated for each window with VCFtools, SAMtools, and BCFtools without relying on phase information. All windows with both haplotypes were then concatenated and used as input for the Demographic Inferences with Linked Selection software version 1.0 [DILS (*43*)]. The analysis focused on the history of divergence between the small and large clades identified by the phylogenetic analyses. DILS was run with default parameters considering a per-site mutation rate μ of 3.7 × 10^−8^ following (*75*). Migration versus isolation models were compared first. Next, submodels of the best model (here, migration) were compared, including secondary contact (the daughter populations evolve initially in isolation and exchange alleles upon secondary contact) and isolation with migration (the two daughter populations continuously exchange alleles). Then, models with homogeneous (*N*_homo_) versus heterogeneous (*N*_hetero_) effective population size and, lastly, with homogeneous (*M*_homo_) versus heterogeneous (*M*_hetero_) migration were compared. The posterior probability corresponded to 1 − error rate, which is based on 140,000 simulations.

### Biogeographic analysis

We estimated ancestral ranges for the hamlet species using the R package BioGeoBEARS (*77*), considering three regions: Gulf of Mexico, Western Caribbean, and Eastern Caribbean (Fig. 2B). The author of this program recommends the following: (i) Operational taxonomic units (OTUs) should ideally be phylogenetic lineages (i.e., genetically isolated populations), and (ii) an ultrametric tree should be used as input. To satisfy the first requirement, we used two alternative pruning schemes: first, by keeping only one individual per monophyletic species/region (Scheme 1 in fig. S8, 169 OTUs) and, second, by keeping only one individual per monophyletic region (Scheme 2 in fig. S8, 58 OTUs). To meet the second requirement, we estimated relative ages for the ancestral nodes in the SNP-based whole-genome tree (Fig. 2A) using the least-square method implemented in IQ-TREE [LSD2; (*78*)]. We built a presence/absence matrix by coding each individual on the basis of the region where they were collected and evaluated six biogeographic models including DEC (*79*), DIVA (*80*), and BAYAREA (*81*), with and without the jump-dispersal or founder-speciation event (*j*) (*82*). We then assessed their fit using Akaike weights as estimated with Akaike information criterion scores corrected for small sample size (AICc). As all three models incorporating the *j* parameter demonstrate equivalent best fits (AICc weights, 0.33) for both schemes (table S5), we provide results for six competing analyses (three models per scheme).

### GWAS for species identity

Association between gene variants and species identity was examined on the basis of the phyps-snp dataset using a linear mixed model with GEMMA (*83*). This approach takes population structure into account by considering a matrix of relatedness among individuals. To remove the effect of the major phylogenetic split, this GWAS was first conducted on the large clade only, which contains 15 of the 18 named species. The dataset was transformed to PLINK format using VCFtools and PLINK. G × P association was calculated on a per-SNP basis for species identity, coded from 1 to 17 and corresponding to the 15 named hamlet species and 2 undescribed species from the large clade. The association between genotype and species identity was tested with a Wald test for each SNP with GEMMA. The results were averaged over 50-kb windows (note that Wald test *P* values were −log_10_ transformed before averaging, so −log_10_(*P*) is reported for every window). The analysis was then repeated with all species and identified the same genomic regions of high association.

### Region-specific trees

Nuclear genomic sequences characterized by a significant association with species identity (50-kb window average –log(*P* value) > 1.5) were extracted from the phylo-all dataset using VCFtools. Files were converted to Fasta format as above, and a combined maximum likelihood tree search and nonparametric bootstrap analysis was conducted using RAxML-NG version 1.0.3 (*72*). Settings included the GTR + G substitution model, 20 each of random and parsimony starting trees, and 100 bootstrap replicates. This analysis was repeated with the eight high-association regions concatenated.

### Genome assembly

Following sequencing, HiFi reads with a minimum quality score of *Q* = 20 were extracted using PacBio SMRT Link. HiFi reads were then assembled with Hifiasm version 0.19.5 (*84*) into primary and haplotype assemblies. The initial assemblies (version 1) were screened for contamination using the following approach: For each contig, the putative source organism was identified with BLAST+ (*85*) according to the National Center for Biotechnology Information’s (NCBI’s) nucleotide database downloaded on 4 April 2022. The mean coverage per contig was determined by mapping HiFi reads to the contigs with minimap version 2.22 (*86*) and SAMtools version 1.9 (*87*). Results were visualized using BlobToolKit version 3.3.4 (*88*), and contigs identified as anything other than “Chordata” or “no hit” were removed from the assemblies. The filtered primary assemblies were anchored with the help of two RAD-tag based *H. nigricans* recombination maps (*25*) using ALLMAPS (*89*), creating version 2 of the assemblies.

### RNA read filtering, alignment, and counting

Demultiplexed and converted Fastq files were quality-checked with FastQC version 0.11.5 (table S6) (*90*). Illumina adapters were removed and low-quality reads were trimmed with Trimmomatic version 0.36 (*91*) using a sliding-window procedure. Filtered reads were aligned to the *H. puella* reference genome (*25*) using HISAT2 version 2.1.0 (*92*). HTSeq version 0.13.5 (*93*) was used to quantify the number of reads unambiguously mapped per gene, and read counts were filtered and normalized using the standard DESeq2 version 1.42 analysis pipeline (*94*). A sample clustering analysis on regularized log-transformed expression values identified two outlier samples (one *H. puella* retina and one *H. nigricans* brain), which were removed from subsequent analyses.

### Coexpression network analysis

Coexpression networks were constructed for each tissue with WGCNA (*95*). Genes with low expression counts were filtered and variance-stabilizing transformation was carried out with DESEq2 (*94*). A total of 22,046, 18,249, and 18,423 genes remained after filtering in the brain, retina, and skin tissue, respectively. A soft thresholding power was chosen for each tissue (four for brain, three for retina, and six for skin) on the basis of the criterion of approximate scale-free topology (*96*). On the basis of this power, a weighted correlation network was constructed for each tissue. Modules, i.e., clusters of genes whose expression correlates highly with each other, were identified, as well as the genes belonging to the same module as *casz1*. We then identified over-represented biological processes, molecular functions, and cellular components of genes coexpressed with *casz1* in at least two tissues with GOstats version 2.68 (*97*) and GSEABase version 1.64 (*98*). To correct for multiple testing, we used the false discovery rate method implemented in goEnrichment version 1.0 (*99*).

### *casz1* splicing analysis

Considering that the strongest association signal within the *casz1* transcription factor gene was located in the region between the first and fifth exons, which harbors large introns, we tested for differential splicing of *casz1* among species (*H. puella*, *H. nigricans*, and *H. unicolor*) across the skin, brain, and retina tissues. Exon-specific reads were counted and tested for differential exon usage with DEXseq version 1.48 (*100*).

### Annotation and analysis of transposable elements

Repeat libraries were generated de novo for the *H. puella* reference genome. This was achieved with RepeatModeler version 2.0 (*101*), relying on the LTRharvest (*102*) and LTR_retriever (*103*) options to enhance LTR (long terminal repeat) detection. The libraries were then used to annotate the genome with RepeatMasker version 4.0.1 (*104*). The resulting repeat annotation and a curated version of the reference gene annotation were used to build a genome index and map our RNA sequencing reads with STAR version 2.7.10 (*105*). Reads were then counted, normalized, and tested for differential expression in transposable elements among species with TEtranscripts version 2.2.1 (*106*).

### Structural variation in the *casz1* region

We extracted the *casz1* region from the *H. puella* reference genome, from the start of the association region (LG12 position 20,135,000) until the end of the *casz1* gene (LG12 position 20,347,811). We then retrieved this same region from our six PacBio haplotype assemblies using the megablast function in BLAST+ version 2.14.1 (*85*) and SAMtools version 1.18 faidx (*87*). Last, pairwise alignments were constructed using Minimap2 version 2.26 (*86*) and checked for structural rearrangements with SyRI version 1.6.3 (*107*). The results were visualized with plotsr version 1.1.1 (*108*).

### Ethics statement

This study builds on a sampling effort conducted over 18 years (2004 to 2022) at 15 locations across the Greater Caribbean region. It was developed and conducted in collaboration with local institutions and scientists, following specific Institutional Animal Care and Use Committee protocols (notably STRI 2013-0301-2016, 2017-0101-2020-2, SI-21007, NU 17-0206, and UPR 01009-02-16-2015). It includes coauthors who are or were permanently based in Mexico, Panama, Colombia, and Puerto Rico.
