# Supplementary material for: Radiation with reproductive isolation in the near-absence of phylogenetic signal
Source: Sci Adv. 2025 Jul 25;11(30):eadt0973. doi: 10.1126/sciadv.adt0973 (PMC12292942; doi:10.1126/sciadv.adt0973)
Supplement: Supplementary file 1 — Figs. S1 to S27 Tables S1 to S6 [file sciadv.adt0973_sm.pdf]

Science Advances
AAAS

Supplementary Materials for

Radiation with reproductive isolation in the near-absence of phylogenetic signal

Martin Helmkampf *et al.*

Corresponding author: Martin Helmkampf, martin.helmkampf@leibniz-zmt.de

Sci. Adv. **11**, eadt0973 (2025)
DOI: 10.1126/sciadv.adt0973

This PDF file includes:

Figs. S1 to S27
Tables S1 to S6

Supplementary Figures

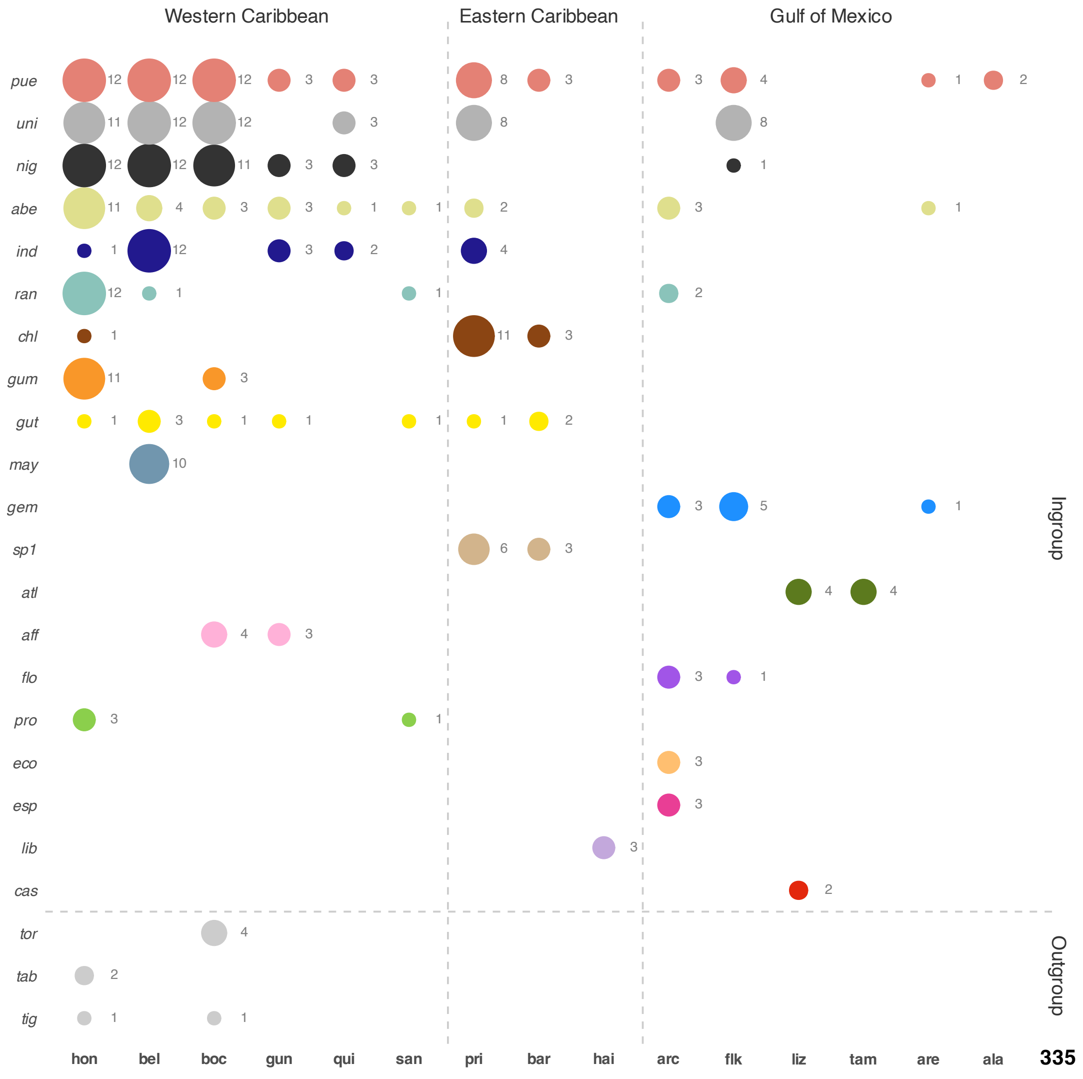

Fig. S1. Overview of the sampling design, comprising 327 samples of all described hamlet species. pue: *Hypoplectrus puella*, uni: *Hypoplectrus unicolor*, nig: *Hypoplectrus nigricans*, abe: *Hypoplectrus aberrans*, ind: *Hypoplectrus indigo*, ran: *Hypoplectrus randallorum*, chl: *Hypoplectrus chlorurus*, gum: *Hypoplectrus gummigutta*, gut: *Hypoplectrus guttavarius*, may: *Hypoplectrus maya*, gem: *Hypoplectrus gemma*, sp1: *Hypoplectrus sp. 1*, atl: *Hypoplectrus atlahua*, aff: *Hypoplectrus affinis*, flo: *Hypoplectrus floridae*, pro: *Hypoplectrus providencianus*, eco: *Hypoplectrus ecosur*, esp: *Hypoplectrus espinosai*, lib: *Hypoplectrus liberte*, cas: *Hypoplectrus castroaguirrei*. In addition, three outgroup species were included – tor: *Serranus tortugarum*, tab: *Serranus tabacarius* and tig: *Serranus tigrinus*. Samples were collected at 15 locations covering the Gulf of Mexico [Tamiahua (tam), Antón Lizardo (liz), Cayos Arcas (arc), Cayo Arenas (are), Alacranes Reef (ala) and the Florida Keys (flk)], the Western Caribbean [Quintana Roo (qui), Belize (bel), Honduras (hon), San Andrés (san), Bocas del Toro (boc) and Guna Yala (gun)], and the Eastern Caribbean [Haiti (hai), Puerto Rico (pri) and Barbados (bar)].

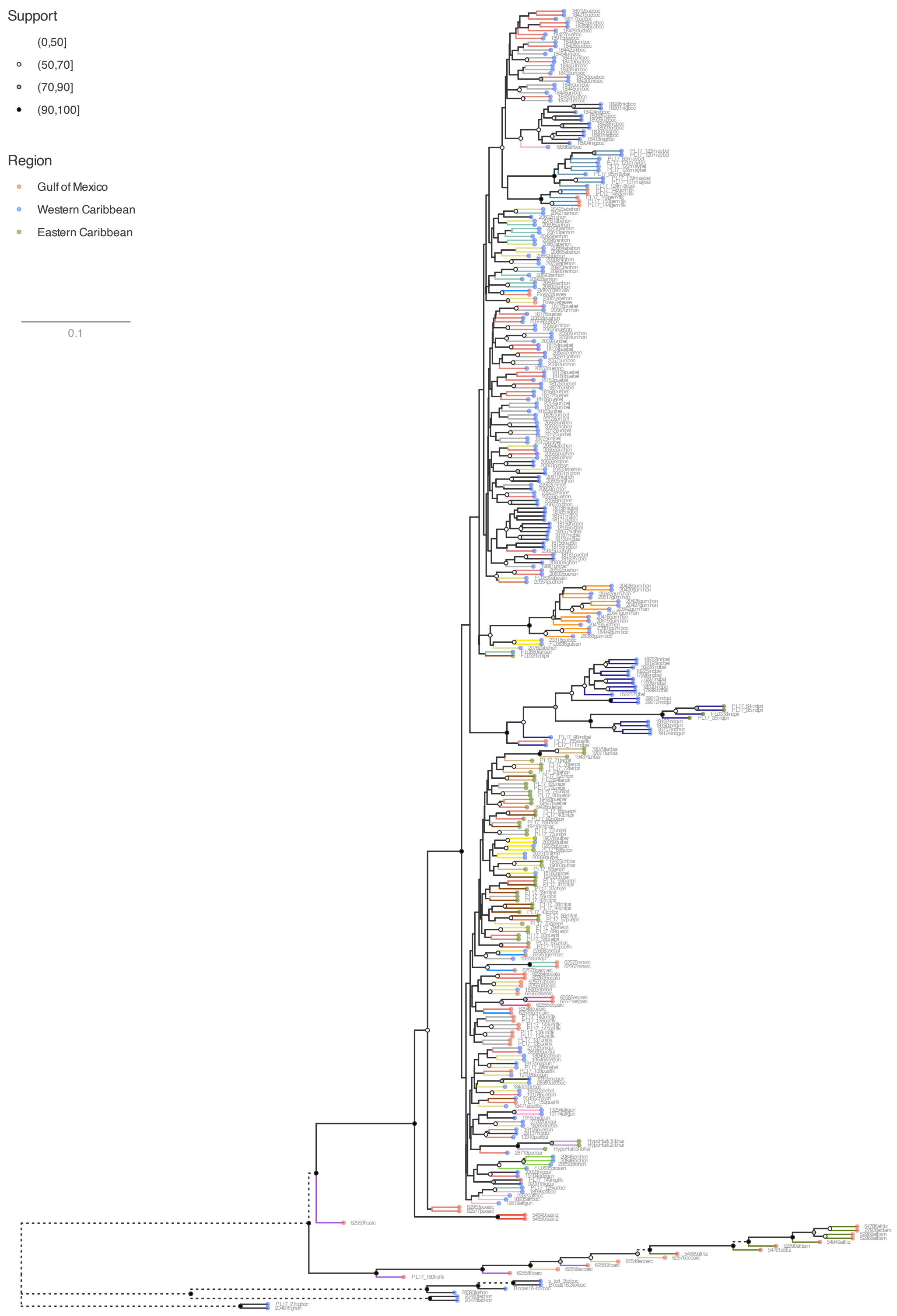
Support
(0,50]
(50,70]
(70,90]
(90,100]
Region
Gulf of Mexico
Western Caribbean
Eastern Caribbean
0.1

Fig. S2. Summary tree inferred with ASTRAL-III from 2000 local trees. Each local tree was based on a randomly chosen 5 kb genomic window. Local trees were reconstructed using IQ-TREE v2, the best-fit substitution model according to ModelFinder, and 1000 ultrafast bootstrap replicates. Internal branches reflect concordance among local trees (measured in coalescent units; terminal branches were set to an arbitrary constant). Dashed branches are displayed at 5 % of their original value to improve visualization. Values at the nodes denote quartet (as opposed to bipartition) support, expressed in local posterior probabilities. Branch colors indicate species as defined in Fig. 1 and fig. S1. The last six letters of the sample names refer to the species (first three letters) and location (last three letters) as in fig. S1.

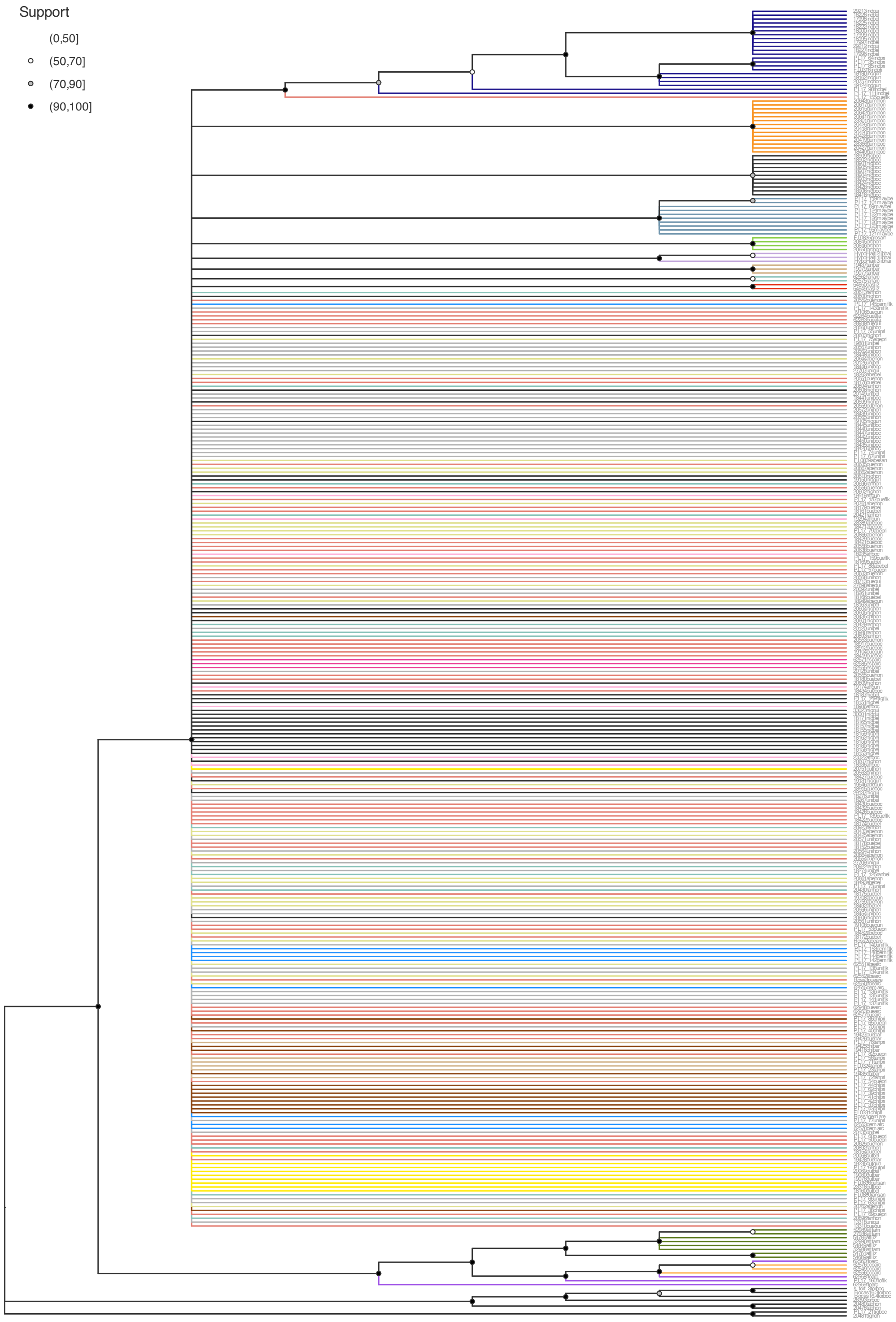
Support
(0,50]
(50,70]
(70,90]
(90,100]

Fig. S3. SVDQuartets 50 % majority consensus tree based on approximately 110 k SNPs. The consensus was derived from 200 bootstrap replicates each considering 5 million quartets (1 % of all possible quartets). Branch colors indicate species as defined in Fig. 1 and fig. S1. The last six letters of the sample names refer to the species (first three letters) and location (last three letters) as in fig. S1.

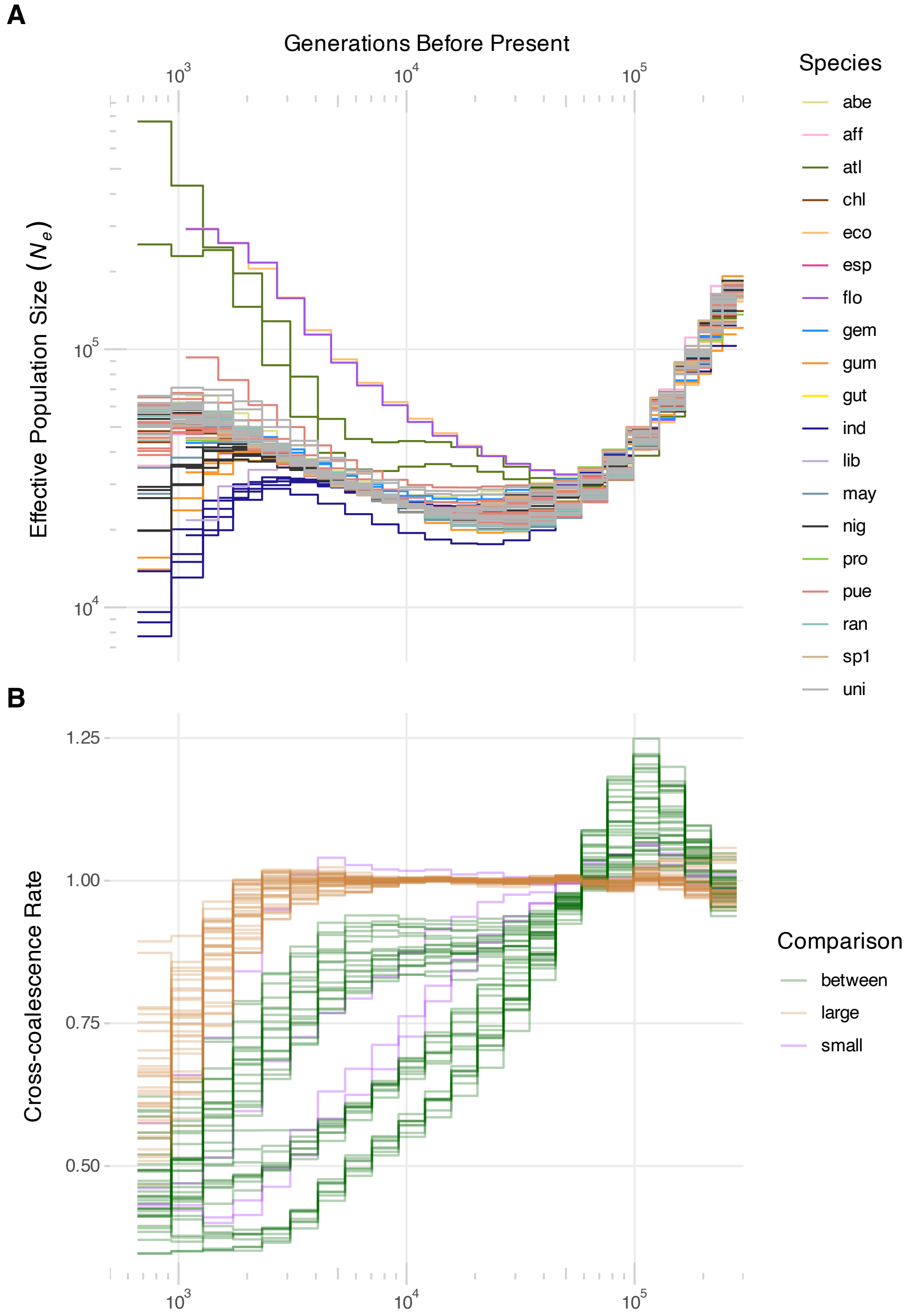
A
Generations Before Present
10^3
10^4
10^5
Effective Population Size (N_e)
10^5
10^4
Species
abe
aff
atl
chl
eco
esp
flo
gem
gum
gut
ind
lib
may
nig
pro
pue
ran
sp1
uni
B
Cross-coalescence Rate
1.25
1.00
0.75
0.50
10^3
10^4
10^5
Comparison
between
large
small

Fig. S4. Effective population size and divergence history as inferred by MSMC2. (**A**) Effective population size over time, with each line representing a set of three or four genomes of the same species (randomly chosen without replacement) *H. castroaguirrei* was excluded from this analysis because only two genomes were available for this species. (**B**) Cross-coalescence rates between pairs of species from the Gulf of Mexico, where species from the two clades co-occur. Each line represents an independent comparison of two genomes each from two species of the same location within the Gulf of Mexico. Comparisons between small and large clade species are shown in green, within large clade comparisons in orange, and within small clade comparisons in purple. Rates of one and zero indicate completely shared and no shared ancestry, respectively. Time in generations was based on a per-site mutation rate of $\mu = 3.7 \times 10^{-8}$. The four most recent and most ancient time segments were considered unreliable and excluded from plotting. The results suggest that the small and large clades separated between 50,000 and 70,000 generations ago (corresponding to 50,000–210,000 years on the basis of an estimated generation time of 1–3 years).

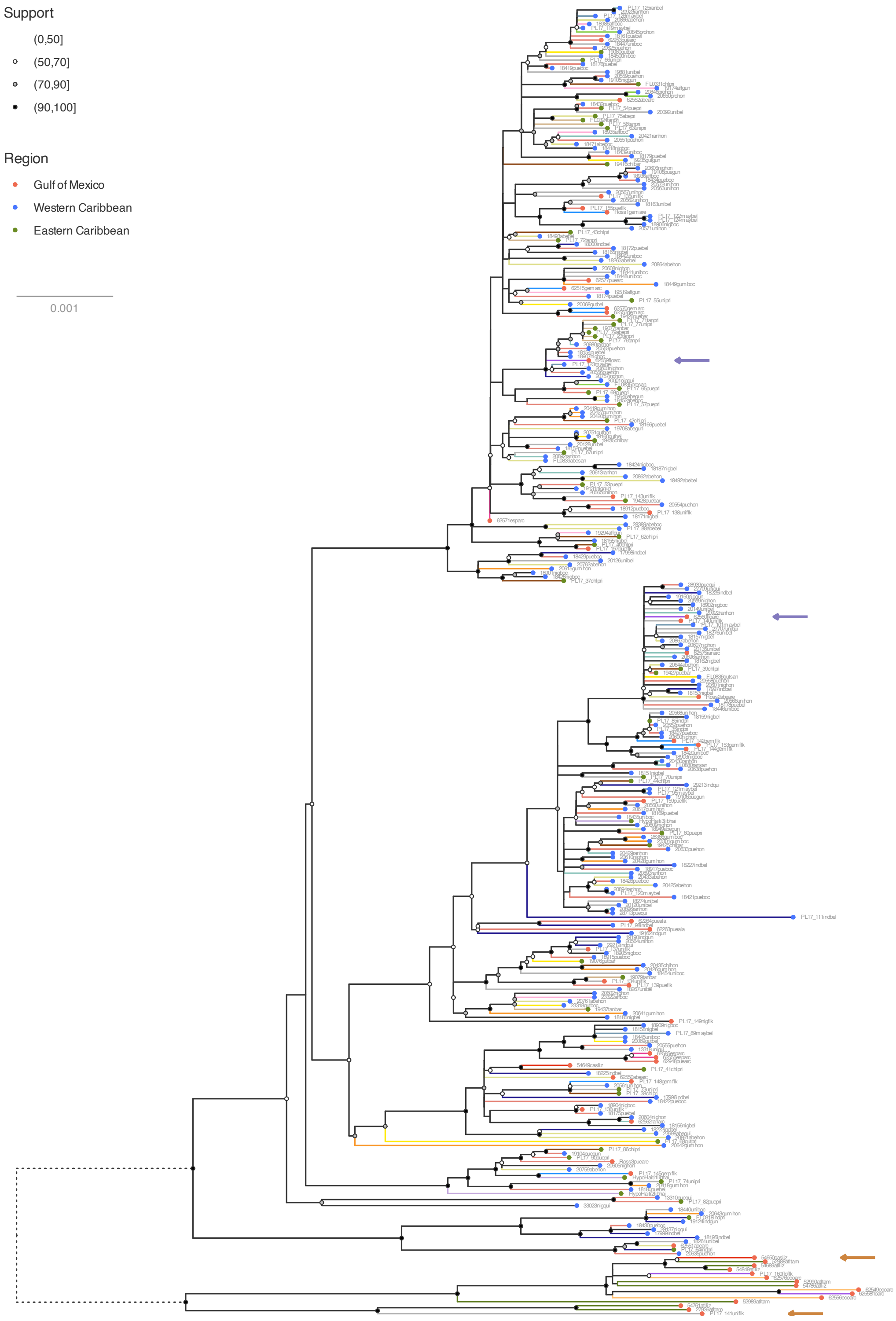

Support
(0,50]
(50,70]
(70,90]
(90,100]
Region
Gulf of Mexico
Western Caribbean
Eastern Caribbean
0.001

Fig. S5. Mitochondrial genome phylogeny generated with RAxML-NG. The analysis is based on the hamlet-only dataset including all mitochondrial sites (approximately 17 kb), the GTR+G model and thorough search parameters. Support values at the nodes were obtained from 200 nonparametric bootstrap replicates. Dashed branches are displayed at 10 % of their original value to improve visualization. Branch colors indicate species as defined in Fig. 1 and fig. S1. The last six letters of the sample names refer to the species (first three letters) and location (last three letters) as in fig. S1. Colored arrows highlight individuals with mitonuclear discordances (nuclear genome from the large clade and mitochondrial genome from the small clade, in orange, and vice versa, in purple).

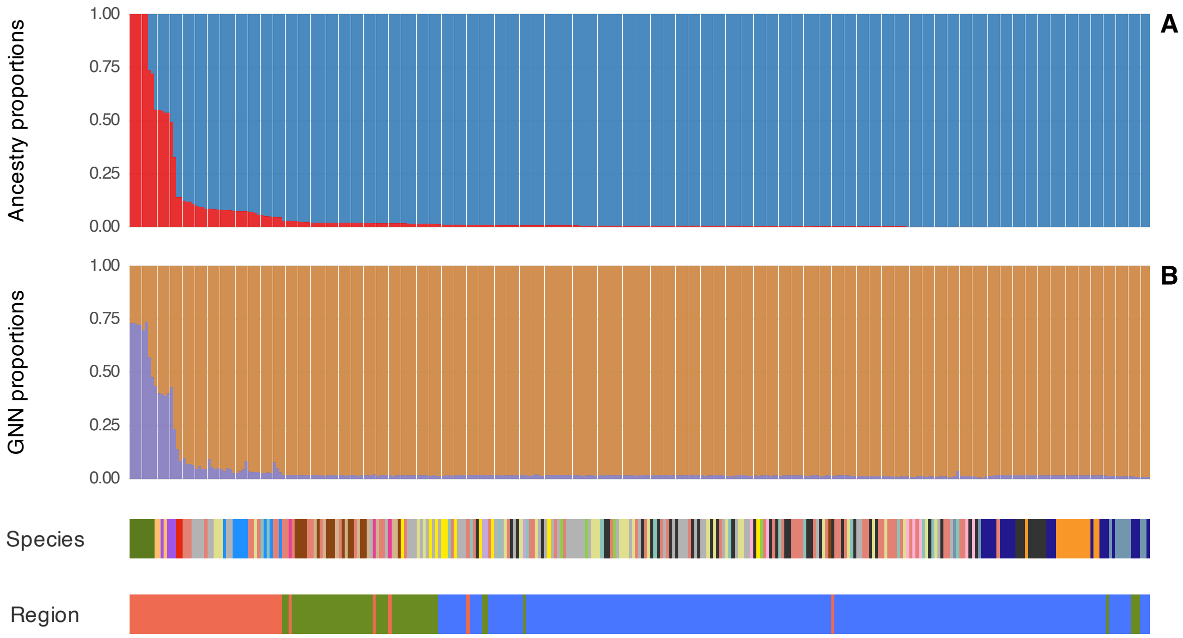
Ancestry proportions
1.00
0.75
0.50
0.25
0.00
A
GNN proportions
1.00
0.75
0.50
0.25
0.00
B
Species
Region

Fig. S6. Admixture between the large and small clades. (**A**) Ancestry proportion plot obtained with ADMIXTURE for *k* (number of ancestral components) = 2, based on a version of the genome-wide SNP dataset (*phyps-snp*). Each individual is represented by a vertical bar broken up into two colored segments whose lengths are proportional to the contributions of the two ancestral components to the genome of the individual. The two ancestral components match the two clades identified by the phylogeny, with evidence of admixture between the two. (**B**) Average genealogical nearest neighbors (GNN) proportions for all individuals. Purple and orange bar segments indicate GNN proportions with respect to the small and large clade, respectively. This analysis is based on both haplotypes of putative chromosome LG02 and was performed with tskit. Individuals are arranged in the same order as in the admixture plot above. Assuming that LG02 is representative of the whole genome, the plot reflect the amount of shared ancestry among individual haplotypes with respect to the two clades. Here again there is evidence of admixture between the two clades. The colored bars below the plots indicate species and region following Fig. 1 and 2, and fig. S1.

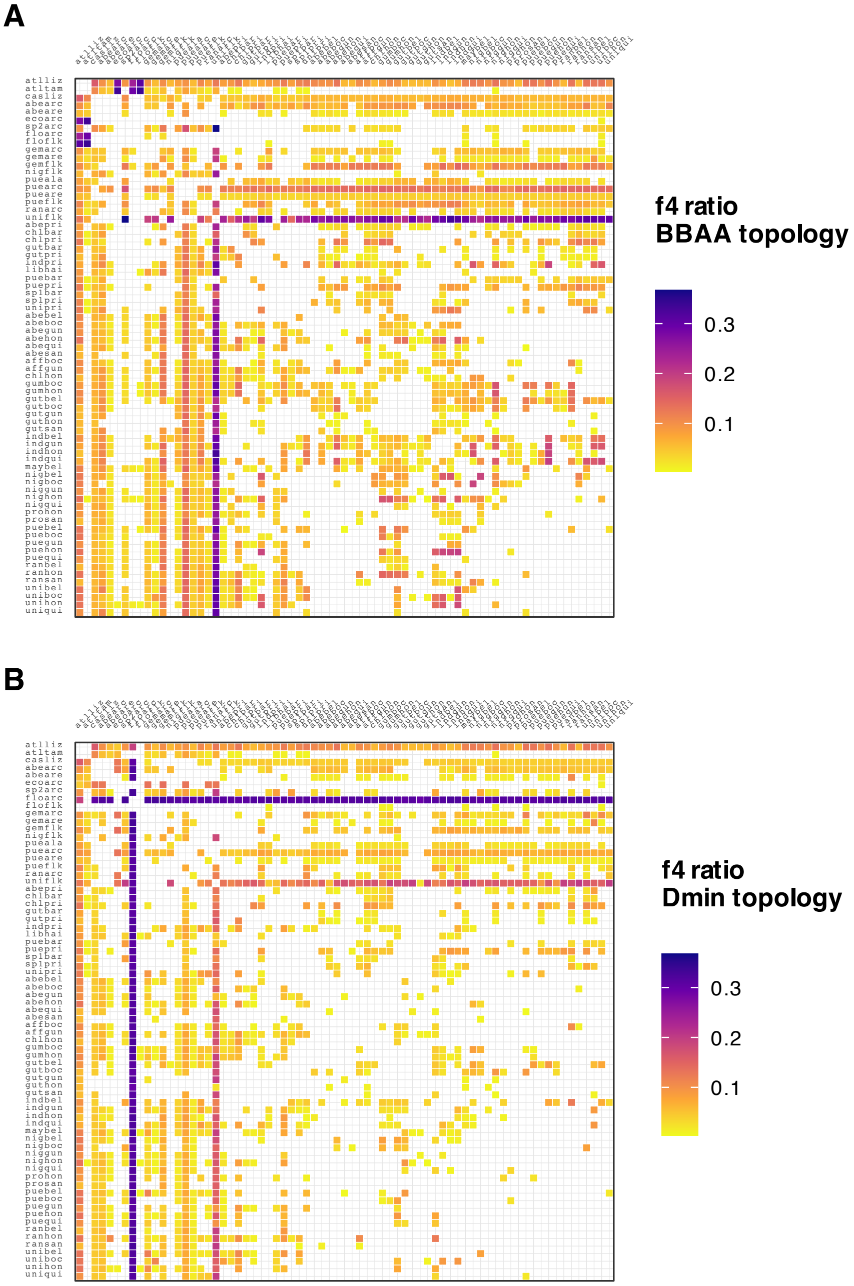
A
f4 ratio
BBAA topology
0.3
0.2
0.1
B
f4 ratio
Dmin topology
0.3
0.2
0.1

Fig. S7. Introgression between the two clades. f_4-ratios calculated by Dtrios (Dsuite package) between all population pairs, considering (**A**) BBAA and (**B**) Dmin topologies. The x- and y-axes represent positions P2 and P3, respectively. For each pair, only the trio with the lowest Bonferroni-corrected p-value regarding D is considered (same-pair trios differ at the P1 position). Tile colors indicate f_4-ratios corresponding to D-values that were significant at the 0.05 level, with white tiles representing pairs without significant D-values. The analysis was based on 936 k SNPs (*phyps-snp* dataset with linked sites removed and containing no missing data), and the *Serranus* samples serving as outgroup. The f_4-ratio estimates the proportion of introgressed DNA in admixed populations. Thus, gene flow between populations seems to be widespread among hamlets, including between clades, and affect substantial parts of the genome.

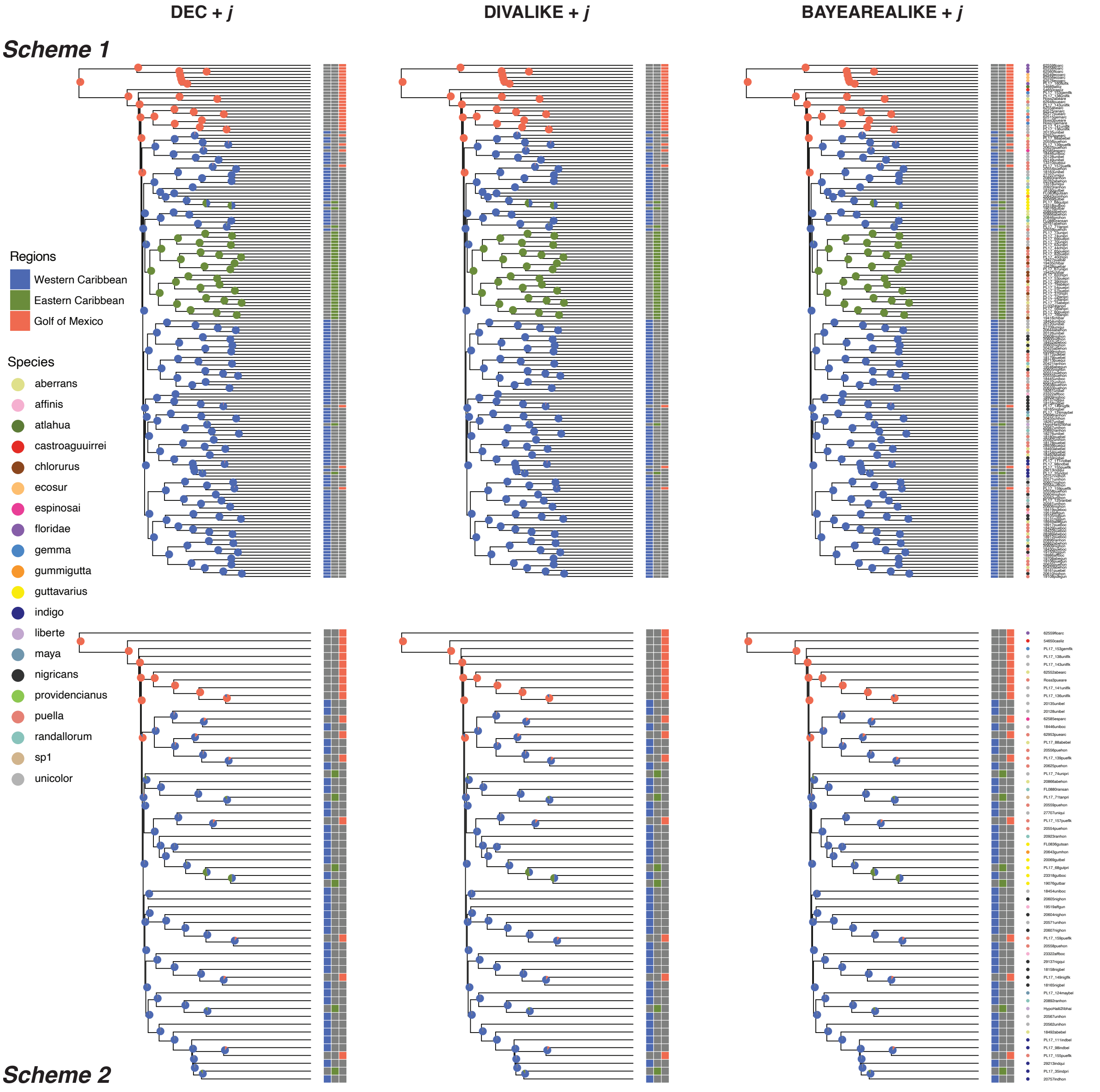
DEC + *j*
DIVALIKE + *j*
BAYEAREALIKE + *j*
Scheme 1
Scheme 2
Regions
Western Caribbean
Eastern Caribbean
Golf of Mexico
Species
aberrans
affinis
atlahua
castroaguuirrei
chlorurus
ecosur
espinosai
floridae
gemma
gummigutta
guttavarius
indigo
liberte
maya
nigricans
providencianus
puella
randallorum
sp1
unicolor

Fig. S8. Inference of ancestral ranges. This analysis was conducted with BioGeoBEARS based on the ultrametric whole-genome tree generated with IQ-TREE, pruned to retain only one individual per monophyletic species/region (Scheme 1) or only one individual per monophyletic region (Scheme 2). Results shown depict ancestral ranges according to three competing models (see table S5). Branch lengths are relative ages from 0 at the root to 1 at the tips, estimated by the least-square method implemented in IQ-TREE based on the ancestral nodes in the SNP-based whole-genome tree (Fig. 2A).

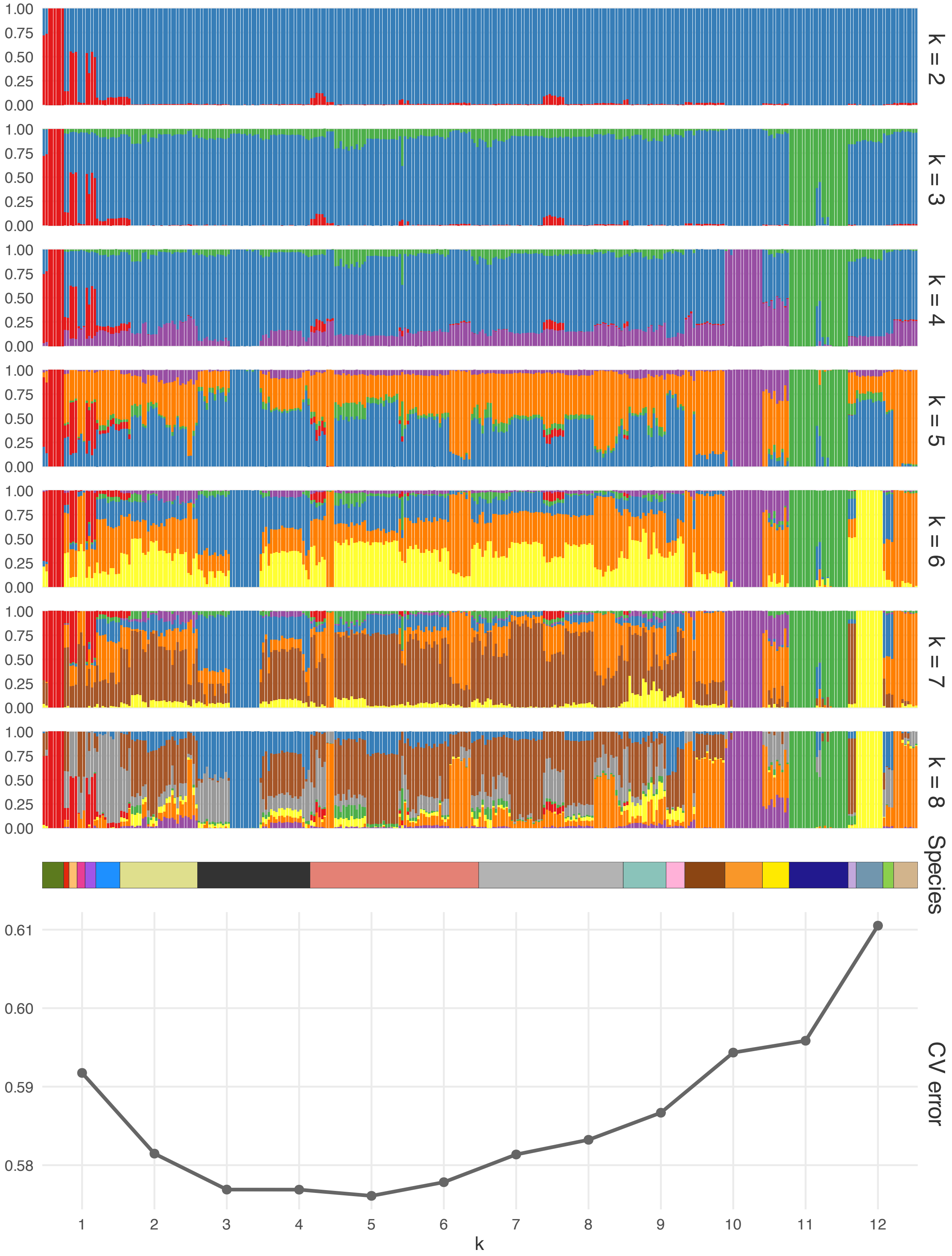

1.00
0.75
0.50
0.25
0.00
k = 2
k = 3
k = 4
k = 5
k = 6
k = 7
k = 8
Species
0.61
0.60
0.59
0.58
CV error
1 2 3 4 5 6 7 8 9 10 11 12
k

Fig. S9. Ancestry proportions. (**A**) Barplots showing estimated ancestry proportions for $k = 2$ to 8, based on ADMIXTURE analysis of a version of the genome-wide SNP dataset (*phyps-snp*). Each individual is represented by a vertical bar broken up into colored segments whose lengths are proportional to the contributions of ancestral components to the genome of the individual. k determines the number of ancestral components or genetic clusters considered, with 5 being the optimal value according to the comparison of cross-validation errors (**B**). The colored horizontal bar below the barplots indicates the species, following the color scheme defined in Figs. 1 and S1.

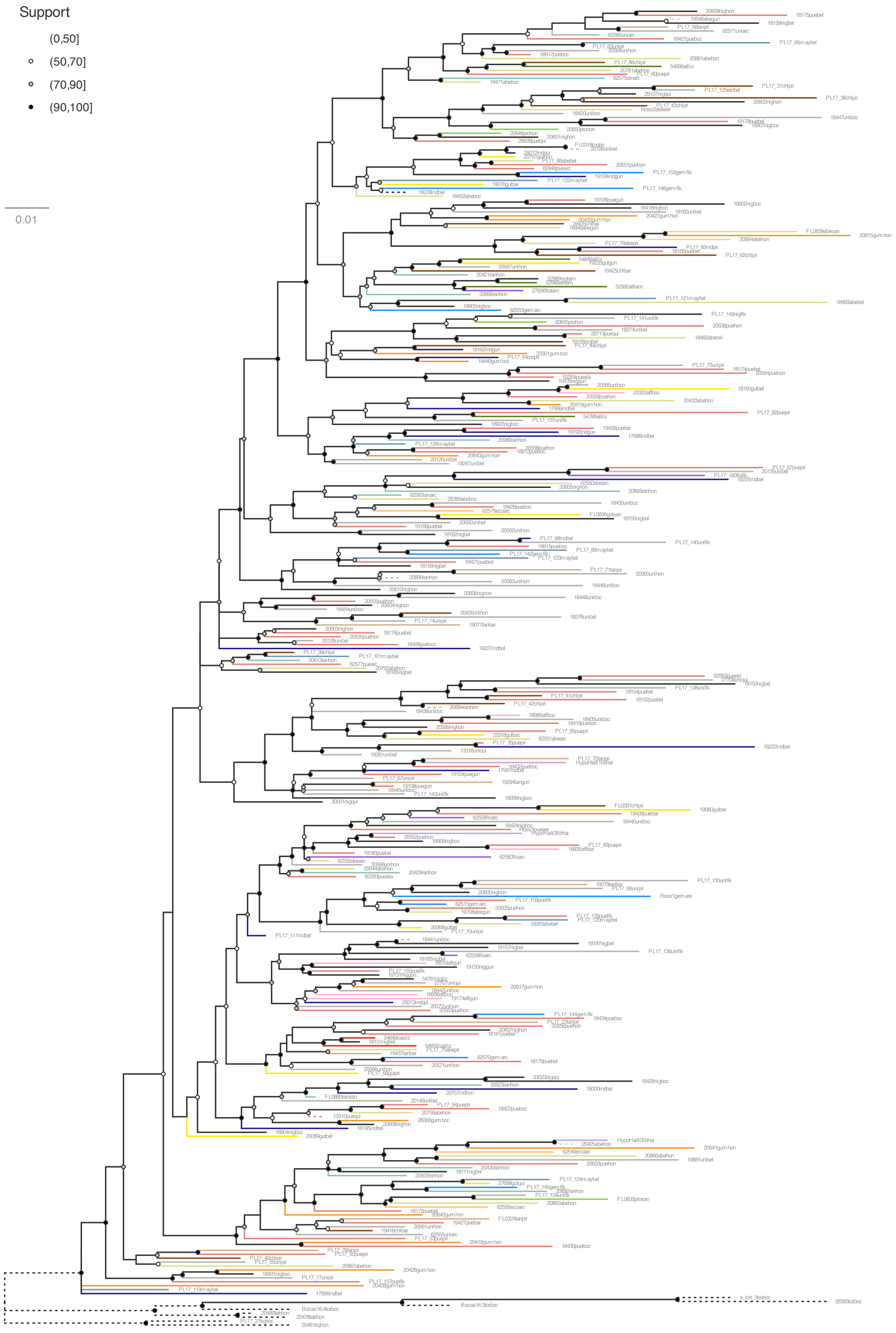
Support
(0,50]
(50,70]
(70,90]
(90,100]
0.01

Fig. S10. Local phylogeny with highest mean support. The highest mean support is calculated across nodes among the 2000 genomic windows of 5 kb length that were considered. Support values were obtained by ultrafast bootstrap approximation with 1000 replicates implemented in IQ-TREE. Dashed branches are displayed at 5 % of their original value to improve visualization. Branch colors signify species as defined in Fig. 1 and fig. S1. The last six letters of the sample names refer to the species (first three letters) and location (last three letters) as in fig. S1.

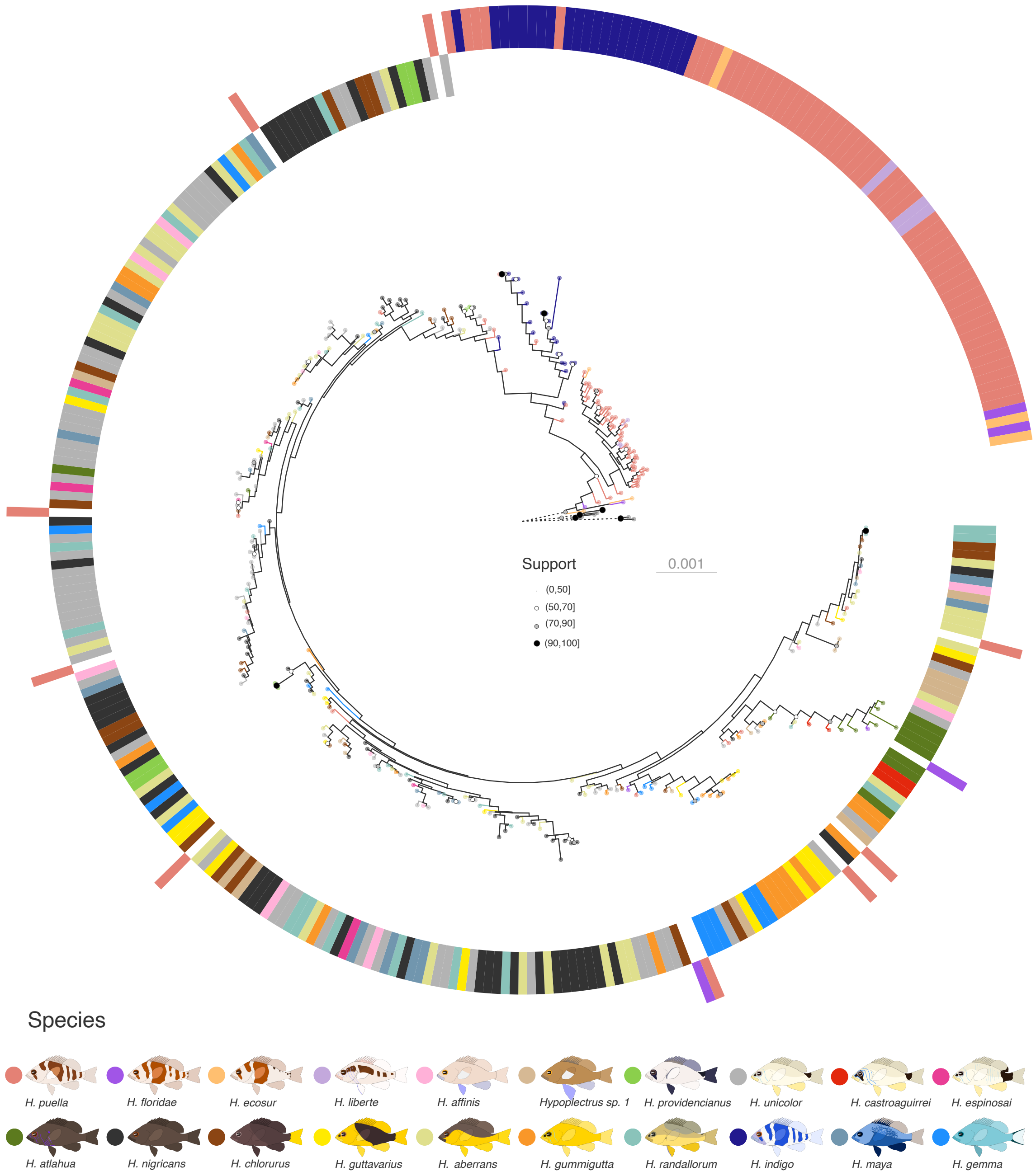
Support
0.001
(0,50]
(50,70]
(70,90]
(90,100]
Species
H. puella
H. floridae
H. ecosur
H. liberte
H. affinis
Hypoplectrus sp. 1
H. providencianus
H. unicolor
H. castroaguirrei
H. espinosai
H. atlahua
H. nigricans
H. chlorurus
H. guttavarius
H. aberrans
H. gummigutta
H. randallorum
H. indigo
H. maya
H. gemma

Fig. S11. Phylogeny of the *casz1* gene including both introns and exons, spanning > 152 kb. The rings, branch and tip point colors indicate hamlet species as shown by the icons below the tree. The outer ring corresponds to species that have a barred color pattern and the inner ring the species that have a non-barred color pattern. Node point size and color represent node support. The eight *Serranus* outgroup individuals are shown in gray and the length of their branches, represented with dashed lines, are reduced to 5 % of their actual length to improve the visualization of the hamlet radiation.

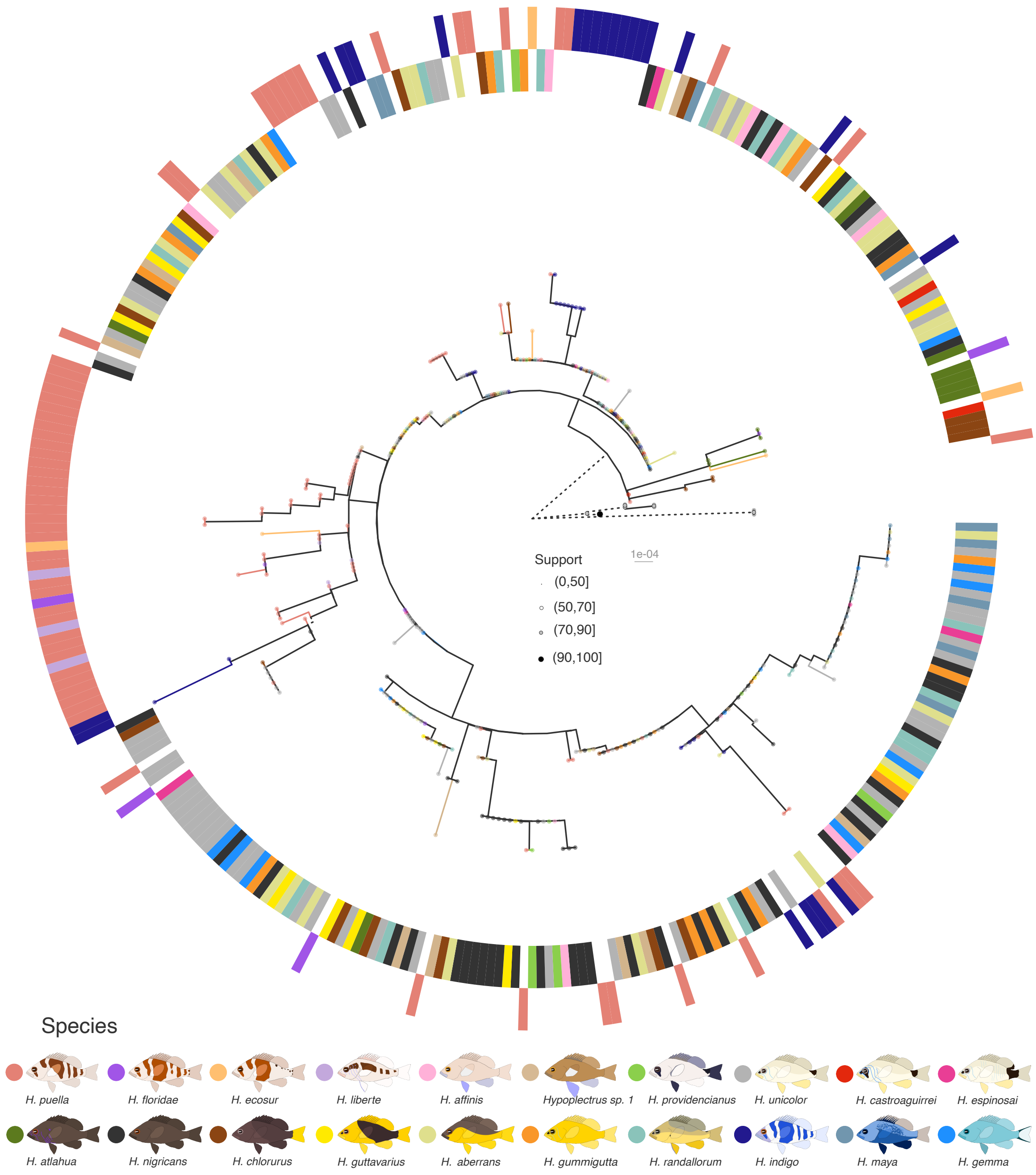
Support
(0,50]
(50,70]
(70,90]
(90,100]
1e-04
Species
H. puella
H. floridae
H. ecosur
H. liberte
H. affinis
Hypoplectrus sp. 1
H. providencianus
H. unicolor
H. castroaguirrei
H. espinosai
H. atlahua
H. nigricans
H. chlorurus
H. guttavarius
H. aberrans
H. gummigutta
H. randallorum
H. indigo
H. maya
H. gemma

Fig. S12. Phylogeny of the *casz1* gene including exons only, spanning 6689 bp. The outer ring, branch and tip point colors indicate hamlet species as shown by the icons below the tree. Node point size and color represent node support. The eight *Serranus* outgroup individuals are shown in gray and the length of their branches, represented with dashed lines, are reduced to 5 % of their actual length to improve the visualization of the hamlet radiation.

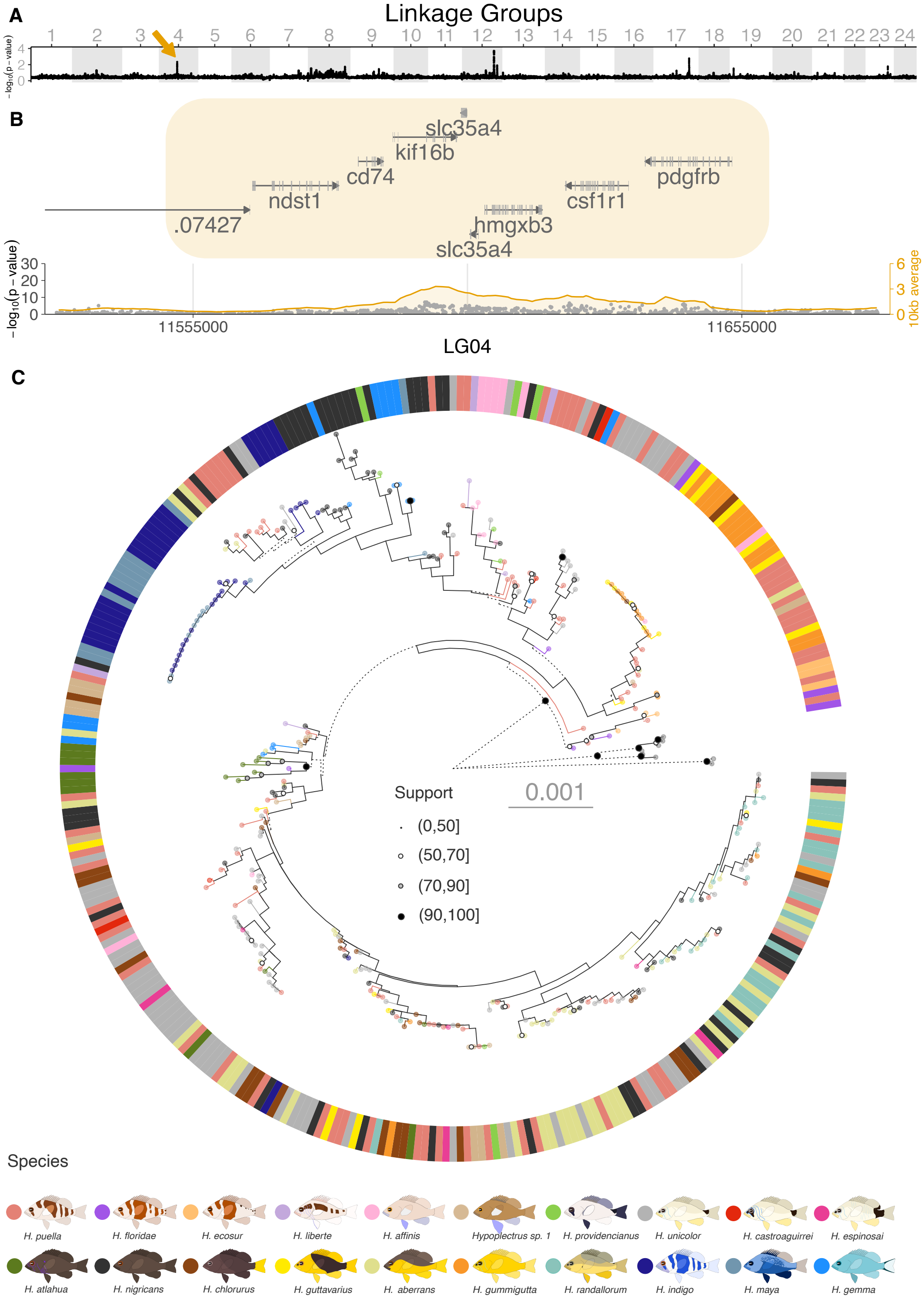

A
Linkage Groups
1 2 3 4 5 6 7 8 9 10 11 12 13 14 15 16 17 18 19 20 21 22 23 24
-log10(p-value)
B
slc35a4
kif16b
cd74
pdgfrb
ndst1
csf1r1
.07427
hmgxb3
slc35a4
−log10(p−value)
10kb average
11555000
11655000
LG04
C
Support
(0,50]
(50,70]
(70,90]
(90,100]
0.001
Species
H. puella
H. floridae
H. ecosur
H. liberte
H. affinis
Hypoplectrus sp. 1
H. providencianus
H. unicolor
H. castroaguirrei
H. espinosai
H. atlahua
H. nigricans
H. chlorurus
H. guttavarius
H. aberrans
H. gummigutta
H. randallorum
H. indigo
H. maya
H. gemma

Fig. S13. GWAS for species identity and phylogeny of the LG04 association peak. (**A**) Results of the genome-wide association study (GWAS) for species identity. The gray and white blocks represent the 24 linkage groups (LG, putative chromosomes) and the black dots are averages of the log-transformed *p*-value of the GWAS association over 50 kb windows with 5 kb increments. (**B**) Close-up on the LG04 association peak. The x-axis shows the position on LG04 (in bp) and the orange rectangle highlights the region that shows an association $>$ 1.5 considering 50 kb windows. gray dots represent the GWAS log-transformed *p* value for each individual SNP, and the orange line its average over 10 kb windows. (**C**) Phylogeny of the LG04 region, covering 91,382 bp centered on the GWAS association peak highlighted in orange in **B**. The outer ring, branch and tip point colors indicate hamlet species as shown by the icons below the tree. Node point size and color represent node support. The eight *Serranus* outgroup individuals are shown in gray and the length of their branches, represented with dashed lines, are reduced to 5 % of their actual length to improve the visualization of the hamlet radiation.

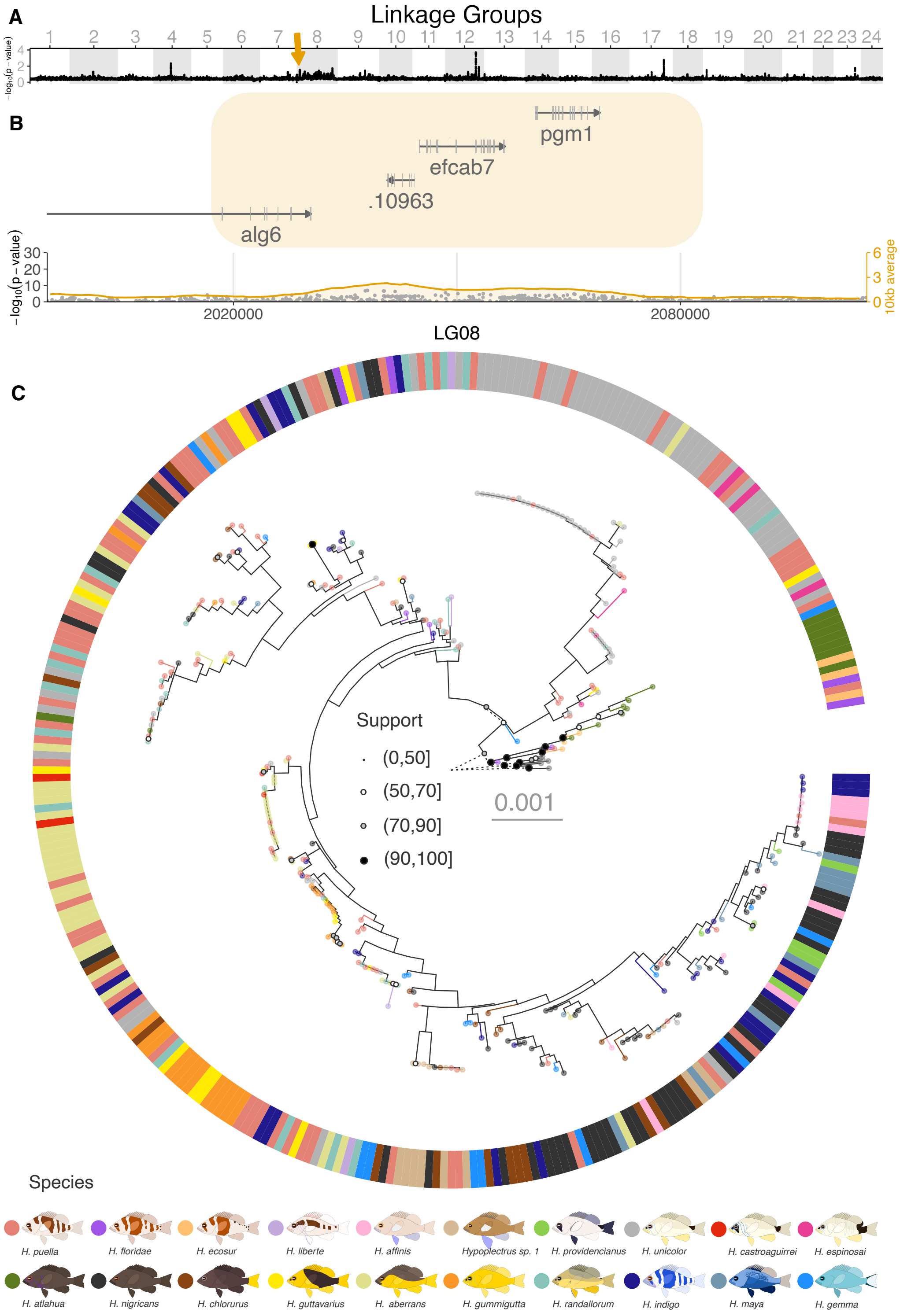

A
Linkage Groups
1 2 3 4 5 6 7 8 9 10 11 12 13 14 15 16 17 18 19 20 21 22 23 24
−log10(p − value)
B
pgm1
efcab7
.10963
alg6
−log10(p − value)
10kb average
2020000
2080000
LG08
C
Support
(0,50]
(50,70]
(70,90]
(90,100]
0.001
Species
H. puella
H. floridae
H. ecosur
H. liberte
H. affinis
Hypoplectrus sp. 1
H. providencianus
H. unicolor
H. castroaguirrei
H. espinosai
H. atlahua
H. nigricans
H. chlorurus
H. guttavarius
H. aberrans
H. gummigutta
H. randallorum
H. indigo
H. maya
H. gemma

Fig. S14. GWAS for species identity and phylogeny of the first LG08 association peak. (**A**) Results of the genome-wide association study (GWAS) for species identity. The gray and white blocks represent the 24 linkage groups (LG, putative chromosomes) and the black dots are averages of the log-transformed *p*-value of the GWAS association over 50 kb windows with 5 kb increments. (**B**) Close-up on the first LG08 association peak. The x-axis shows the position on LG08 (in bp) and the orange rectangle highlights the region that shows an association $>$ 1.5 considering 50 kb windows. gray dots represent the GWAS log-transformed *p* value for each individual SNP, and the orange line its average over 10 kb windows. (**C**) Phylogeny of the LG08 region, covering 55,355 bp centered on the GWAS association peak highlighted in orange in **B**. The outer ring, branch and tip point colors indicate hamlet species as shown by the icons below the tree. Node point size and color represent node support. The eight *Serranus* outgroup individuals are shown in gray and the length of their branches, represented with dashed lines, are reduced to 2 % of their actual length to improve the visualization of the hamlet radiation.

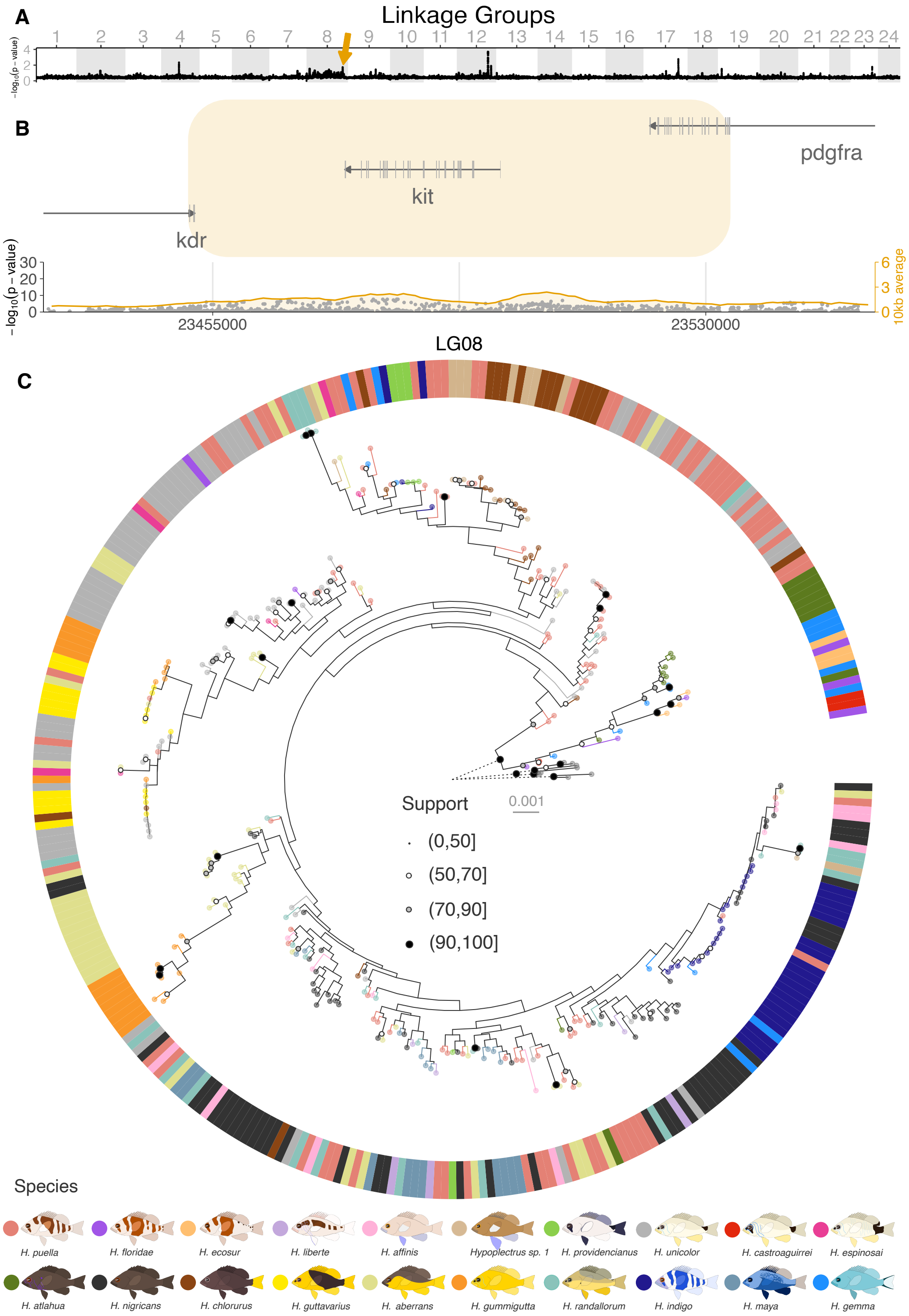

A
Linkage Groups
1 2 3 4 5 6 7 8 9 10 11 12 13 14 15 16 17 18 19 20 21 22 23 24
$-\log_{10}(p-value)$
B
pdgfra
kit
kdr
$-\log_{10}(p-value)$
10kb average
23455000
23530000
LG08
C
Support
0.001
(0,50]
(50,70]
(70,90]
(90,100]
Species
H. puella
H. floridae
H. ecosur
H. liberte
H. affinis
Hypoplectrus sp. 1
H. providencianus
H. unicolor
H. castroaguirrei
H. espinosai
H. atlahua
H. nigricans
H. chlorurus
H. guttavarius
H. aberrans
H. gummigutta
H. randallorum
H. indigo
H. maya
H. gemma

Fig. S15. GWAS for species identity and phylogeny of the second LG08 association peak. (**A**) Results of the genome-wide association study (GWAS) for species identity. The gray and white blocks represent the 24 linkage groups (LG, putative chromosomes) and the black dots are averages of the log-transformed *p*-value of the GWAS association over 50 kb windows with 5 kb increments. (**B**) Close-up on the second LG08 association peak. The x-axis shows the position on LG08 (in bp) and the orange rectangle highlights the region that shows an association $>$ 1.5 considering 50 kb windows. gray dots represent the GWAS log-transformed *p* value for each individual SNP, and the orange line its average over 10 kb windows. (**C**) Phylogeny of the second LG08 region, covering 67,783 bp centered on the GWAS association peak highlighted in orange in **B**. The outer ring, branch and tip point colors indicate hamlet species as shown by the icons below the tree. Node point size and color represent node support. The eight *Serranus* outgroup individuals are shown in gray and the length of their branches, represented with dashed lines, are reduced to 5 % of their actual length to improve the visualization of the hamlet radiation.

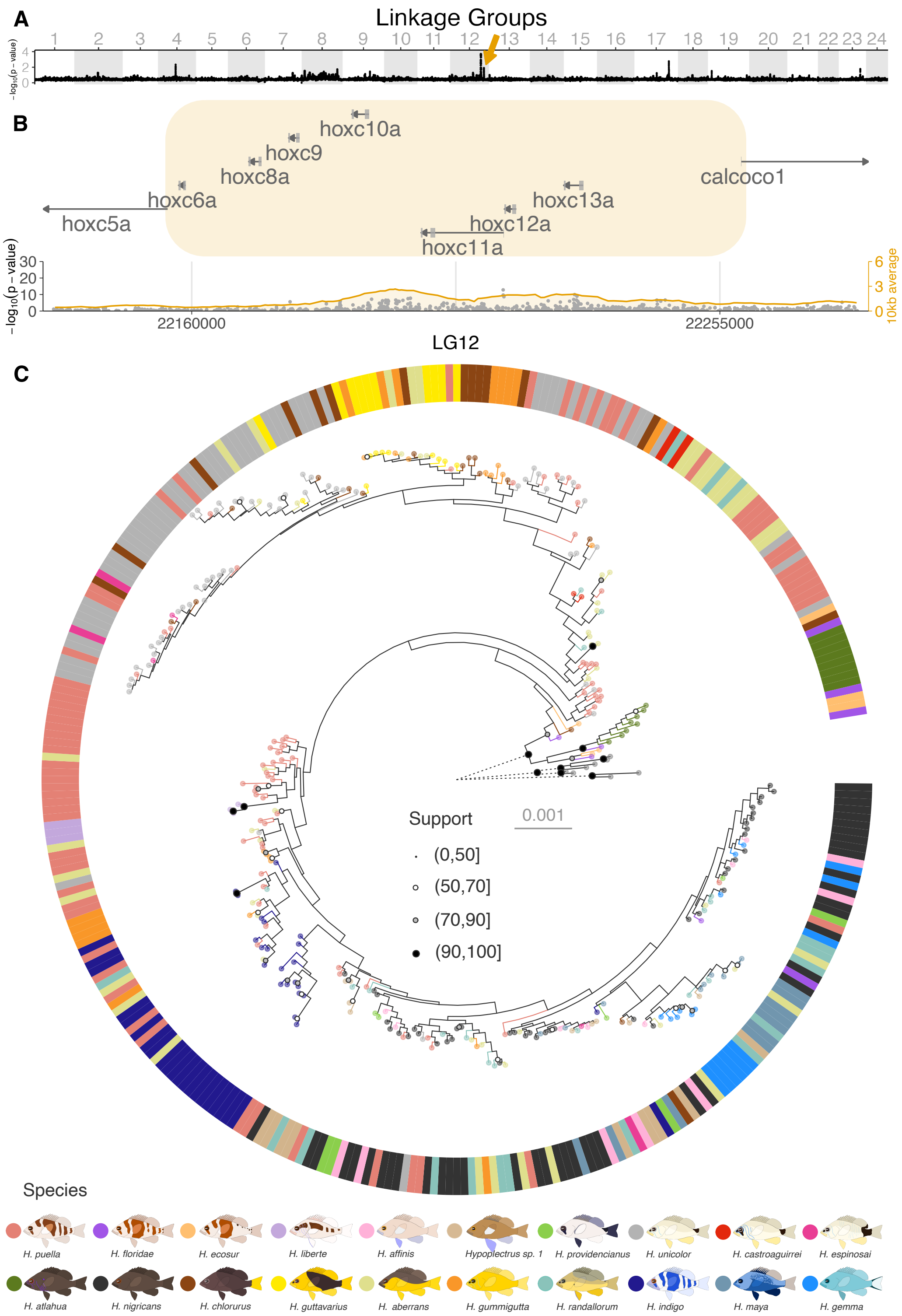

A
Linkage Groups
1 2 3 4 5 6 7 8 9 10 11 12 13 14 15 16 17 18 19 20 21 22 23 24
-log10(p-value)
B
hoxc10a
hoxc9
hoxc8a
hoxc6a
hoxc5a
hoxc13a
hoxc12a
hoxc11a
calcoco1
-log10(p-value)
10kb average
22160000
22255000
LG12
C
Support
0.001
(0,50]
(50,70]
(70,90]
(90,100]
Species
H. puella
H. floridae
H. ecosur
H. liberte
H. affinis
Hypoplectrus sp. 1
H. providencianus
H. unicolor
H. castroaguirrei
H. espinosai
H. atlahua
H. nigricans
H. chlorurus
H. guttavarius
H. aberrans
H. gummigutta
H. randallorum
H. indigo
H. maya
H. gemma

Fig. S16. GWAS for species identity and phylogeny of the second LG12 association peak. (**A**) Results of the genome-wide association study (GWAS) for species identity. The gray and white blocks represent the 24 linkage groups (LG, putative chromosomes) and the black dots are averages of the log-transformed *p*-value of the GWAS association over 50 kb windows with 5 kb increments. (**B**) Close-up on the second LG12 association peak. The x-axis shows the position on LG12 (in bp) and the orange rectangle highlights the region that shows an association $>$ 1.5 considering 50 kb windows. gray dots represent the GWAS log-transformed *p* value for each individual SNP, and the orange line its average over 10 kb windows. (**C**) Phylogeny of the second LG12 region, covering 88,334 bp centered on the GWAS association peak highlighted in orange in **B**. The outer ring, branch and tip point colors indicate hamlet species as shown by the icons below the tree. Node point size and color represent node support. The eight *Serranus* outgroup individuals are shown in gray and the length of their branches, represented with dashed lines, are reduced to 5 % of their actual length to improve the visualization of the hamlet radiation.

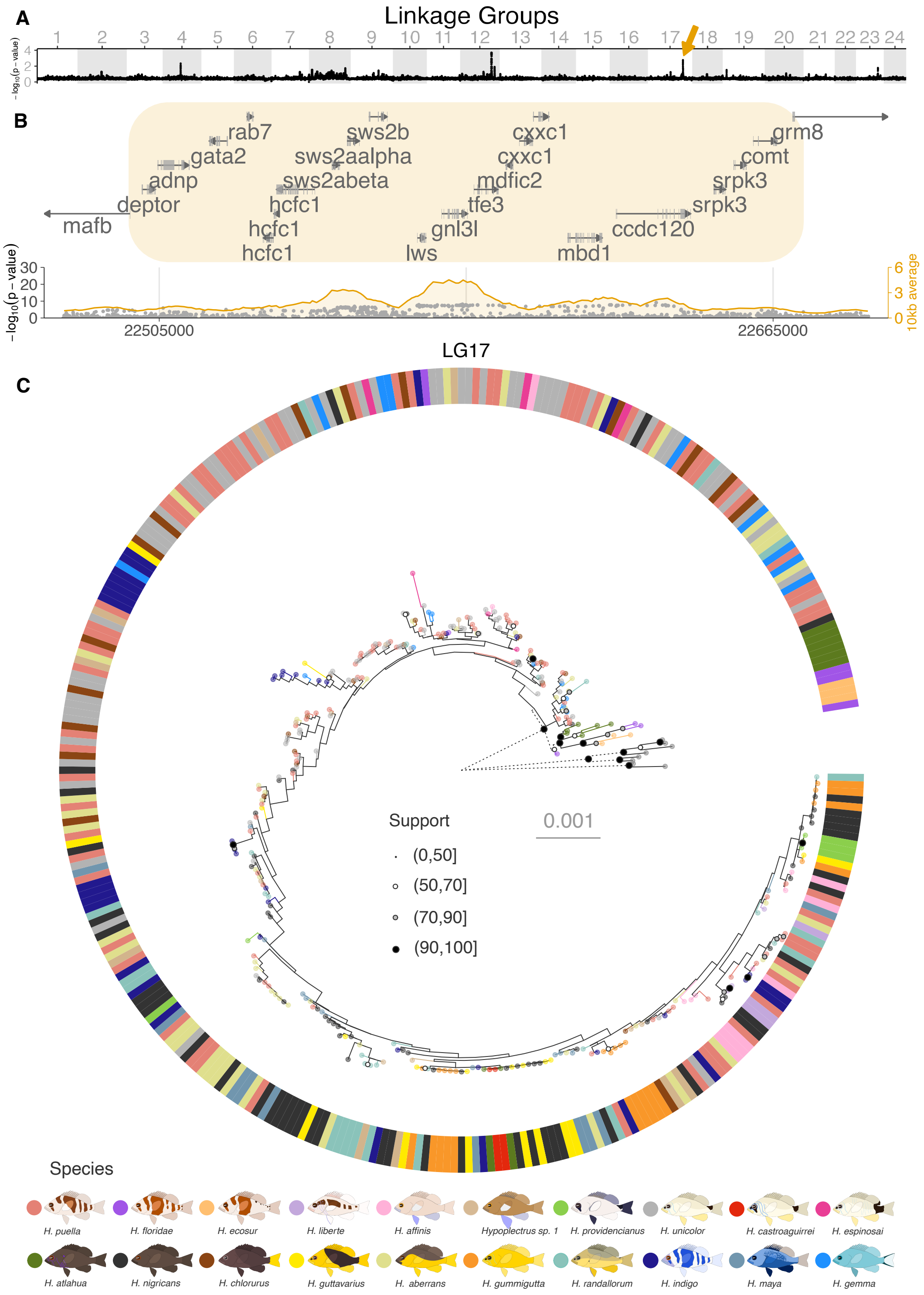
A
Linkage Groups
1 2 3 4 5 6 7 8 9 10 11 12 13 14 15 16 17 18 19 20 21 22 23 24
-log10(p-value)
B
rab7
gata2
adnp
deptor
mafb
sws2b
sws2aalpha
sws2abeta
hcfc1
hcfc1
hcfc1
cxxc1
cxxc1
mdfic2
tfe3
gnl3l
lws
mbd1
ccdc120
srpk3
srpk3
comt
grm8
-log10(p-value)
10kb average
22505000
22665000
LG17
C
Support
0.001
(0,50]
(50,70]
(70,90]
(90,100]
Species
H. puella
H. floridae
H. ecosur
H. liberte
H. affinis
Hypoplectrus sp. 1
H. providencianus
H. unicolor
H. castroaguirrei
H. espinosai
H. atlahua
H. nigricans
H. chlorurus
H. guttavarius
H. aberrans
H. gummigutta
H. randallorum
H. indigo
H. maya
H. gemma

Fig. S17. GWAS for species identity and phylogeny of the LG17 association peak. (**A**) Results of the genome-wide association study (GWAS) for species identity. The gray and white blocks represent the 24 linkage groups (LG, putative chromosomes) and the black dots are averages of the log-transformed *p*-value of the GWAS association over 50 kb windows with 5 kb increments. (**B**) Close-up on the LG17 association peak. The x-axis shows the position on LG17 (in bp) and the orange rectangle highlights the region that shows an association > 1.5 considering 50 kb windows. gray dots represent the GWAS log-transformed *p* value for each individual SNP, and the orange line its average over 10 kb windows. (**C**) Phylogeny of the LG17 region, covering 146,194 bp centered on the GWAS association peak highlighted in orange in **B**. The outer ring, branch and tip point colors indicate hamlet species as shown by the icons below the tree. Node point size and color represent node support. The eight *Serranus* outgroup individuals are shown in gray and the length of their branches, represented with dashed lines, are reduced to 5 % of their actual length to improve the visualization of the hamlet radiation.

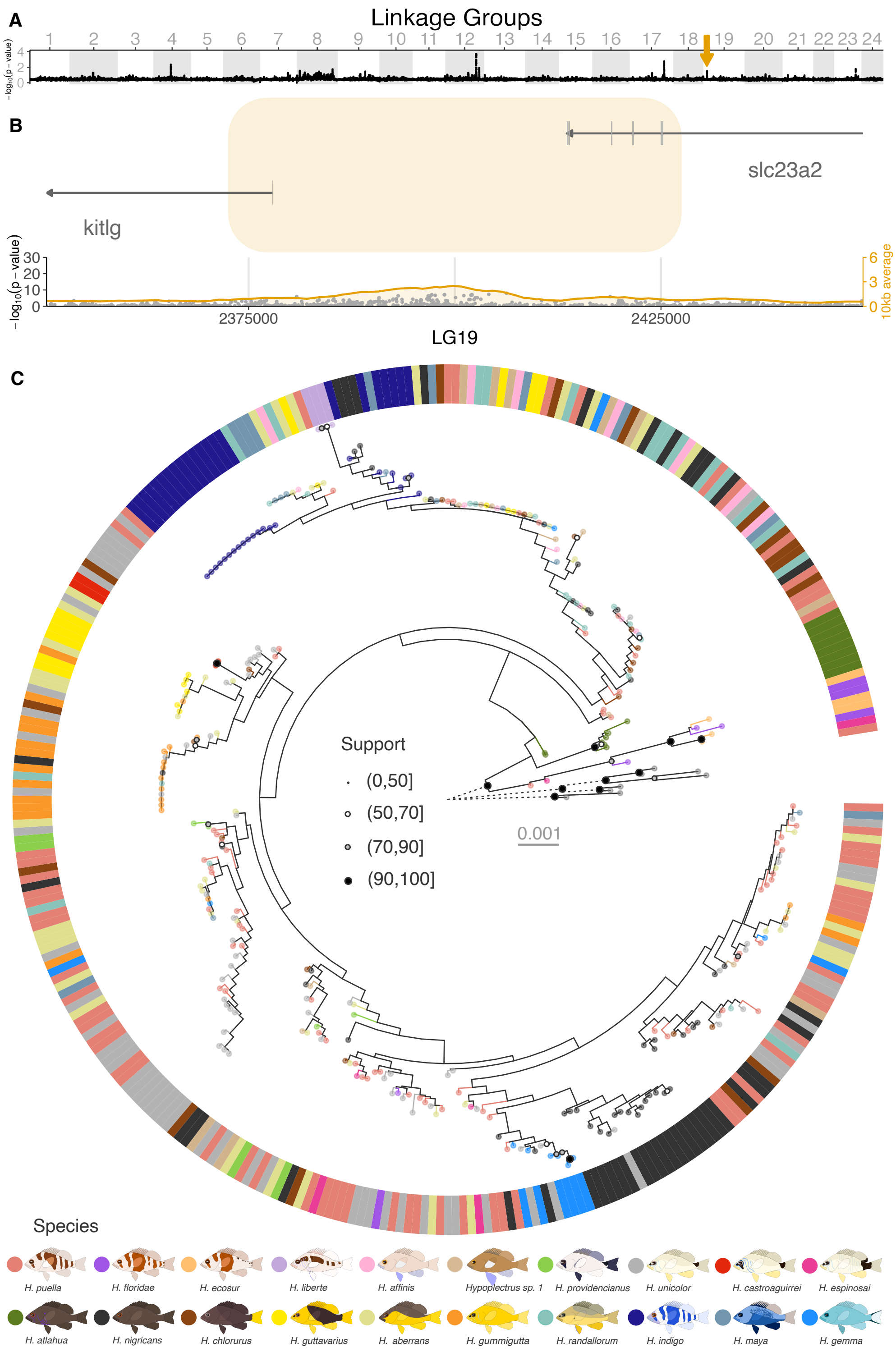
A
Linkage Groups
1 2 3 4 5 6 7 8 9 10 11 12 13 14 15 16 17 18 19 20 21 22 23 24
B
slc23a2
kitlg
−log10(p − value)
10kb average
2375000
2425000
LG19
C
Support
(0,50]
(50,70]
(70,90]
(90,100]
0.001
Species
H. puella
H. floridae
H. ecosur
H. liberte
H. affinis
Hypoplectrus sp. 1
H. providencianus
H. unicolor
H. castroaguirrei
H. espinosai
H. atlahua
H. nigricans
H. chlorurus
H. guttavarius
H. aberrans
H. gummigutta
H. randallorum
H. indigo
H. maya
H. gemma

Fig. S18. GWAS for species identity and phylogeny of the LG19 association peak. (**A**) Results of the genome-wide association study (GWAS) for species identity. The gray and white blocks represent the 24 linkage groups (LG, putative chromosomes) and the black dots are averages of the log-transformed *p*-value of the GWAS association over 50 kb windows with 5 kb increments. (**B**) Close-up on the LG19 association peak. The x-axis shows the position on LG19 (in bp) and the orange rectangle highlights the region that shows an association $>$ 1.5 considering 50 kb windows. gray dots represent the GWAS log-transformed *p* value for each individual SNP, and the orange line its average over 10 kb windows. (**C**) Phylogeny of the LG19 region, covering 44,506 bp centered on the GWAS association peak highlighted in orange in **B**. The outer ring, branch and tip point colors indicate hamlet species as shown by the icons below the tree. Node point size and color represent node support. The eight *Serranus* outgroup individuals are shown in gray and the length of their branches, represented with dashed lines, are reduced to 5 % of their actual length to improve the visualization of the hamlet radiation.

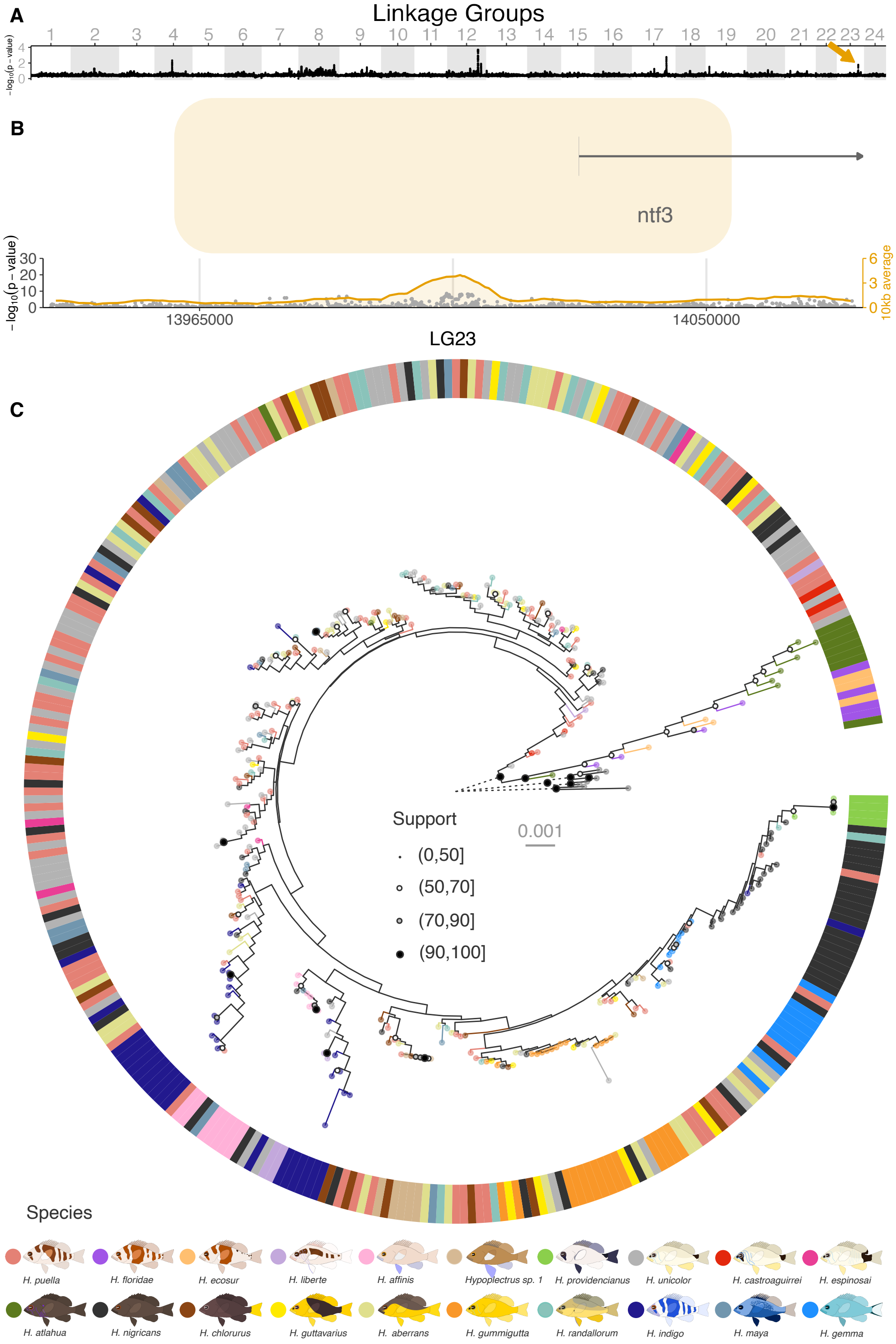
A
Linkage Groups
1 2 3 4 5 6 7 8 9 10 11 12 13 14 15 16 17 18 19 20 21 22 23 24
-log10(p-value)
B
ntf3
-log10(p-value)
10kb average
13965000
14050000
LG23
C
Support
(0,50]
(50,70]
(70,90]
(90,100]
0.001
Species
H. puella
H. floridae
H. ecosur
H. liberte
H. affinis
Hypoplectrus sp. 1
H. providencianus
H. unicolor
H. castroaguirrei
H. espinosai
H. atlahua
H. nigricans
H. chlorurus
H. guttavarius
H. aberrans
H. gummigutta
H. randallorum
H. indigo
H. maya
H. gemma

Fig. S19. GWAS for species identity and phylogeny of the LG23 association peak. (**A**) Results of the genome-wide association study (GWAS) for species identity. The gray and white blocks represent the 24 linkage groups (LG, putative chromosomes) and the black dots are averages of the log-transformed *p*-value of the GWAS association over 50 kb windows with 5 kb increments. (**B**) Close-up on the LG23 association peak. The x-axis shows the position on LG23 (in bp) and the orange rectangle highlights the region that shows an association $>$ 1.5 considering 50 kb windows. gray dots represent the GWAS log-transformed *p* value for each individual SNP, and the orange line its average over 10 kb windows. (**C**) Phylogeny of the LG23 region, covering 75,943 bp centered on the GWAS association peak highlighted in orange in **B**. The outer ring, branch and tip point colors indicate hamlet species as shown by the icons below the tree. Node point size and color represent node support. The eight *Serranus* outgroup individuals are shown in gray and the length of their branches, represented with dashed lines, are reduced to 5 % of their actual length to improve the visualization of the hamlet radiation.

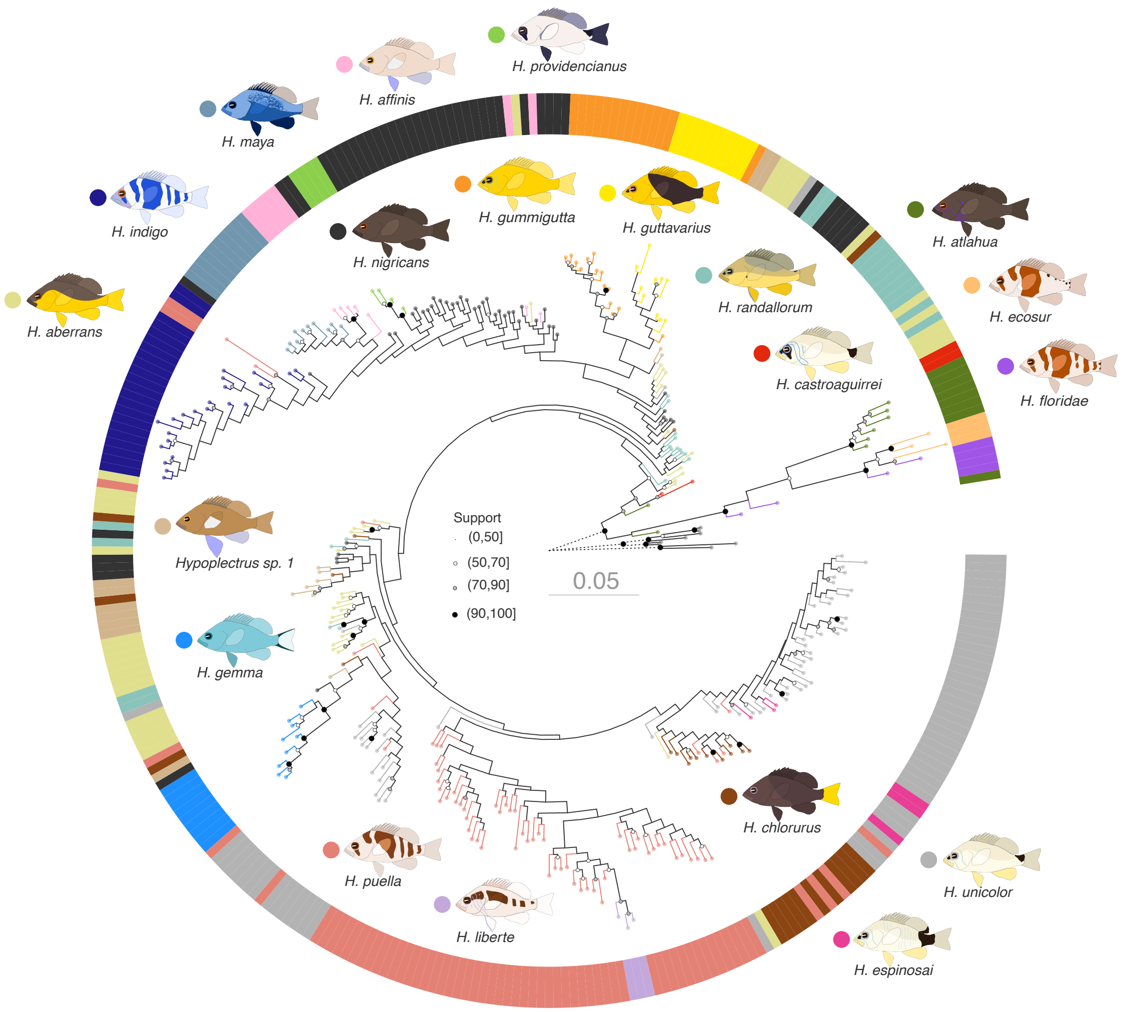

H. providencianus
H. affinis
H. maya
H. indigo
H. aberrans
H. nigricans
H. gummigutta
H. guttavarius
H. atlahua
H. randallorum
H. ecosur
H. castroaguirrei
H. floridae
Support
(0,50]
(50,70]
(70,90]
(90,100]
0.05
Hypoplectrus sp. 1
H. gemma
H. chlorurus
H. puella
H. liberte
H. unicolor
H. espinosai

Fig. S20. Phylogeny of the eight concatenated regions with high association to species identity. These are the eight genomic regions with an association -log(pvalue) > 1.5 considering 50 kb windows (Figs. 3, S13-S19), covering a total of 762,681 bp. The outer ring, branch and tip point colors indicate hamlet species as shown by the icons around the tree. Node point size and color represent node support. The eight *Serranus* outgroup individuals are shown in gray and the length of their branches, represented with dashed lines, are reduced to 0.5 % of their actual length to improve the visualization of the hamlet radiation.

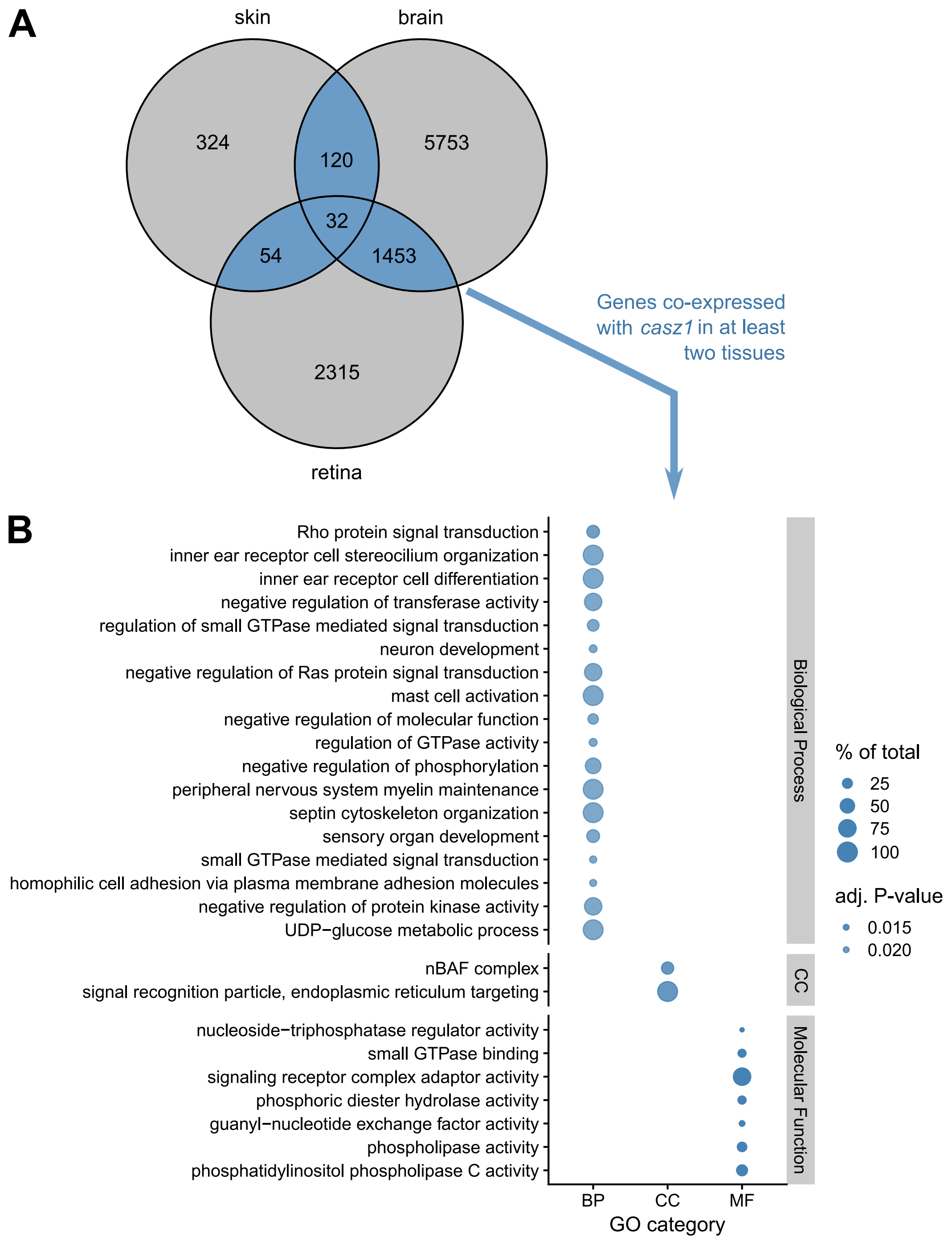
A
skin
brain
324
120
5753
32
54
1453
2315
retina
Genes co-expressed with casz1 in at least two tissues
B
Rho protein signal transduction
inner ear receptor cell stereocilium organization
inner ear receptor cell differentiation
negative regulation of transferase activity
regulation of small GTPase mediated signal transduction
neuron development
negative regulation of Ras protein signal transduction
mast cell activation
negative regulation of molecular function
regulation of GTPase activity
negative regulation of phosphorylation
peripheral nervous system myelin maintenance
septin cytoskeleton organization
sensory organ development
small GTPase mediated signal transduction
homophilic cell adhesion via plasma membrane adhesion molecules
negative regulation of protein kinase activity
UDP−glucose metabolic process
nBAF complex
signal recognition particle, endoplasmic reticulum targeting
nucleoside−triphosphatase regulator activity
small GTPase binding
signaling receptor complex adaptor activity
phosphoric diester hydrolase activity
guanyl−nucleotide exchange factor activity
phospholipase activity
phosphatidylinositol phospholipase C activity
Biological Process
CC
Molecular Function
BP
CC
MF
GO category
% of total
25
50
75
100
adj. P-value
0.015
0.020

Fig. S21. Genes co-expressed with *casz1*. (**A**) Venn diagram showing the number of genes co-expressed with *casz1* in brain, retina and skin tissue. (**B**) Top 10 % enriched Gene Ontology (GO) terms for genes that are co-expressed with *casz1* in at least two tissues, ordered by increasing adjusted P-value per GO category. The circle size indicates the percentage of co-expressed genes relative to the number of genes that are annotated with this term. The GO categories are biological processes (BP), cellular components (CC) and molecular functions (MF).

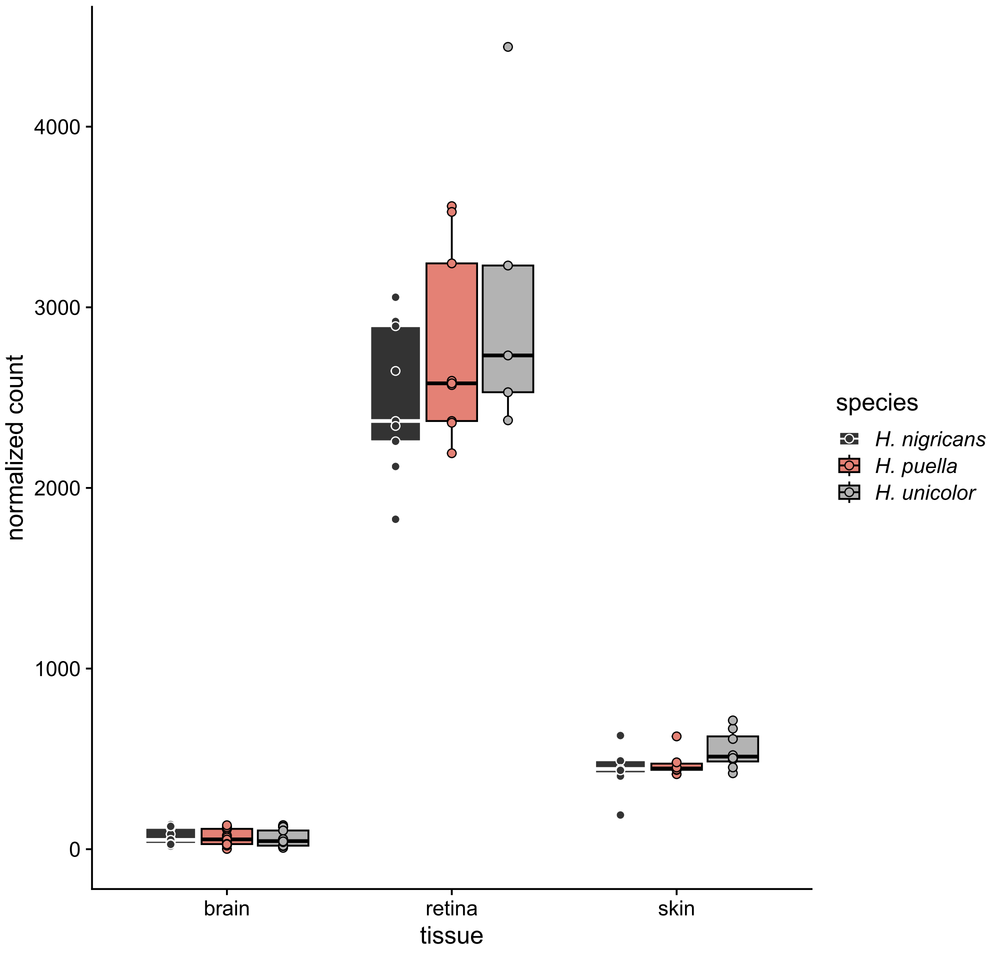

Fig. S22. Normalized expression of *casz1* across tissues and species. Boxplots display the median (center line), the 25th and 75th percentiles (box limits), the largest value within 1.5× the inter-quartile range (whiskers) and outliers (points) with sample points plotted on top. The sample sizes are: seven skin samples per species; 18, 18 and 17 brain samples from *H. puella*, *H. unicolor* and *H. nigricans*, respectively; and 9, 5 and 9 retinal samples from *H. puella*, *H. unicolor* and *H. nigricans*, respectively.

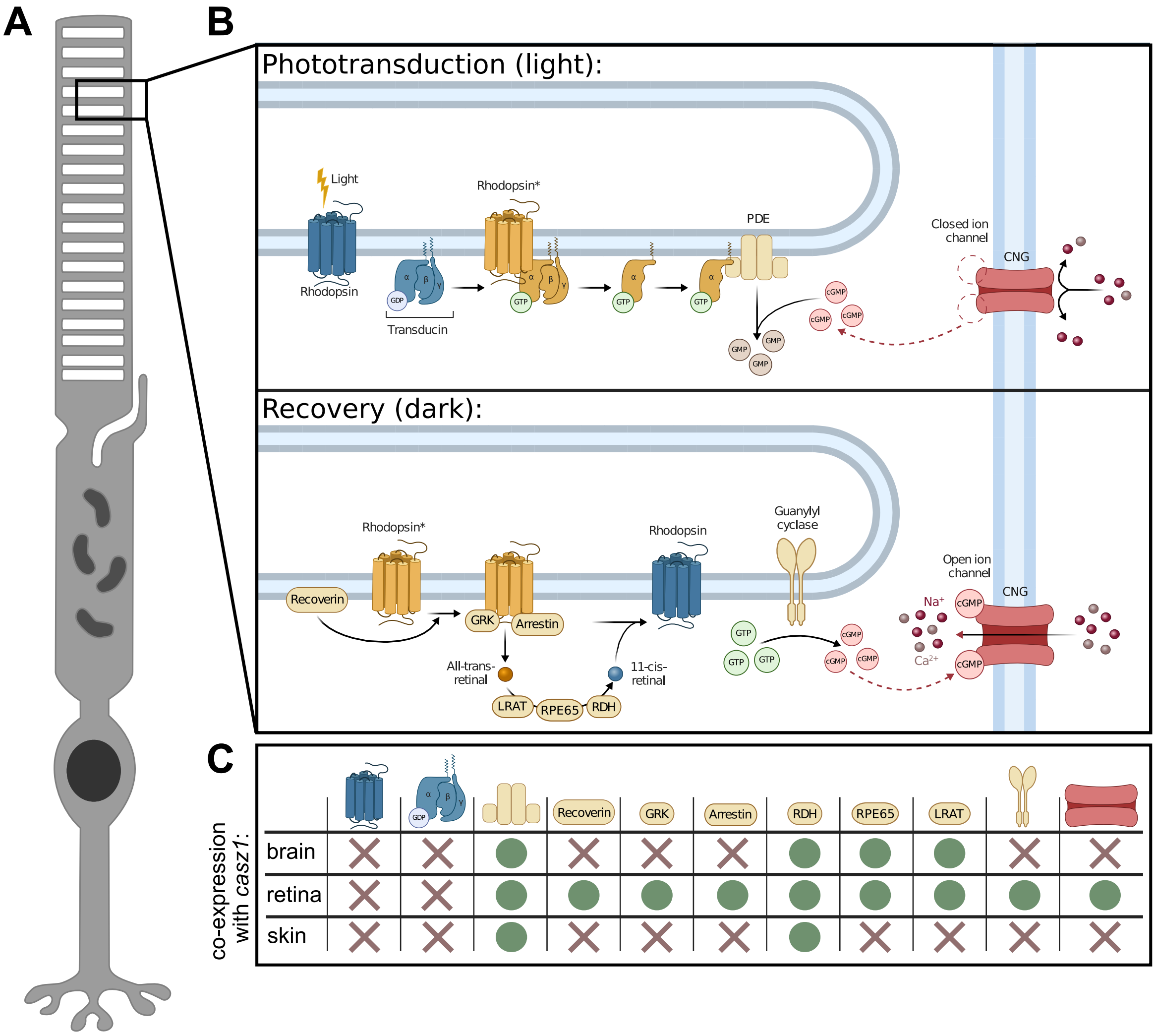

Fig. S23. Phototransduction cascade. (**A**) Illustration of a rod cell in the retinal tissue. (**B**) Genes involved in the phototransduction cascade during light and recovery phase. (**C**) Genes that are co-expressed with *casz1* across the three different tissues are highlighted with a green dot.

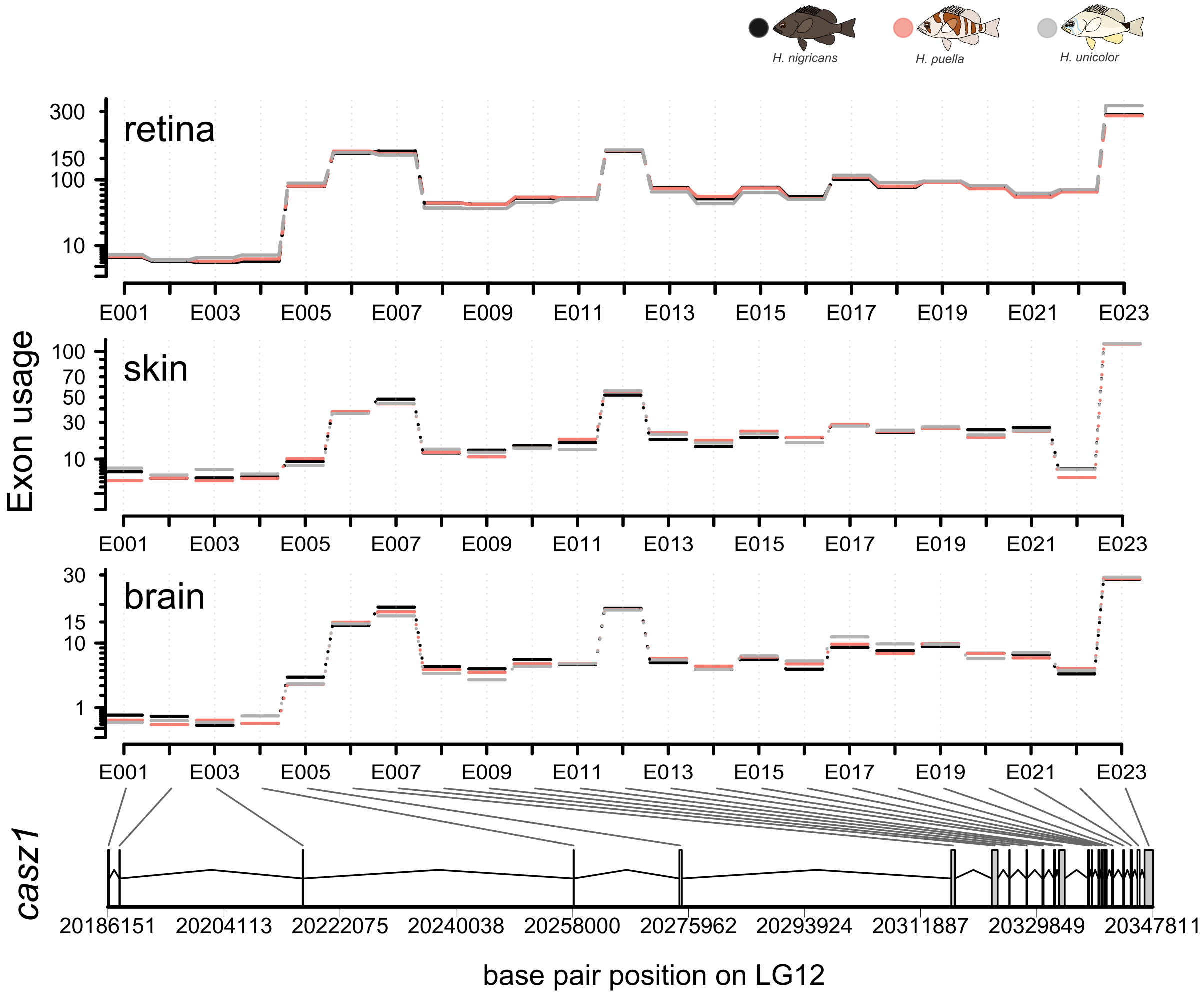

Fig. S24. ***casz1* exon usage.** The three plots show the normalized expression of *casz1* exons relative to the gene mean.

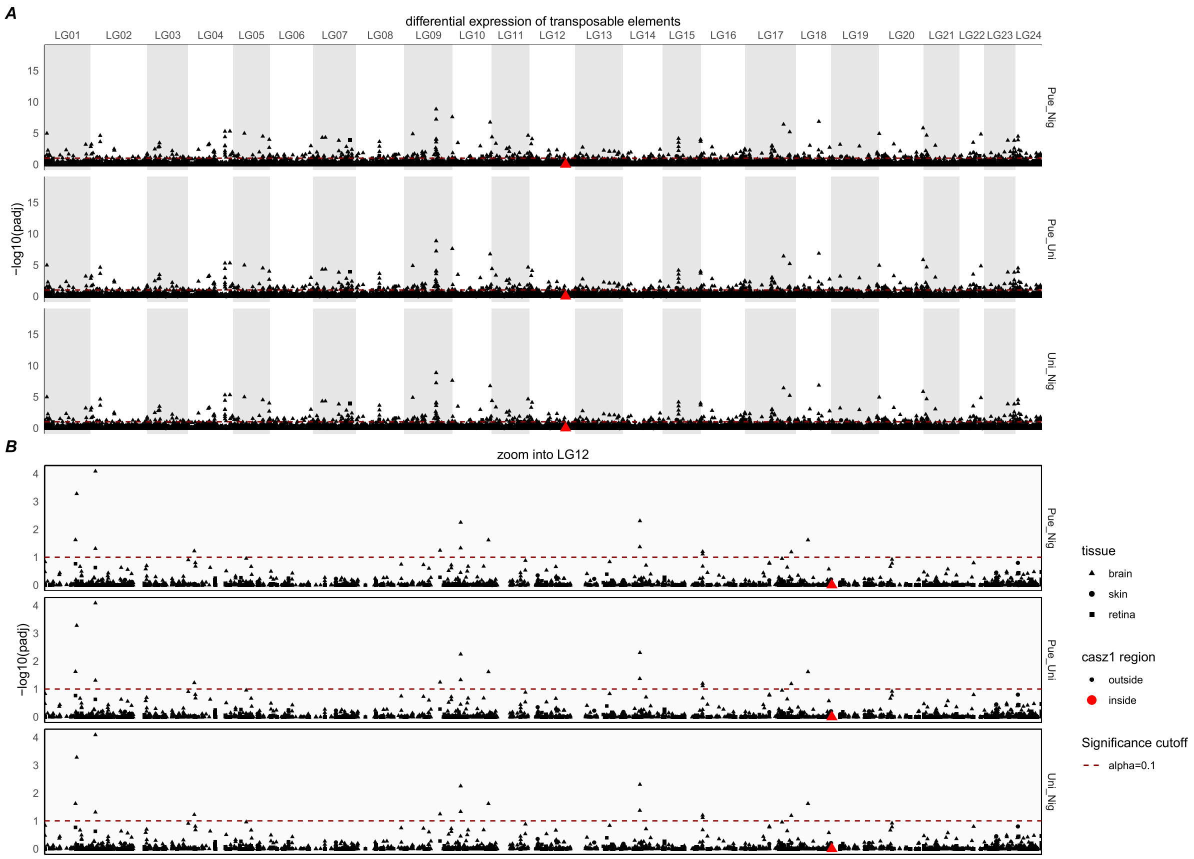

Fig. S25. Differential expression of transposable elements between species. Pue: *H. puella*, Nig: *H. nigricans*, Uni: *H. unicolor*). Adjusted log transformed P-values were plotted along the genome (**A**) and for LG12 (**B**). The *casz1* region is highlighted in red.

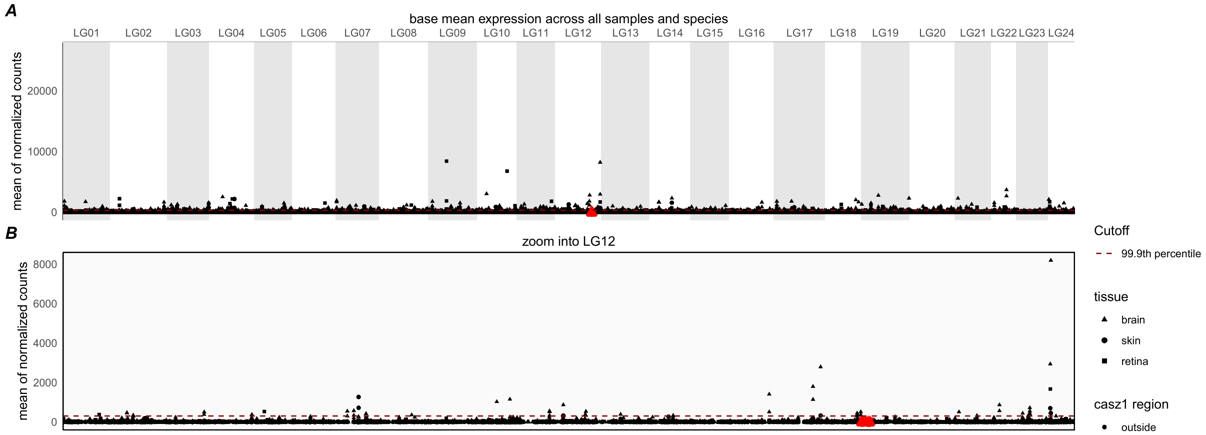

Fig. S26. Normalized expression of transposable elements. (**A**) Along the genome and (**B**) in LG12 across species (*H. puella, H. nigricans, H. unicolor*). High expression values indicate mobilized transposable elements. The *casz1* region is highlighted in red.

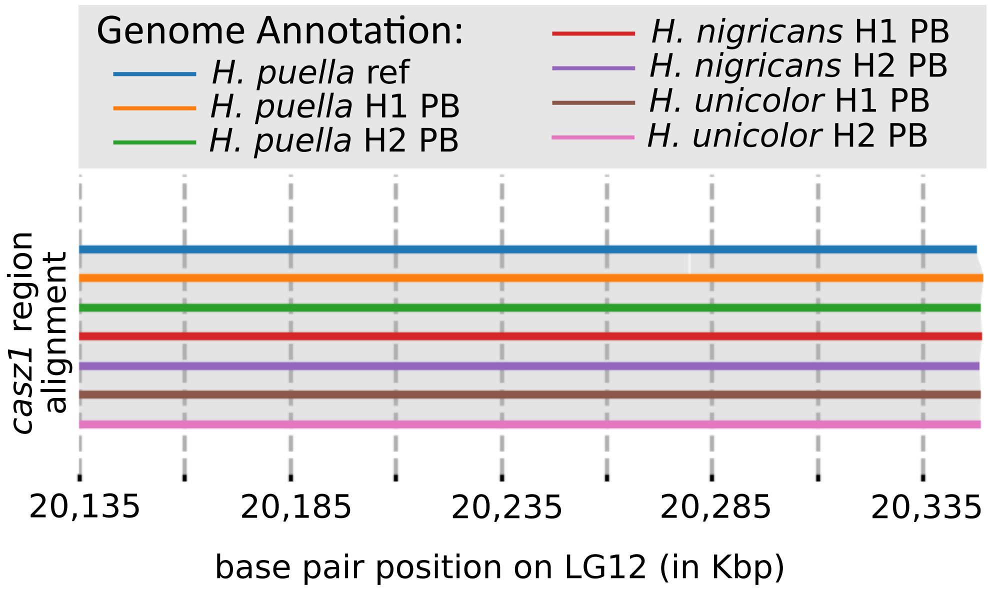

Fig. S27. Structural variation in the *casz1* region. Each line represents a sequence from six PacBio genomes (two haplotypes per genome, H1 PB and H2 PB) and the *H. puella* reference genome. The base pair position on the y-axis is relative to the reference genome assembly (blue). The gray areas between the sequences (colored lines) indicate that they align without major gaps or rearrangements. A Fasta file with the *casz1* region alignment can be found in the GitHub repository (github.com/mhelmkampf/hamlet_phylogeny).

Supplementary Tables

Table S1. List of resequenced genomes used in this study, including 327 hamlet and eight outgroup samples. Date, latitude and longitude refer to collection dates and coordinates. Accession gives the sample record in the European Nucleotide Archive (ENA) for each sample, with asterisks indicating samples for which new data has been generated and submitted to ENA (note that data from previous sequencing runs may be attached to some samples, but only data from the latest runs were used in this study). Col.: Colombia, Mex.: Mexico, Pan.: Panama.

Sample ID	Species	Location	Date	Latitude	Longitude	Accession
13310	*H. puella*	Quintana Roo, Mex.	2007-01-08	20.45344	-87.26467	ERS20924417*
13318	*H. unicolor*	Quintana Roo, Mex.	2007-01-08	20.45633	-87.26217	ERS20924418*
17996	*H. indigo*	Belize	2004-07-27	16.80083	-88.07889	ERS4141229
17997	*H. indigo*	Belize	2004-07-27	16.80083	-88.07889	ERS4141230
17998	*H. indigo*	Belize	2004-07-27	16.80083	-88.07889	ERS4141231
17999	*H. indigo*	Belize	2004-07-27	16.80083	-88.07889	ERS4141232
18000	*H. indigo*	Belize	2004-07-27	16.80083	-88.07889	ERS4141233
18151	*H. nigricans*	Belize	2004-07-25	16.76528	-88.14417	ERS2619600
18152	*H. puella*	Belize	2004-07-25	16.76528	-88.14417	ERS2619637
18153	*H. nigricans*	Belize	2004-07-25	16.76528	-88.14417	ERS2619601
18154	*H. puella*	Belize	2004-07-25	16.76528	-88.14417	ERS2619638
18155	*H. nigricans*	Belize	2004-07-25	16.80083	-88.07889	ERS2619602
18156	*H. nigricans*	Belize	2004-07-25	16.80083	-88.07889	ERS2619603
18157	*H. nigricans*	Belize	2004-07-25	16.80083	-88.07889	ERS2619604
18158	*H. nigricans*	Belize	2004-07-25	16.80083	-88.07889	ERS2619605
18159	*H. nigricans*	Belize	2004-07-25	16.80083	-88.07889	ERS2619606
18160	*H. guttavarius*	Belize	2004-07-27	16.78389	-88.07667	ERS20924419*
18161	*H. puella*	Belize	2004-07-26	16.80583	-88.07917	ERS2619639
18162	*H. nigricans*	Belize	2004-07-25	16.76528	-88.14417	ERS2619607
18163	*H. unicolor*	Belize	2004-07-25	16.76528	-88.14417	ERS2619674
18165	*H. nigricans*	Belize	2004-07-25	16.76528	-88.14417	ERS2619608
18166	*H. puella*	Belize	2004-07-25	16.76528	-88.14417	ERS2619640
18169	*H. puella*	Belize	2004-07-25	16.76528	-88.14417	ERS2619641

**Sample ID**	**Species**	**Location**	**Date**	**Latitude**	**Longitude**	**Accession**
18171	*H. nigricans*	Belize	2004-07-25	16.76528	-88.14417	ERS2619609
18172	*H. puella*	Belize	2004-07-25	16.76528	-88.14417	ERS2619642
18174	*H. puella*	Belize	2004-07-25	16.76528	-88.14417	ERS2619643
18175	*H. puella*	Belize	2004-07-25	16.76528	-88.14417	ERS2619644
18176	*H. puella*	Belize	2004-07-25	16.76528	-88.14417	ERS2619645
18178	*H. puella*	Belize	2004-07-25	16.76528	-88.14417	ERS2619646
18179	*H. puella*	Belize	2004-07-25	16.76528	-88.14417	ERS2619647
18180	*H. puella*	Belize	2004-07-25	16.76528	-88.14417	ERS2619648
18185	*H. nigricans*	Belize	2004-07-26	16.80583	-88.07917	ERS2619610
18187	*H. nigricans*	Belize	2004-07-26	16.80583	-88.07917	ERS2619611
18195	*H. indigo*	Belize	2004-07-26	16.80583	-88.07917	ERS4141234
18222	*H. indigo*	Belize	2004-07-27	16.80083	-88.07889	ERS4141235
18225	*H. indigo*	Belize	2004-07-27	16.80083	-88.07889	ERS4141236
18226	*H. indigo*	Belize	2004-07-27	16.78389	-88.07667	ERS4141237
18227	*H. indigo*	Belize	2004-07-27	16.78389	-88.07667	ERS4141238
18261	*H. unicolor*	Belize	2004-07-24	NA	NA	ERS2619675
18263	*H. aberrans*	Belize	2004-07-24	NA	NA	ERS20924420*
18267	*H. unicolor*	Belize	2004-07-24	NA	NA	ERS2619676
18274	*H. unicolor*	Belize	2004-07-24	NA	NA	ERS2619677
18276	*H. unicolor*	Belize	2004-07-25	16.76528	-88.14417	ERS2619678
18418	*H. nigricans*	Bocas del Toro, Pan.	2004-05-12	9.37750	-82.30389	ERS2619625
18419	*H. puella*	Bocas del Toro, Pan.	2004-05-12	10.23917	-83.17306	ERS2619661
18420	*H. unicolor*	Bocas del Toro, Pan.	2004-05-12	10.23917	-83.17306	ERS2619698
18421	*H. puella*	Bocas del Toro, Pan.	2004-05-12	10.23917	-83.17306	ERS2619662
18422	*H. puella*	Bocas del Toro, Pan.	2004-05-12	9.37750	-82.30389	ERS2619663
18424	*H. nigricans*	Bocas del Toro, Pan.	2004-05-12	10.23917	-83.17306	ERS2619626
18426	*H. puella*	Bocas del Toro, Pan.	2004-05-12	10.23917	-83.17306	ERS2619664
18427	*H. puella*	Bocas del Toro, Pan.	2004-05-12	10.23917	-83.17306	ERS2619665
18428	*H. nigricans*	Bocas del Toro, Pan.	2004-05-12	10.23917	-83.17306	ERS2619627
18429	*H. puella*	Bocas del Toro, Pan.	2004-05-12	10.23917	-83.17306	ERS2619666
18430	*H. puella*	Bocas del Toro, Pan.	2004-05-12	10.23917	-83.17306	ERS2619667
18432	*H. puella*	Bocas del Toro, Pan.	2004-05-12	10.23917	-83.17306	ERS2619668
18434	*H. puella*	Bocas del Toro, Pan.	2004-05-12	9.37750	-82.30389	ERS2619669
18435	*H. unicolor*	Bocas del Toro, Pan.	2004-06-23	9.33278	-82.25472	ERS2619699
18439	*H. unicolor*	Bocas del Toro, Pan.	2004-06-25	9.33278	-82.25472	ERS2619700
18440	*H. unicolor*	Bocas del Toro, Pan.	2004-06-25	9.33278	-82.25472	ERS2619701
18441	*H. unicolor*	Bocas del Toro, Pan.	2004-06-25	9.33278	-82.25472	ERS2619702
18442	*H. unicolor*	Bocas del Toro, Pan.	2004-07-08	9.29833	-82.28944	ERS2619703

Sample ID	Species	Location	Date	Latitude	Longitude	Accession
18445	*H. unicolor*	Bocas del Toro, Pan.	2004-06-28	9.33278	-82.25472	ERS2619704
18446	*H. unicolor*	Bocas del Toro, Pan.	2004-06-29	9.33278	-82.25472	ERS2619705
18447	*H. unicolor*	Bocas del Toro, Pan.	2004-07-09	9.28944	-82.25889	ERS2619706
18448	*H. unicolor*	Bocas del Toro, Pan.	2004-06-28	9.33278	-82.25472	ERS2619707
18449	*H. gummigutta*	Bocas del Toro, Pan.	2004-07-03	9.37750	-82.30389	ERS2619710
18450	*H. unicolor*	Bocas del Toro, Pan.	2004-06-25	9.33278	-82.25472	ERS2619708
18452	*H. aberrans*	Bocas del Toro, Pan.	2004-07-02	9.33083	-82.21444	ERS20924421*
18454	*H. unicolor*	Bocas del Toro, Pan.	2004-06-30	9.34806	-82.26333	ERS2619709
18471	*H. aberrans*	Bocas del Toro, Pan.	2004-07-10	9.37750	-82.30389	ERS20924422*
18492	*H. aberrans*	Belize	2004-07-24	16.89389	-88.05806	ERS20924423*
18493	*H. aberrans*	Belize	2004-07-24	NA	NA	ERS20924424*
18901	*H. nigricans*	Bocas del Toro, Pan.	2005-03-25	9.29833	-82.28944	ERS2619629
18902	*H. nigricans*	Bocas del Toro, Pan.	2005-03-25	9.29833	-82.28944	ERS2619630
18903	*H. nigricans*	Bocas del Toro, Pan.	2005-03-25	9.29833	-82.28944	ERS2619631
18904	*H. nigricans*	Bocas del Toro, Pan.	2005-03-25	9.29833	-82.28944	ERS2619632
18905	*H. nigricans*	Bocas del Toro, Pan.	2005-03-25	9.29833	-82.28944	ERS2619633
18906	*H. nigricans*	Bocas del Toro, Pan.	2005-03-25	9.29833	-82.28944	ERS2619634
18907	*H. nigricans*	Bocas del Toro, Pan.	2005-03-25	9.29833	-82.28944	ERS2619635
18909	*H. nigricans*	Bocas del Toro, Pan.	2005-03-25	9.29833	-82.28944	ERS2619636
18912	*H. puella*	Bocas del Toro, Pan.	2005-03-25	9.29833	-82.28944	ERS2619670
18915	*H. puella*	Bocas del Toro, Pan.	2005-03-25	9.29833	-82.28944	ERS2619671
18917	*H. puella*	Bocas del Toro, Pan.	2005-03-25	9.29833	-82.28944	ERS2619672
18935	*H. affinis*	Bocas del Toro, Pan.	2005-03-27	9.25081	-82.13061	ERS20924425*
18936	*H. affinis*	Bocas del Toro, Pan.	2005-03-27	9.25081	-82.13061	ERS20924426*
18949	*H. aberrans*	Guna Yala, Pan.	2005-05-04	9.54875	-78.95061	ERS20924427*
18986	*H. affinis*	Bocas del Toro, Pan.	2005-03-25	9.29833	-82.28944	ERS8632037
19076	*H. guttavarius*	Barbados	2005-06-09	13.22619	-59.65286	ERS20924428*
19077	*H. sp1*	Barbados	2005-06-09	13.22619	-59.65286	ERS20924429*
19079	*H. sp1*	Barbados	2005-06-10	13.13375	-59.64072	ERS20924430*
19080	*H. guttavarius*	Barbados	2005-06-12	13.17264	-59.64675	ERS20924431*
19104	*H. puella*	Guna Yala, Pan.	2005-05-08	9.57592	-78.72794	ERS20924432*
19105	*H. nigricans*	Guna Yala, Pan.	2005-05-08	9.57592	-78.72794	ERS20924433*
19106	*H. puella*	Guna Yala, Pan.	2005-05-08	9.57592	-78.72794	ERS20924434*
19108	*H. puella*	Guna Yala, Pan.	2005-05-08	9.57592	-78.72794	ERS20924435*
19124	*H. indigo*	Guna Yala, Pan.	2005-05-08	9.57592	-78.72794	ERS20924436*
19131	*H. nigricans*	Guna Yala, Pan.	2005-05-09	9.47431	-78.52164	ERS20924437*
19150	*H. nigricans*	Guna Yala, Pan.	2005-05-09	9.47431	-78.52164	ERS20924438*
19162	*H. indigo*	Guna Yala, Pan.	2005-05-07	9.58019	-78.69406	ERS20924439*

Sample ID	Species	Location	Date	Latitude	Longitude	Accession
19174	*H. affinis*	Guna Yala, Pan.	2005-05-07	9.58019	-78.69406	ERS20924440*
19190	*H. indigo*	Guna Yala, Pan.	2005-05-08	9.57592	-78.72794	ERS20924441*
19235	*H. guttavarius*	Guna Yala, Pan.	2005-05-15	9.19967	-77.96972	ERS20924442*
19294	*H. affinis*	Guna Yala, Pan.	2005-05-22	9.57647	-78.72912	ERS20924443*
19416	*H. chlorurus*	Barbados	2005-06-06	13.22369	-59.64506	ERS20924444*
19425	*H. chlorurus*	Barbados	2005-06-06	13.22369	-59.64506	ERS20924445*
19426	*H. puella*	Barbados	2005-06-06	13.22369	-59.64506	ERS20924446*
19427	*H. puella*	Barbados	2005-06-06	13.22369	-59.64506	ERS20924447*
19428	*H. puella*	Barbados	2005-06-06	13.22369	-59.64506	ERS20924448*
19435	*H. chlorurus*	Barbados	2005-06-06	13.22369	-59.64506	ERS20924449*
19437	*H. sp1*	Barbados	2005-06-06	13.22369	-59.64506	ERS20924450*
19519	*H. affinis*	Guna Yala, Pan.	2005-05-14	9.29072	-78.14039	ERS20924451*
19546	*H. aberrans*	Guna Yala, Pan.	2005-05-14	9.29072	-78.14039	ERS20924452*
19708	*H. aberrans*	Guna Yala, Pan.	2005-05-06	9.54875	-78.95061	ERS20924453*
19881	*H. unicolor*	Belize	2005-08-16	16.70781	-87.85981	ERS2619679
20068	*H. guttavarius*	Belize	2005-08-13	NA	NA	ERS20924454*
20069	*H. guttavarius*	Belize	2005-08-13	NA	NA	ERS20924455*
20092	*H. unicolor*	Belize	2005-08-15	16.89364	-88.12256	ERS2619680
20120	*H. unicolor*	Belize	2005-08-11	16.80083	-88.07889	ERS2619681
20126	*H. unicolor*	Belize	2005-08-12	16.89364	-88.12256	ERS2619682
20128	*H. unicolor*	Belize	2005-08-12	16.89364	-88.12256	ERS2619683
20135	*H. unicolor*	Belize	2005-08-12	16.89364	-88.12256	ERS2619684
20149	*H. unicolor*	Belize	2005-08-12	16.89364	-88.12256	ERS2619685
20418	*H. gummigutta*	Honduras	2006-06-03	16.03000	-83.32861	ERS4141241
20419	*H. gummigutta*	Honduras	2006-06-03	16.03000	-83.32861	ERS4141242
20420	*H. gummigutta*	Honduras	2006-06-03	16.03000	-83.32861	ERS4141243
20421	*H. randallorum*	Honduras	2006-06-03	16.03000	-83.32861	ERS4141244
20425	*H. aberrans*	Honduras	2006-06-03	16.03000	-83.32861	ERS4141245
20426	*H. gummigutta*	Honduras	2006-06-04	15.95583	-83.29306	ERS4141246
20427	*H. gummigutta*	Honduras	2006-06-04	15.95583	-83.29306	ERS4141247
20428	*H. gummigutta*	Honduras	2006-06-04	15.95583	-83.29306	ERS4141248
20429	*H. randallorum*	Honduras	2006-06-04	15.95583	-83.29306	ERS4141249
20430	*H. randallorum*	Honduras	2006-06-04	15.95583	-83.29306	ERS4141250
20433	*H. aberrans*	Honduras	2006-06-04	15.95583	-83.29306	ERS4141251
20435	*H. chlorurus*	Honduras	2006-06-04	15.95583	-83.29306	ERS20924456*
20478	*S. tabacarius*	Honduras	2006-06-06	15.25000	-82.61700	ERS4141252
20480	*S. tabacarius*	Honduras	2006-06-07	15.25000	-82.61700	ERS20924457*
20481	*S. tigrinus*	Honduras	2006-06-07	15.25000	-82.61700	ERS20924458*

Sample ID	Species	Location	Date	Latitude	Longitude	Accession
20551	*H. puella*	Honduras	2006-06-04	15.95583	-83.29306	ERS2619649
20552	*H. puella*	Honduras	2006-06-04	15.95583	-83.29306	ERS2619650
20553	*H. puella*	Honduras	2006-06-04	15.95583	-83.29306	ERS2619651
20554	*H. puella*	Honduras	2006-06-04	15.95583	-83.29306	ERS2619652
20555	*H. puella*	Honduras	2006-06-04	15.95583	-83.29306	ERS2619653
20556	*H. puella*	Honduras	2006-06-04	15.95583	-83.29306	ERS2619654
20558	*H. puella*	Honduras	2006-06-04	15.95583	-83.29306	ERS2619655
20559	*H. puella*	Honduras	2006-06-04	15.95583	-83.29306	ERS2619656
20560	*H. unicolor*	Honduras	2006-06-04	15.95583	-83.29306	ERS2619686
20561	*H. unicolor*	Honduras	2006-06-04	15.95583	-83.29306	ERS2619687
20562	*H. unicolor*	Honduras	2006-06-04	15.95583	-83.29306	ERS2619688
20563	*H. unicolor*	Honduras	2006-06-04	15.95583	-83.29306	ERS2619689
20564	*H. unicolor*	Honduras	2006-06-04	15.95583	-83.29306	ERS2619690
20565	*H. unicolor*	Honduras	2006-06-04	15.95583	-83.29306	ERS2619691
20566	*H. unicolor*	Honduras	2006-06-04	15.95583	-83.29306	ERS2619692
20567	*H. unicolor*	Honduras	2006-06-04	15.95583	-83.29306	ERS2619693
20568	*H. unicolor*	Honduras	2006-06-04	15.95583	-83.29306	ERS2619694
20571	*H. unicolor*	Honduras	2006-06-04	15.95583	-83.29306	ERS2619695
20572	*H. unicolor*	Honduras	2006-06-04	15.95583	-83.29306	ERS2619696
20599	*H. nigricans*	Honduras	2006-06-04	15.95583	-83.29306	ERS2619612
20600	*H. nigricans*	Honduras	2006-06-04	15.95583	-83.29306	ERS2619613
20601	*H. nigricans*	Honduras	2006-06-04	15.95583	-83.29306	ERS2619614
20602	*H. nigricans*	Honduras	2006-06-04	15.95583	-83.29306	ERS2619615
20603	*H. nigricans*	Honduras	2006-06-04	15.95583	-83.29306	ERS2619616
20604	*H. nigricans*	Honduras	2006-06-04	15.95583	-83.29306	ERS2619617
20605	*H. nigricans*	Honduras	2006-06-04	15.95583	-83.29306	ERS2619618
20606	*H. nigricans*	Honduras	2006-06-04	15.95583	-83.29306	ERS2619619
20607	*H. nigricans*	Honduras	2006-06-04	15.95583	-83.29306	ERS2619620
20608	*H. nigricans*	Honduras	2006-06-04	15.95583	-83.29306	ERS2619621
20609	*H. nigricans*	Honduras	2006-06-04	15.95583	-83.29306	ERS2619622
20610	*H. nigricans*	Honduras	2006-06-04	15.95583	-83.29306	ERS2619623
20613	*H. randallorum*	Honduras	2006-06-05	15.95583	-83.29306	ERS4141253
20615	*H. gummigutta*	Honduras	2006-06-05	15.95583	-83.29306	ERS4141254
20617	*H. gummigutta*	Honduras	2006-06-05	15.95583	-83.29306	ERS4141255
20625	*H. puella*	Honduras	2006-06-05	15.95583	-83.29306	ERS2619657
20633	*H. puella*	Honduras	2006-06-05	15.95583	-83.29306	ERS2619658
20635	*H. puella*	Honduras	2006-06-05	15.95583	-83.29306	ERS2619659
20638	*H. puella*	Honduras	2006-06-05	15.95583	-83.29306	ERS2619660

Sample ID	Species	Location	Date	Latitude	Longitude	Accession
20641	*H. gummigutta*	Honduras	2006-06-05	15.95583	-83.29306	ERS4141256
20642	*H. gummigutta*	Honduras	2006-06-05	15.95583	-83.29306	ERS4141257
20643	*H. gummigutta*	Honduras	2006-06-05	15.95583	-83.29306	ERS4141258
20644	*H. aberrans*	Honduras	2006-06-05	15.95583	-83.29306	ERS4141259
20650	*H. providencianus*	Honduras	2006-06-06	15.25000	-82.61667	ERS20924459*
20696	*H. randallorum*	Honduras	2006-06-12	16.11028	-86.95389	ERS4141260
20751	*H. guttavarius*	Honduras	2006-06-06	15.25000	-82.61667	ERS20924460*
20757	*H. indigo*	Honduras	2006-06-06	15.25000	-82.61667	ERS20924461*
20759	*H. aberrans*	Honduras	2006-06-06	15.25000	-82.61667	ERS4141261
20761	*H. aberrans*	Honduras	2006-06-06	15.25000	-82.61667	ERS4141262
20762	*H. aberrans*	Honduras	2006-06-06	15.25000	-82.61667	ERS4141263
20845	*H. providencianus*	Honduras	2006-06-07	15.25000	-82.61667	ERS20924462*
20846	*H. providencianus*	Honduras	2006-06-07	15.25000	-82.61667	ERS20924463*
20861	*H. aberrans*	Honduras	2006-06-07	15.25000	-82.61667	ERS4141264
20862	*H. aberrans*	Honduras	2006-06-07	15.25000	-82.61667	ERS4141265
20864	*H. aberrans*	Honduras	2006-06-07	15.25000	-82.61667	ERS4141266
20866	*H. aberrans*	Honduras	2006-06-07	15.25000	-82.61667	ERS4141267
20867	*H. aberrans*	Honduras	2006-06-07	15.25000	-82.61667	ERS4141268
20892	*H. randallorum*	Honduras	2006-06-08	16.44500	-85.87500	ERS4141269
20893	*H. randallorum*	Honduras	2006-06-08	16.44500	-85.87500	ERS4141270
20894	*H. randallorum*	Honduras	2006-06-08	16.44500	-85.87500	ERS4141271
20896	*H. randallorum*	Honduras	2006-06-08	16.44500	-85.87500	ERS4141272
20922	*H. randallorum*	Honduras	2006-06-09	16.47361	-85.92389	ERS4141273
20923	*H. randallorum*	Honduras	2006-06-09	16.47361	-85.92389	ERS4141274
20980	*H. randallorum*	Honduras	2006-06-10	16.49750	-85.90278	ERS4141275
23301	*H. gummigutta*	Bocas del Toro, Pan.	2009-04-19	NA	NA	ERS20924464*
23318	*H. guttavarius*	Bocas del Toro, Pan.	2009-12-09	NA	NA	ERS20924465*
23322	*H. affinis*	Bocas del Toro, Pan.	2009-09-17	NA	NA	ERS20924466*
27698	*H. aberrans*	Quintana Roo, Mex.	2010-08-18	20.97881	-86.80011	ERS20924467*
27707	*H. unicolor*	Quintana Roo, Mex.	2010-08-19	20.84180	-86.87781	ERS20924468*
27709	*H. unicolor*	Quintana Roo, Mex.	2010-08-19	20.84180	-86.87781	ERS20924469*
27936	*H. atlahua*	Tamiahua, Mex.	2010-10-02	21.47032	-97.22894	ERS20924470*
28366	*H. gummigutta*	Bocas del Toro, Pan.	2017-02-04	9.33222	-82.22188	ERS8632035
28389	*H. aberrans*	Bocas del Toro, Pan.	2017-02-06	9.31810	-82.22180	ERS8632036
28393	*S. tortugarum*	Bocas del Toro, Pan.	2017-02-07	9.30140	-82.29410	ERS4141276
28713	*H. puella*	Quintana Roo, Mex.	2010-10-15	20.97689	-86.81816	ERS20924471*
28939	*H. puella*	Quintana Roo, Mex.	2010-11-30	20.84180	-86.87781	ERS20924472*
29137	*H. nigricans*	Quintana Roo, Mex.	2010-12-04	20.84180	-86.87781	ERS20924473*

Sample ID	Species	Location	Date	Latitude	Longitude	Accession
29212	*H. indigo*	Quintana Roo, Mex.	2010-12-04	20.86840	-86.84730	ERS20924474*
29213	*H. indigo*	Quintana Roo, Mex.	2010-12-04	20.86840	-86.84730	ERS20924475*
30001	*H. nigricans*	Quintana Roo, Mex.	2010-12-04	20.84180	-86.87781	ERS20924476*
33023	*H. nigricans*	Quintana Roo, Mex.	2010-12-04	20.84993	-86.87318	ERS20924477*
52988	*H. atlahua*	Tamiahua, Mex.	2013-06-25	21.47588	-97.22700	ERS20924478*
52989	*H. atlahua*	Tamiahua, Mex.	2013-06-25	21.47588	-97.22700	ERS20924479*
52990	*H. atlahua*	Tamiahua, Mex.	2013-06-25	21.47588	-97.22700	ERS20924480*
54649	*H. castroaguirrei*	Antón Lizardo, Mex.	2013-07-02	19.15555	-95.86390	ERS20924481*
54650	*H. castroaguirrei*	Antón Lizardo, Mex.	2013-07-02	19.15555	-95.86390	ERS20924482*
54689	*H. atlahua*	Antón Lizardo, Mex.	2013-07-02	19.17068	-95.87361	ERS20924483*
54761	*H. atlahua*	Antón Lizardo, Mex.	2013-06-29	19.08144	-95.96852	ERS20924484*
54786	*H. atlahua*	Antón Lizardo, Mex.	2013-06-29	19.09803	-95.98555	ERS20924485*
54849	*H. atlahua*	Antón Lizardo, Mex.	2013-06-30	19.05436	-95.83570	ERS20924486*
62263	*H. puella*	Arr. Alacranes, Mex.	2015-10-04	22.39949	-89.48939	ERS20924487*
62264	*H. puella*	Arr. Alacranes, Mex.	2015-10-04	22.39949	-89.48939	ERS20924488*
62515	*H. gemma*	Cayo Arcas, Mex.	2018-06-22	20.20523	-91.96972	ERS20924489*
62549	*H. ecosur*	Cayo Arcas, Mex.	2018-06-24	20.21088	-91.97582	ERS20924490*
62550	*H. aberrans*	Cayo Arcas, Mex.	2018-06-24	20.20394	-91.97077	ERS20924491*
62551	*H. aberrans*	Cayo Arcas, Mex.	2018-06-24	20.20394	-91.97077	ERS20924492*
62552	*H. aberrans*	Cayo Arcas, Mex.	2018-06-24	20.20394	-91.97077	ERS20924493*
62553	*H. gemma*	Cayo Arcas, Mex.	2018-06-24	20.20394	-91.97077	ERS20924494*
62555	*H. espinosai*	Cayo Arcas, Mex.	2018-06-24	20.20394	-91.97077	ERS20924495*
62556	*H. ecosur*	Cayo Arcas, Mex.	2018-06-24	20.20394	-91.97077	ERS20924496*
62558	*H. floridae*	Cayo Arcas, Mex.	2018-06-24	20.20394	-91.97077	ERS20924497*
62559	*H. floridae*	Cayo Arcas, Mex.	2018-06-24	20.20394	-91.97077	ERS20924498*
62560	*H. floridae*	Cayo Arcas, Mex.	2018-06-24	20.20394	-91.97077	ERS20924499*
62562	*H. randallorum*	Cayo Arcas, Mex.	2018-06-24	20.20394	-91.97077	ERS20924500*
62570	*H. gemma*	Cayo Arcas, Mex.	2018-06-25	20.20354	-91.97664	ERS20924501*
62571	*H. espinosai*	Cayo Arcas, Mex.	2018-06-25	20.20354	-91.97664	ERS20924502*
62575	*H. randallorum*	Cayo Arcas, Mex.	2018-06-25	20.20354	-91.97664	ERS20924503*
62576	*H. ecosur*	Cayo Arcas, Mex.	2018-06-25	20.20354	-91.97664	ERS20924504*
62577	*H. puella*	Cayo Arcas, Mex.	2018-06-25	20.20354	-91.97664	ERS20924505*
62585	*H. espinosai*	Cayo Arcas, Mex.	2018-06-25	20.19568	-91.95947	ERS20924506*
62948	*H. puella*	Cayo Arcas, Mex.	2018-06-19	20.20492	-91.97831	ERS20924507*
62953	*H. puella*	Cayo Arcas, Mex.	2018-06-19	20.20492	-91.97831	ERS20924508*
Bocas16.3	*S. tortugarum*	Bocas del Toro, Pan.	2016-02	NA	NA	ERS20924509*
Bocas16.4	*S. tortugarum*	Bocas del Toro, Pan.	2016-02	NA	NA	ERS20924510*
FL0318	*H. indigo*	Puerto Rico	2020-02-05	18.47923	-67.00593	SRR19070349

Sample ID	Species	Location	Date	Latitude	Longitude	Accession
FL0324	*H. sp1*	Puerto Rico	2020-02-05	18.47923	-67.00593	ERS20924511*
FL0331	*H. chlorurus*	Puerto Rico	2020-02-05	18.47923	-67.00593	SRR18184334
FL0835	*H. providencianus*	San Andrés, Col.	2016-06-06	12.50162	-81.73137	SRR17839752
FL0836	*H. guttavarius*	San Andrés, Col.	2016-06-06	12.50162	-81.73137	SRR18184176
FL0839	*H. aberrans*	San Andrés, Col.	2016-06-06	12.50162	-81.73137	SRR19070321
FL0880	*H. randallorum*	San Andrés, Col.	2016-06-08	12.83287	-82.22055	SRR17839729
HypoHaiti1	*H. liberte*	Haiti	2015-08-05	19.67700	-71.84300	ERS20924512*
HypoHaiti2	*H. liberte*	Haiti	2015-09-04	19.67700	-71.84300	ERS20924513*
HypoHaiti3	*H. liberte*	Haiti	2015-09-04	19.67700	-71.84300	ERS20924514*
PL17_101	*H. maya*	Belize	2017-06-04	16.77096	-88.16366	ERS2899592
PL17_111	*H. indigo*	Belize	2017-11-04	16.89555	-88.06139	ERS14948399
PL17_119	*H. maya*	Belize	2017-04-13	16.48726	-88.27183	ERS2899593
PL17_120	*H. maya*	Belize	2017-04-13	16.48726	-88.27183	ERS2899594
PL17_121	*H. maya*	Belize	2017-04-13	16.48726	-88.27183	ERS2899595
PL17_122	*H. maya*	Belize	2017-04-13	16.48726	-88.27183	ERS2899596
PL17_123	*H. maya*	Belize	2017-04-13	16.51037	-88.25462	ERS2899597
PL17_124	*H. maya*	Belize	2017-04-13	16.51037	-88.25462	ERS2899598
PL17_125	*H. randallorum*	Belize	2017-04-13	16.51037	-88.25462	ERS14948421
PL17_126	*H. maya*	Belize	2017-04-13	16.51037	-88.25462	ERS2899599
PL17_134	*H. unicolor*	Florida Keys, USA	2017-05-07	24.84680	-80.62296	ERS14948448
PL17_135	*H. unicolor*	Florida Keys, USA	2017-06-07	24.75258	-80.76065	ERS14948455
PL17_136	*H. unicolor*	Florida Keys, USA	2017-06-07	24.75258	-80.76065	ERS14948452
PL17_137	*H. unicolor*	Florida Keys, USA	2017-07-07	24.73489	-80.80080	ERS14948453
PL17_138	*H. unicolor*	Florida Keys, USA	2017-07-07	24.73489	-80.80080	ERS14948451
PL17_139	*H. puella*	Florida Keys, USA	2017-08-07	24.80618	-80.67677	ERS14948450
PL17_140	*H. unicolor*	Florida Keys, USA	2017-08-07	24.80618	-80.67677	ERS14948456
PL17_141	*H. unicolor*	Florida Keys, USA	2017-08-07	24.80618	-80.67677	ERS14948457
PL17_142	*H. gemma*	Florida Keys, USA	2017-08-07	24.80618	-80.67677	ERS2899137
PL17_143	*H. unicolor*	Florida Keys, USA	2017-09-07	24.81247	-80.66971	ERS14948454
PL17_144	*H. gemma*	Florida Keys, USA	2017-09-07	24.81247	-80.66971	ERS2899138
PL17_145	*H. gemma*	Florida Keys, USA	2017-09-07	24.81247	-80.66971	ERS2899139
PL17_148	*H. gemma*	Florida Keys, USA	2017-11-07	24.76937	-80.72791	ERS2899140
PL17_149	*H. nigricans*	Florida Keys, USA	2017-12-07	24.89978	-80.61716	ERS14948447
PL17_153	*H. gemma*	Florida Keys, USA	2017-07-13	24.80696	-80.67675	ERS2899141
PL17_155	*H. puella*	Florida Keys, USA	2017-07-14	25.04367	-80.36930	ERS14948449
PL17_157	*H. puella*	Florida Keys, USA	2017-07-15	24.72062	-80.83116	ERS14948446
PL17_159	*H. puella*	Florida Keys, USA	2017-07-16	24.68493	-80.92229	ERS14948445
PL17_160	*H. floridae*	Florida Keys, USA	2017-08-17	24.50768	-81.57143	ERS4141277

Sample ID	Species	Location	Date	Latitude	Longitude	Accession
PL17_21	*S. tigrinus*	Bocas del Toro, Pan.	2017-02-07	9.30140	-82.29410	ERS20924515*
PL17_23	*H. sp1*	Puerto Rico	2017-03-13	17.93425	-67.01878	ERS14948480
PL17_35	*H. indigo*	Puerto Rico	2017-03-15	17.95282	-67.05643	ERS14948482
PL17_37	*H. chlorurus*	Puerto Rico	2017-03-15	17.95282	-67.05643	ERS14948469
PL17_38	*H. chlorurus*	Puerto Rico	2017-03-15	17.95282	-67.05643	ERS14948464
PL17_39	*H. chlorurus*	Puerto Rico	2017-03-15	17.95282	-67.05643	ERS14948460
PL17_40	*H. chlorurus*	Puerto Rico	2017-03-15	17.95282	-67.05643	ERS14948466
PL17_41	*H. chlorurus*	Puerto Rico	2017-03-15	17.95282	-67.05643	ERS14948462
PL17_42	*H. chlorurus*	Puerto Rico	2017-03-15	17.95282	-67.05643	ERS14948467
PL17_43	*H. chlorurus*	Puerto Rico	2017-03-15	17.95282	-67.05643	ERS14948461
PL17_44	*H. chlorurus*	Puerto Rico	2017-03-15	17.95282	-67.05643	ERS14948463
PL17_50	*H. puella*	Puerto Rico	2017-03-16	17.95505	-67.05325	ERS14948475
PL17_53	*H. puella*	Puerto Rico	2017-03-16	17.95505	-67.05325	ERS14948476
PL17_54	*H. puella*	Puerto Rico	2017-03-16	17.95505	-67.05325	ERS14948483
PL17_55	*H. unicolor*	Puerto Rico	2017-03-16	17.95505	-67.05325	ERS14948485
PL17_56	*H. sp1*	Puerto Rico	2017-03-17	17.95787	-67.05804	ERS14948484
PL17_57	*H. puella*	Puerto Rico	2017-03-17	17.95787	-67.05804	ERS14948479
PL17_60	*H. puella*	Puerto Rico	2017-03-17	17.95787	-67.05804	ERS14948481
PL17_62	*H. chlorurus*	Puerto Rico	2017-03-17	17.95787	-67.05804	ERS14948465
PL17_63	*H. unicolor*	Puerto Rico	2017-03-18	17.97003	-67.04642	ERS14948487
PL17_64	*H. indigo*	Puerto Rico	2017-03-18	17.97003	-67.04642	ERS14948471
PL17_65	*H. puella*	Puerto Rico	2017-03-18	17.97003	-67.04642	ERS14948490
PL17_66	*H. unicolor*	Puerto Rico	2017-03-18	17.97003	-67.04642	ERS14948459
PL17_67	*H. unicolor*	Puerto Rico	2017-03-18	17.93425	-67.01878	ERS14948493
PL17_68	*H. guttavarius*	Puerto Rico	2017-03-20	17.89242	-67.01418	ERS14948470
PL17_69	*H. puella*	Puerto Rico	2017-03-21	17.89483	-67.01714	ERS14948486
PL17_70	*H. unicolor*	Puerto Rico	2017-03-21	17.89483	-67.01714	ERS14948489
PL17_71	*H. sp1*	Puerto Rico	2017-03-21	17.89483	-67.01714	ERS14948478
PL17_72	*H. sp1*	Puerto Rico	2017-03-21	17.89483	-67.01714	ERS14948472
PL17_73	*H. unicolor*	Puerto Rico	2017-03-21	17.89032	-67.01794	ERS14948491
PL17_74	*H. unicolor*	Puerto Rico	2017-03-21	17.89032	-67.01794	ERS14948492
PL17_75	*H. aberrans*	Puerto Rico	2017-03-22	NA	NA	ERS14948458
PL17_76	*H. sp1*	Puerto Rico	2017-03-22	NA	NA	ERS14948477
PL17_77	*H. unicolor*	Puerto Rico	2017-03-22	NA	NA	ERS14948488
PL17_79	*H. aberrans*	Puerto Rico	2017-03-22	NA	NA	ERS20924516*
PL17_82	*H. puella*	Puerto Rico	2017-03-23	NA	NA	ERS14948473
PL17_85	*H. indigo*	Puerto Rico	2017-03-24	NA	NA	ERS14948474
PL17_86	*H. chlorurus*	Puerto Rico	2017-03-24	17.95282	-67.05643	ERS14948468

**Sample ID**	**Species**	**Location**	**Date**	**Latitude**	**Longitude**	**Accession**
PL17_88	*H. aberrans*	Belize	2017-07-04	16.65361	-88.20097	ERS14948398
PL17_89	*H. maya*	Belize	2017-07-04	16.66000	-88.18500	ERS2899590
PL17_95	*H. maya*	Belize	2017-08-04	16.77096	-88.16366	ERS2899591
PL17_98	*H. indigo*	Belize	2017-09-04	16.80126	-88.07942	ERS20924517*
Rare1	*H. gemma*	Cayo Arenas, Mex.	2017-01-01	22.11528	-91.39833	ERS20924518*
Rare2	*H. aberrans*	Cayo Arenas, Mex.	2017-01-01	22.11528	-91.39833	ERS20924519*
Rare3	*H. puella*	Cayo Arenas, Mex.	2017-01-01	22.11528	-91.39833	ERS20924520*
s_tort_3	*S. tortugarum*	Bocas del Toro, Pan.	2016-02	NA	NA	ERS4141278

Table S2. Samples used for *de novo* genome assembly with long reads. All samples were collected in Bocas del Toro, Panama. Date, latitude and longitude refer to sampling dates and coordinates. Accession specifies GenBank assembly accessions numbers, at which assembly statistics can be found.

**Species**	**Assembly**	**Date**	**Latitude**	**Longitude**	**Accession**
H. puella	HypPue2.2	2022-02-10	9.252250	-82.130233	ERS20388951
H. nigricans	HypNig1.2	2022-03-01	9.302050	-82.205533	ERS20388952
H. unicolor	HypUni1.2	2022-03-01	9.302050	-82.205533	ERS20388953

Table S3. Samples used for DNA expression analysis. All samples were collected in Bocas del Toro, Panama. Date, latitude and longitude refer to sampling dates and coordinates.

**Sample ID**	**Species**	**Tissue**	**Date**	**Latitude**	**Longitude**	**Accession**
Pue_10	*H. puella*	retina	2017-02-09	9.367	-82.291	ERR2750999
Uni_04	*H. unicolor*	retina	2017-02-07	9.301	-82.294	ERR2751000
Nig_09	*H. nigricans*	retina	2017-02-07	9.301	-82.294	ERR2751001
Pue_05	*H. puella*	retina	2017-02-07	9.301	-82.294	ERR2751002
Nig_08	*H. nigricans*	retina	2017-02-07	9.301	-82.294	ERR2751003
Pue_04	*H. puella*	retina	2017-02-07	9.301	-82.294	ERR2751004
Pue_08	*H. puella*	retina	2017-02-09	9.367	-82.291	ERR2751005
Pue_09	*H. puella*	retina	2017-02-09	9.367	-82.291	ERR2751006
Uni_03	*H. unicolor*	retina	2017-02-06	9.318	-82.222	ERR2751007
Nig_05	*H. nigricans*	retina	2017-02-07	9.301	-82.294	ERR2751008
Nig_03	*H. nigricans*	retina	2017-02-06	9.318	-82.222	ERR2751009
Uni_02	*H. unicolor*	retina	2017-02-07	9.301	-82.294	ERR2751010
Nig_04	*H. nigricans*	retina	2017-02-07	9.301	-82.294	ERR2751011
Nig_07	*H. nigricans*	retina	2017-02-07	9.301	-82.294	ERR2751012
Pue_03	*H. puella*	retina	2017-02-07	9.301	-82.294	ERR2751013
Pue_02	*H. puella*	retina	2017-02-06	9.318	-82.222	ERR2751014
Nig_06	*H. nigricans*	retina	2017-02-06	9.318	-82.222	ERR2751015
Pue_07	*H. puella*	retina	2017-02-07	9.301	-82.294	ERR2751016
Uni_05	*H. unicolor*	retina	2017-02-07	9.301	-82.294	ERR2751017
Pue_06	*H. puella*	retina	2017-02-07	9.301	-82.294	ERR2751018
Uni_01	*H. unicolor*	retina	2017-02-06	9.318	-82.222	ERR2751019
Nig_01	*H. nigricans*	retina	2017-02-06	9.318	-82.222	ERR2751020
Pue_01	*H. puella*	retina	2017-02-06	9.318	-82.222	ERR2751021
Nig_02	*H. nigricans*	retina	2017-02-06	9.318	-82.222	ERR2751022
S52	*H. unicolor*	skin	2022-07-11	9.299082	-82.207186	*
S53	*H. puella*	skin	2022-07-10	9.299082	-82.207186	*
S54	*H. unicolor*	skin	2022-07-15	9.279863	-82.218015	*
S55	*H. puella*	skin	2022-07-14	9.279863	-82.218015	*
S56	*H. unicolor*	skin	2022-05-02	9.322150	-82.220150	*
S57	*H. unicolor*	skin	2022-07-19	9.332653	-82.199490	*
S58	*H. nigricans*	skin	2022-07-16	9.332653	-82.199490	*
S59	*H. nigricans*	skin	2022-07-14	9.279863	-82.218015	*
S60	*H. nigricans*	skin	2022-07-15	9.279863	-82.218015	*
S61	*H. puella*	skin	2022-07-14	9.279863	-82.218015	*

Sample ID	Species	Tissue	Date	Latitude	Longitude	Accession
S62	*H. nigricans*	skin	2022-05-02	9.322150	-82.220150	*
S63	*H. puella*	skin	2022-07-19	9.332653	-82.199490	*
S64	*H. unicolor*	skin	2022-07-09	9.299082	-82.207186	*
S65	*H. unicolor*	skin	2022-07-14	9.279863	-82.218015	*
S66	*H. nigricans*	skin	2022-07-09	9.299082	-82.207186	*
S67	*H. nigricans*	skin	2022-07-11	9.299082	-82.207186	*
S68	*H. unicolor*	skin	2022-05-02	9.322150	-82.220150	*
S69	*H. puella*	skin	2022-07-09	9.299082	-82.207186	*
S70	*H. puella*	skin	2022-07-16	9.332653	-82.199490	*
S71	*H. unicolor*	skin	2022-07-19	9.332653	-82.199490	*
S72	*H. nigricans*	skin	2022-07-19	9.332653	-82.199490	*
S73	*H. puella*	optic tectum	2022-07-10	9.299082	-82.207186	*
S74	*H. unicolor*	diencephalon	2022-07-11	9.299082	-82.207186	*
S75	*H. unicolor*	diencephalon	2022-07-19	9.332653	-82.199490	*
S76	*H. nigricans*	diencephalon	2022-07-14	9.279863	-82.218015	*
S77	*H. unicolor*	diencephalon	2022-07-15	9.279863	-82.218015	*
S78	*H. nigricans*	optic tectum	2022-07-09	9.299082	-82.207186	*
S79	*H. unicolor*	telencephalon	2022-07-14	9.279863	-82.218015	*
S80	*H. puella*	optic tectum	2022-07-09	9.299082	-82.207186	*
S81	*H. puella*	telencephalon	2022-07-14	9.279863	-82.218015	*
S82	*H. puella*	diencephalon	2022-07-10	9.299082	-82.207186	*
S83	*H. unicolor*	diencephalon	2022-07-09	9.299082	-82.207186	*
S84	*H. unicolor*	diencephalon	2022-07-14	9.279863	-82.218015	*
S85	*H. nigricans*	optic tectum	2022-07-15	9.279863	-82.218015	*
S86	*H. unicolor*	diencephalon	2022-07-19	9.332653	-82.199490	*
S87	*H. unicolor*	optic tectum	2022-07-19	9.332653	-82.199490	*
S88	*H. nigricans*	telencephalon	2022-07-16	9.332653	-82.199490	*
S89	*H. nigricans*	telencephalon	2022-07-15	9.279863	-82.218015	*
S90	*H. unicolor*	optic tectum	2022-07-14	9.279863	-82.218015	*
S91	*H. unicolor*	telencephalon	2022-07-19	9.332653	-82.199490	*
S92	*H. puella*	diencephalon	2022-07-14	9.279863	-82.218015	*
S93	*H. puella*	optic tectum	2022-07-16	9.332653	-82.199490	*
S94	*H. nigricans*	telencephalon	2022-07-11	9.299082	-82.207186	*
S95	*H. unicolor*	telencephalon	2022-07-11	9.299082	-82.207186	*
S96	*H. unicolor*	telencephalon	2022-07-19	9.332653	-82.199490	*
S97	*H. unicolor*	optic tectum	2022-07-15	9.279863	-82.218015	*
S98	*H. nigricans*	telencephalon	2022-07-14	9.279863	-82.218015	*
S99	*H. puella*	telencephalon	2022-07-10	9.299082	-82.207186	*

**Sample ID**	**Species**	**Tissue**	**Date**	**Latitude**	**Longitude**	**Accession**
S100	*H. nigricans*	diencephalon	2022-07-09	9.299082	-82.207186	*
S101	*H. puella*	telencephalon	2022-07-14	9.279863	-82.218015	*
S102	*H. nigricans*	optic tectum	2022-07-11	9.299082	-82.207186	*
S103	*H. nigricans*	telencephalon	2022-07-09	9.299082	-82.207186	*
S104	*H. nigricans*	optic tectum	2022-07-19	9.332653	-82.199490	*
S105	*H. puella*	telencephalon	2022-07-19	9.332653	-82.199490	*
S106	*H. nigricans*	optic tectum	2022-07-16	9.332653	-82.199490	*
S107	*H. puella*	optic tectum	2022-07-19	9.332653	-82.199490	*
S108	*H. unicolor*	telencephalon	2022-07-09	9.299082	-82.207186	*
S109	*H. unicolor*	optic tectum	2022-07-09	9.299082	-82.207186	*
S110	*H. puella*	telencephalon	2022-07-09	9.299082	-82.207186	*
S111	*H. unicolor*	optic tectum	2022-07-11	9.299082	-82.207186	*
S112	*H. puella*	telencephalon	2022-07-16	9.332653	-82.199490	*
S113	*H. nigricans*	telencephalon	2022-07-19	9.332653	-82.199490	*
S114	*H. nigricans*	diencephalon	2022-07-15	9.279863	-82.218015	*
S115	*H. nigricans*	diencephalon	2022-07-16	9.332653	-82.199490	*
S116	*H. puella*	optic tectum	2022-07-14	9.279863	-82.218015	*
S117	*H. puella*	diencephalon	2022-07-16	9.332653	-82.199490	*
S118	*H. nigricans*	diencephalon	2022-07-11	9.299082	-82.207186	*
S119	*H. puella*	optic tectum	2022-07-14	9.279863	-82.218015	*
S120	*H. unicolor*	telencephalon	2022-07-15	9.279863	-82.218015	*
S121	*H. puella*	diencephalon	2022-07-14	9.279863	-82.218015	*
S122	*H. puella*	diencephalon	2022-07-09	9.299082	-82.207186	*
S123	*H. puella*	diencephalon	2022-07-19	9.332653	-82.199490	*
S124	*H. nigricans*	diencephalon	2022-07-19	9.332653	-82.199490	*
S125	*H. unicolor*	optic tectum	2022-07-19	9.299082	-82.207186	*
S126	*H. nigricans*	optic tectum	2022-07-14	9.279863	-82.218015	*

Table S4. Demographic Inference with Linked Selection (DILS) results. Models with migration are favored over models with isolation (first column). Within models with migration, isolation with migration (IM, the two daughter populations continuously exchange alleles) is favored over a model with secondary contact (SC, the daughter populations evolve initially in isolation and then exchange alleles upon secondary contact, second column). Models with homogeneous effective population size and migration are favored over models with heterogeneous effective population size and migration (third and fourth column, respectively). PP: posterior probability.

	**Migration vs. Isolation**	**IM vs. SC**	**Mhomo vs. Mhetero**	**Nhomo vs. Nhetero**
Best model	migration	IM	Mhomo	Nhomo
Vote counts	860 | 140	788 | 212	846 | 154	619 | 381
PP	0.879	0.830	0.867	0.770

Table S5. Summary statistics of the six biogeographic models implemented in BioGeoBEARS based on Schemes 1 and 2 (first and second block separated by horizontal line; see Methods for details). LnL: Log-Likelihood, npar: number of parameters, d: dispersal, e: extinction, j: founder-speciation, w: dispersal matrix power exponential, AICc: corrected Akaike Information Criterion.

**Model**	**LnL**	**npar**	**d**	**e**	**j**	**w**	**AICc**	**AICc weight**
DEC	-108.5	2	0.028	1.00E-12	0	1	221.1	2.40E-17
DEC+j	-70.29	3	1.00E-12	1.00E-12	0.025	1	146.7	0.33
DIVALIKE	-101.7	2	0.034	1.00E-12	0	1	207.5	2.20E-14
DIVALIKE+j	-70.29	3	1.00E-12	1.00E-12	0.025	1	146.7	0.33
BAYAREALIKE	-143.5	2	0.033	0.077	0	1	291	1.60E-32
BAYAREALIKE+j	-70.29	3	1.00E-07	1.00E-07	0.025	1	146.7	0.33
DEC	-92.36	2	0.076	0.085	0	1	188.9	1.80E-18
DEC+j	-51.48	3	1.00E-12	1.00E-12	0.1	1	109.4	0.33
DIVALIKE	-86.54	2	0.087	0.03	0	1	177.3	6.10E-16
DIVALIKE+j	-51.48	3	1.00E-12	1.00E-12	0.099	1	109.4	0.33
BAYAREALIKE	-104.3	2	0.072	0.3	0	1	212.8	1.20E-23
BAYAREALIKE+j	-51.49	3	1.00E-07	1.00E-07	0.094	1	109.4	0.33

Table S6. General read statistics and number of samples per tissue for the DNA expression analysis. Asterisks denote datasets number of samples after removing outliers. M: million.

	**Retina**	**Brain**	**Skin**
No. of samples, *H. puella*	9*	18	6
No. of samples, *H. nigricans*	9	17*	7
No. of samples, *H. unicolor*	5	18	8
Raw read length	150 bp PE	100 bp PE	100 bp PE
Sum raw reads	157.7 M	1,676.7 M	353.1 M
Mean ± sd raw reads	6.6 ± 0.7 M	31.1 ± 8.4 M	16.8 ± 4.5 M
Mean ± sd trimmed read length	129 ± 9 bp	94 ± 1 bp	95 ± 1 bp
Sum trimmed reads	137.4 M	1,293.1 M	252.8 M
Mean ± sd trimmed reads	5.7 ± 0.7 M	24.0 ± 6.5 M	12.0 ± 3.6 M
Sum uniquely aligned reads	78.1 M	603.3 M	161.0 M
Mean ± sd uniquely aligned reads	3.3 ± 0.4 M	11.2 ± 2.9 M	7.7 ± 2.5 M
